# Synthesis and Antiradical Activity of Isoquercitrin Esters with Aromatic Acids and Their Homologues

**DOI:** 10.3390/ijms18051074

**Published:** 2017-05-17

**Authors:** Eva Heřmánková-Vavříková, Alena Křenková, Lucie Petrásková, Christopher Steven Chambers, Jakub Zápal, Marek Kuzma, Kateřina Valentová, Vladimír Křen

**Affiliations:** Laboratory of Biotransformation, Institute of Microbiology, Czech Academy of Sciences, Vídeňská 1083, CZ-142 20 Prague, Czech Republic; evavavrikova@seznam.cz (E.H.-V.); alenka.petrickova@gmail.com (A.K.); petraskova@biomed.cas.cz (L.P.); christopher.chambers@biomed.cas.cz (C.S.C.); jakub.zapal@seznam.cz (J.Z.); kuzma@biomed.cas.cz (M.K.); kren@biomed.cas.cz (V.K.)

**Keywords:** isoquercitrin, aromatic acid, gallic acid, cinnamic acid, antioxidant activity, Novozym 435, lipase, DPPH, lipoperoxidation

## Abstract

Isoquercitrin, (IQ, quercetin-3-*O*-β-d-glucopyranoside) is known for strong chemoprotectant activities. Acylation of flavonoid glucosides with carboxylic acids containing an aromatic ring brings entirely new properties to these compounds. Here, we describe the chemical and enzymatic synthesis of a series of IQ derivatives at the C-6″. IQ benzoate, phenylacetate, phenylpropanoate and cinnamate were prepared from respective vinyl esters using Novozym 435 (Lipase B from *Candida antarctica* immobilized on acrylic resin). The enzymatic procedure gave no products with “hydroxyaromatic” acids, their vinyl esters nor with their benzyl-protected forms. A chemical protection/deprotection method using Steglich reaction yielded IQ 4-hydroxybenzoate, vanillate and gallate. In case of *p*-coumaric, caffeic, and ferulic acid, the deprotection lead to the saturation of the double bonds at the phenylpropanoic moiety and yielded 4-hydroxy-, 3,4-dihydroxy- and 3-methoxy-4-hydroxy-phenylpropanoates. Reducing capacity of the cinnamate, gallate and 4-hydroxyphenylpropanoate towards Folin-Ciocalteau reagent was significantly lower than that of IQ, while other derivatives displayed slightly better or comparable capacity. Compared to isoquercitrin, most derivatives were less active in 1,1-diphenyl-2-picrylhydrazyl (DPPH) radical scavenging, but they showed significantly better 2,2′-azinobis-(3-ethylbenzothiazoline-6-sulfonic acid, ABTS) scavenging activity and were substantially more active in the inhibition of *tert*-butylhydroperoxide induced lipid peroxidation of rat liver microsomes. The most active compounds were the hydroxyphenylpropanoates.

## 1. Introduction

**Figure 1:**
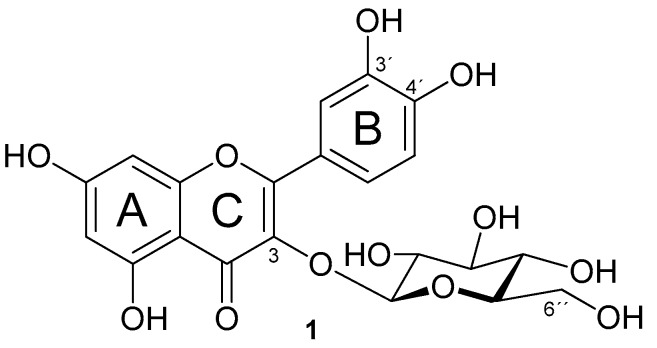
Isoquercitrin (**1**, quercetin 3-*O*-β-d-glucopyranoside).

Plant polyphenols are attractive for researchers and food producers due to their free radical scavenging and other, mostly positive, biological activities. Generally low toxicity of the polyphenols is another positive feature. One of the most popular flavonoids with a large number of health benefits is quercetin [1]. Some controversies concerning its possible mutagenic effects (positive Ames test) were closed and this substance now has a Generally Recognized As Safe (GRAS) status from the United States Food & Drug Administration (FDA) [2]. Its glucosylated form is isoquercitrin (**1**, IQ, quercetin-3-*O*-β-d-glucopyranoside, Figure 1), which can be found in fruits (apples, berries), vegetables (onions), medicinal plants (St. John’s worth) and in various plant-derived beverages (tea, wine) [3,4]. IQ is known as a strong chemoprotectant [4] against cardiovascular diseases [5], asthma [6], and diabetes [7]. Recently, an efficient method of biocatalytic production of pure IQ from rutin was developed [8,9] based on the use of thermophilic α-l-rhamnosidase [10] from *Aspergillus terreus* heterologously expressed in *Pichia pastoris*, which affords **1** in very high yields and purity (typically 97–99%).

Acylation of flavonoid glucosides with carboxylic acids containing an aromatic ring brings structural diversity to the flavonoid skeleton and often alters the physico-chemical (co-pigmentation, increased stability) and physiological (UV resistivity, radical scavenging capacity) properties of these natural and semi-synthetic molecules [11,12]. For example, the presence of *p*-coumaroyl-glucopyranosyl units in delphinidin-type anthocyanidins from *Dianella* berries was found to be responsible for their intensive blue color, at physiological pH values, due to co-pigmentation related bathochromic shift and pigment stabilization [13]. Similarly, chrysin glucoside acylated with methyl *p*-methoxycinnamic acid showed more efficient complexation of Al^3+^ and stronger binding to bovine serum albumin (BSA) than the parent compound [14]. Tiliroside (*p*-coumaroyl kaempferol glucoside) from *Tilia argentea* (Linden) exhibited a strong hepatoprotective effect against d-galactosamine/lipopolysaccharide induced liver injury in mice [15]. Also, a semi-synthetic introduction of aromatic acyl groups into the sugar moiety of IQ resulted in improved thermostability and light-sensitivity [11].

Acyl derivatives of flavonol glucosides can be prepared chemically or enzymatically. The use of chemical acylation methods generally proceeds via complex multistep synthesis with several protection/deprotection strategies, while the enzymatic-catalyzed reactions are more practical and straightforward due to regioselectivity at primary alcoholic groups on sugar moieties and mild reaction conditions. Different enzymes were used for acylation of IQ: *Candida antarctica* lipase B [16], subtilisin [17] or enzymatic reaction system from cultured cells of *Ipomoea batatas* [18]. Quercetin (isoquercitrin aglycone) is usually the starting material for chemical preparation of IQ or other *O*-β-d-glucosylated or *O*-β-d-glucuronidated quercetin derivatives [19]. The chemical synthesis of IQ coumaroyl was also initiated from quercetin [20].

In this work, we aimed to study chemical or chemoenzymatic modifications of IQ that lead to altered biological activity. We have prepared a complex panel of IQ derivatives **2**–**11**, substituted at C-6″ OH with carboxylic acids containing an aromatic ring (benzoic (**12**), phenylacetic (**13**), phenylpropanoic (**14**), cinnamic (**15**), 4-hydroxybenzoic (**16**), vanillic (**17**), gallic (**18**), *p*-coumaric (**19**), caffeic (**20**), ferulic (**21**)) using IQ as the starting material for both enzymatic and chemical approaches. The antiradical and anti-lipoperoxidant activities of all derivatives were determined.

## 2. Results and Discussion

### 2.1. Preparation of Isoquercitrin Esters (**2**–**5**) by Enzymatic Approach

**Figure 2:**
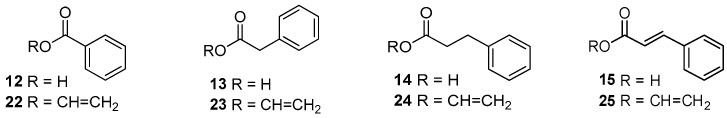
Aromatic acids **12**–**15** and their activated forms **22**–**25** used in the enzymatic approach [22,23].

**Scheme 1:**
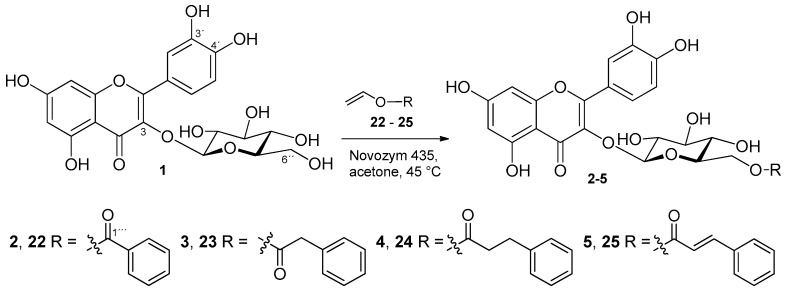
Preparation of isoquercitrin esters **2**–**5** by enzymatic methodology.

While the chemical modifications of flavonoids with numerous reactive hydroxyl groups require multistep protection/deprotection strategies, often including aggressive and toxic chemicals, lipases prefer stereoselective acylation of primary alcoholic groups [21]. Lipase-catalyzed preparation of C-6″ acyl derivatives of IQ (**2**–**5**) was accomplished by Novozym 435^®^—(*Candida antarctica* lipase CAL-B immobilized on an acrylic resin, Scheme 1). We have successfully used vinyl esters of benzoic, phenylacetic, phenylpropanoic, cinnamic acids **22**–**25** [22,23] (Figure 2), as donors, and therefore have achieved a faster lipase-catalyzed reaction with a stronger shift of reaction equilibrium towards the products **2**–**5**. The ratio between IQ and vinyl ester of respective aromatic acid was 1:5 in dry acetone.

IQ derivatives **2**, **4**, and **5** were prepared previously [11] using Chirazyme L-2 or Amano-PS as catalysts and vinyl esters of respective aromatic acids were used as acyl donors. However, formation of these products was confirmed only by ^13^C NMR and MS, but ^1^H NMR was missing. NMR and MS data are missing completely in the work of Nakajima et al. [18], who claimed the preparation of IQ cinnamate (**5**) with an enzymatic system from cultured cells of *Ipomoea batatas*. In contrast, we present here full structural data of all these esters (see Section 3.4.1 and Tables S1–S3 and Figures S1–S8 in the Supplementary Materials).

Chemoenzymatic preparation of isoquercitrin esters with both cinnamic, *p*-coumaric and ferulic acids has been described previously as a three-step synthesis [17]. First, methyl malonate was introduced to the primary OH of isoquercitrin in the presence of the protease subtilisin, followed by hydrolysis of the methoxycarbonyl group catalyzed by biophine esterase. The final step was the Knoevenagel reaction with the corresponding aldehyde, which condensed with malonic IQ monoester to afford IQ *p*-coumarate or ferulate.

**Figure 3:**
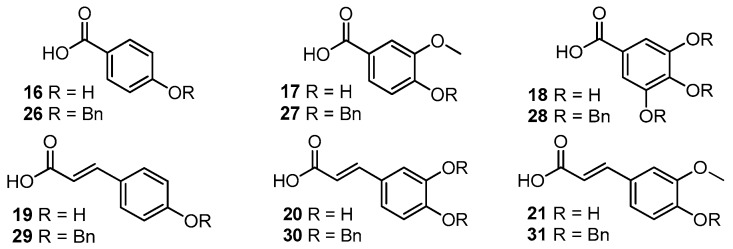
Aromatic acids **16**–**21** and their protected forms **26**–**31** used for the chemical approach.

To obtain IQ esters with acids containing a hydroxyaromatic moiety (4-hydroxybenzoic, vanillic, gallic, *p*-coumaric, caffeic, and ferulic, **16**–**21**, Figure 3) CAL-B catalyzed reactions (up to 12 days, 45 °C) were tested with intact acids, their vinyl esters, acetyl-protected vinyl esters and benzyl-protected acids. However, the enzymatic reactions did not afford any products with IQ and the non-protected acids **16**–**21**. In contrast to the previously published results with *p*-coumaric acid (no spectroscopic data given at all) [24], vinylesters of the acids **16**–**21** did not afford the expected products with IQ (Novozym 435, up to 12 days, 45 °C). When the aromatic OHs were protected by acetylation, the vinyl esters yielded with isoquercitrin (Novozym 435, 2 days, 45 °C) only undesired isoquercitrin 3″,6″-di-*O*-acetate (confirmed by MS and NMR). Using the benzyl-protected 4-hydroxybenzoic (**26**) [25], vanillic (**27**) [26], gallic (**28**) [27], *p*-coumaric (**29**), caffeic (**30**) [28] and ferulic (**31**) [29] acids, prepared according to the previous procedures (Figure 3, Section 2.2), enzymatic reactions (2 days, 45 °C) were also unsuccessful, in this case probably due to bulk acyl donors. Chemoenzymatic synthesis is therefore not completely versatile and IQ esters with aromatic acids **16**–**21** and therefore, their esters could not be prepared by this method. Thus, the chemical approach for compound **6**–**11** synthesis was chosen.

### 2.2. Preparation of Isoquercitrin Esters (**6**–**11**) by Chemical Approach

**Scheme 2:**
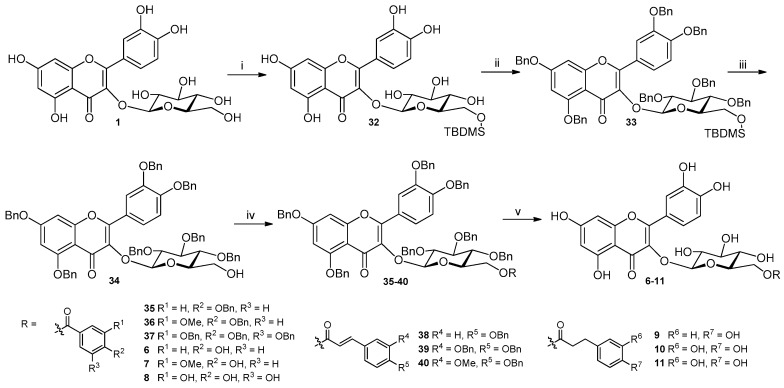
Preparation of isoquercitrin esters **6**–**11** by chemical methodology. Reagents and conditions: (i) imidazole, *tert*-butyldimethylsilyl chloride, dimethylformamide (DMF), 25 °C, 24 h; (ii) NaH, BnBr, DMF, 25 °C, 24 h; (iii) tetrabutylammonium fluoride , tetrahydrofuran (THF) 25 °C, 24 h; (iv) perBn aromatic acids **26**–**31**, 4-dimethylaminopyridine , *N*,*N*′-dicyclohexylcarbodiimide (DCC), 25 °C, 24 h; (v) H_2_-Pd/C, EtOH:THF 1:1, 25 °C, 12 h.

Protection of the phenolic OH groups of the acids **16**–**21** (see Section 2.1) and of IQ (**1**) was required for the chemical synthesis of IQ esters **6**–**11**. Selective protection of complex polyphenols, such as isoquercitrin, is complicated and protection groups have to be chosen very carefully due to the presence of many hydroxy- groups with different reactivity. First, the primary alcoholic group of glucose was selectively protected using *tert*-butyldimethylsilyl chloride (TBDMSCl) in the presence of imidazole. Silylated isoquercitrin **32** was then benzylated with benzyl bromide to afford **33** in 37% yield. Fully protected isoquercitrin **33** was desilylated with tetrabutylammonium fluoride to afford **34** in 82% yield. Protection with benzoyl group (BzCl) proved to be impracticable due to the migration of Bz groups to C-6″ OH after desilylation (Scheme 2).

**Figure 4:**
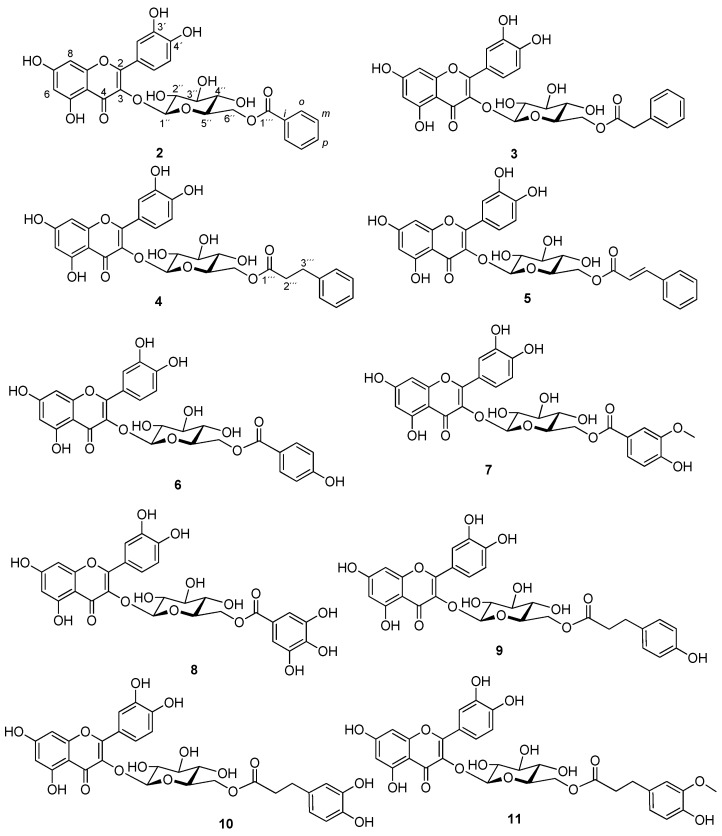
Proposed structures of the isoquercitrin esters **2**–**11**.

Free C-6″ OH of compound **34** was then esterified by the Steglich reaction (*N*,*N*′-dicyclohexylcarbodiimide (DCC), 4-dimethylaminopyridine (DMAP)) with acids **26**–**31** yielding respective intermediates **35**–**40**. Final deprotection was accomplished by hydrogenolysis with Pd-C yielding **6**–**11** (Figure 4). In the case of compounds **6**–**8**, their expected structures were confirmed by NMR. NMR and MS have revealed that during hydrogenolytic debenzylation of **38**–**40**, the double bonds at the phenylpropenoic moieties were hydrogenated forming the saturated products **9**–**11** (Scheme 2).

Following methods for selective deprotection of compounds **38**–**40** were tested to preserve the double bond: (1) Inspired by partial successful preparation of isoquercitrin *p*-coumarate from perbenzylated intermediate [20], preservation of the double bond via catalytic hydrogenation using 1,4-cyclohexadiene/Pd-C was tried. However, debenzylation failed even after 48 h; (2) TiCl_4_ in dry DCM at −20 °C under Ar for 1 h, previously used for synthesis of 1,2,3,4,6-penta-caffeoyl-d-glucopyranose with intact olefinic bonds [30], gave only the saturated product **10** in our hands; (3) Different polyhydroxycinnamic acids were reported to be debenzylated using AlCl_3_/DMAP in dry DCM (0 °C, 48 h) [31], debenzylation of our compound **39** using this approach was unsuccessful as no reaction occurred. Therefore, we were unable to obtain respective cinnamic acid derivatives. Nevertheless, the compounds **9**–**11** (Figure 4) obtained have not been reported to date, isoquercitrin 4-hydroxyphenylpropanoate (**9**), 3,4-dihydroxyphenylpropanoate (**10**) and 4-hydroxy-3-methoxyphenylpropanoate (**11**) were thus included into our panel of isoquercitrin derivatives with aromatic acids and their homologues for the determination of their antioxidant activity.

### 2.3. Antiradical Activity

Complex antioxidant activity of all isoquercitrin esters **2**–**11** (Figure 4) was evaluated and compared with that of isoquercitrin (**1**, Table 1). The reducing capacity of IQ cinnamate (**5**), gallate (**8**) and 4-hydroxyphenylpropanoate (**9**) towards Folin-Ciocalteau reagent (FCR), which is performed in aqueous milieu at alkaline pH, was approximately half of that of **1** (0.5 vs. 1.1 gallic acid equivalents, GAE). This is probably related to the increased lipophilicity of these compounds. On the other hand, IQ phenylacetate (**3**), phenylpropanoate (**4**) vanillate (**7**) and 4-hydroxy-3-methoxyphenylpropanoate (**11**) displayed slightly better (1.5 GAE) reducing capacity than isoquercitrin and the rest of the derivatives were not significantly different from **1**. Compared to isoquercitrin, compounds **3**, **9**, and **10** had comparable activity (IC_50_, i.e., the concentration exhibiting 50% effect, of 1.4–1.9 µM) in 1,1-diphenyl-2-picrylhydrazyl (DPPH) radical scavenging and all other derivatives were less active (IC_50_ 2.2–11.5 µM with compound **6** being the least effective). In contrast, most of the derivatives showed significantly better ABTS scavenging activity than isoquercitrin (1.98 trolox equivalents, TE). The loss of activity was observed only in the case of IQ phenylacetate (**3**) and cinnamate (**5**). Compounds **2**, **7**, **9**, **10** and **11** displayed the strongest activity (over 7 trolox equivalents (TE)), followed by IQ gallate (**8**) with 5.7 TE. Finally, isoquercitrin derivatives, except for **5**, were substantially more active than isoquercitrin (IC_50_ = 972 µM) in the inhibition of lipid peroxidation of rat liver microsomes induced by *tert*-butylhydroperoxide. Compounds **8** (IC_50_ = 6.6 µM) and **3** (IC_50_ = 6.8 µM) were the most active, but strong activity was noted also for originally unwanted compounds **9**–**11** (IC_50_ ≈ 9.4 µM). These results are most likely influenced by the spatial arrangement of the aromatic moieties in the presence of the water-lipid interphase [32].

The results obtained for isoquercitrin are in a good agreement with our previous study [16]. From the compounds described in the present work, some antioxidant activity was previously reported only for IQ-6″-gallate and isolated from *Euphorbia supina* [33] and *Pemphis acidula* [34], respectively. Peroxynitrite (ONOO^−^) [33] and DPPH scavenging activity, inhibition of methyl linoleate oxidation and inhibition of oxidative cell death of IQ-6″-gallate was enhanced compared to IQ [34]. This is in accordance with the present study. Isomeric IQ-2″-gallate, isolated from *Persicatia lapathifolia* inhibited peroxynitrite and superoxide production from RAW 264.7 macrophages, but its effect was compared only with quercetin (which was more efficient) and not isoquercitrin [35]. Compared to previously reported isoquercitrin esters with mono- and dicarboxylic aliphatic acids from our laboratory [16], the derivatives containing an aromatic ring presented here generally displayed similar DPPH scavenging potency and were less able to reduce FCR. On the other hand, the esters **2**, **4** and **7**–**11** especially exhibited more pronounced ABTS scavenging and proved to be much stronger inhibitors against lipid peroxidation.

## 3. Materials and Methods

### 3.1. Chemicals and Reagents

Isoquercitrin (purity 96%) was prepared by enzymatic deglycosylation of rutin using our procedure [8,9]. Lipase B from *Candida antarctica* immobilized on acrylic resin (Novozym 435) was purchased from Novo-Nordisk (Copenhagen, Denmark). Folin-Ciocalteau reagent was purchased from Merck (Prague, Czech Republic). Benzoic (**12**), phenylacetic (**13**), phenylpropanoic (**14**), cinnamic (**15**), 4-hydroxybenzoic (**16**), vanillic (**17**), gallic (**18**), *p*-coumaric (**19**), caffeic (**20**) and ferulic (**21**) acids, 1,1-diphenyl-2-picrylhydrazyl (DPPH) radical, antioxidant assay kit (CS0790); pooled microsomes from male rat liver (M9066); trolox and other chemicals were obtained from Sigma-Aldrich (Prague, Czech Republic). Vinyl esters (**12**, **15**) [22], (**13**, **14**) [23], and benzylated acids **16** [25], **17** [26], **18** [27], **19**, **20** [28], and **21** [29] were prepared as described previously.

### 3.2. NMR and MS Methods

NMR spectra were recorded on a Bruker Avance III 700 MHz spectrometer (700.13 MHz for ^1^H, 176.05 MHz for ^13^C), a Bruker Avance III 600 MHz spectrometer (600.23 MHz for ^1^H, 150.93 MHz for ^13^C) and Bruker Avance III 400 MHz (400.00 MHz for ^1^H, 100.59 MHz for ^13^C, all spectrometers from Bruker BioSpin, Rheinstetten, Germany) in DMSO-*d*_6_ (99.8% atom D) at 30 °C or acetone-*d*_6_, (99.8% atom D, VWR International, Stříbrná Skalice, Czech Republic) at 20 °C. The residual signal of the solvent was used as an internal standard (for DMSO-*d*_6_: δ_H_ 2.500 ppm, δ_C_ 39.60 ppm, for acetone-*d*_6_: δ_H_ 2.050 ppm, δ_C_ 30.50 ppm). The following experiments were performed and processed using the manufacturer’s software (Topspin 3.2, Bruker BioSpin, Rheinstetten, Germany): ^1^H NMR, ^13^C NMR with power-gated ^1^H decoupling, ge-2D COSY, ge-2D multiplicity-edited ^1^H–^13^C HSQC, ge-2D ^1^H–^13^C HMBC and 1D TOCSY. ^1^H NMR and ^13^C NMR spectra were zero filled to four-fold data points and multiplied by a window function before the Fourier transformation.

The two-parameter double-exponential Lorentz–Gauss function was applied for ^1^H to improve resolution, and line broadening (1 Hz) was applied to get a better ^13^C signal-to-noise ratio. The multiplicity of the ^13^C signals was resolved using the ge-2D multiplicity-edited ^1^H–^13^C HSQC. Chemical shifts are given in δ-scale with digital resolution justifying the reported values to three decimal places for δ_H_ or two decimal places for δ_C_.

Mass spectra were obtained using the Shimadzu Prominence system (Shimadzu, Kyoto, Japan) equipped with an electrospray ion source. The samples were dissolved in acetonitrile and introduced into the mobile phase flow (acetonitrile, 0.4 mL/min).

### 3.3. HPLC Analysis

All analytical HPLC analyses were performed with the Shimadzu Prominence System (Shimadzu Corporation, Kyoto, Japan) consisting of a DGU-20A_3_ mobile phase degasser, two LC-20AD solvent delivery units, a SIL-20AC cooling auto sampler, a CTO-10AS column oven and SPD-M20A diode array detector. The Chromolith Performance RP-18e monolithic column (100 mm × 3 mm i.d., Merck, DE) coupled with a guard column (5 mm × 4.6 mm i.d., Merck, DE) was employed. Mobile phase acetonitrile/water/formic acid (5/95/0.1, *v*/*v*/*v*, phase A) and acetonitrile/water/formic acid (80/20/0.1, *v*/*v*/*v*, phase B) were used for the analyses; gradient: 0–10 min 7–80% B; 10–12 min 80% B, 12–14 min 80–7% B at 25 °C; the flow rate was 1.2 mL/min. The PDA data were acquired in the 200–450 nm range and signals at 360 nm were extracted. Chromatographic data were collected and processed using Shimadzu Solution software (version 5.75 SP2, Shimadzu Corporation, Tokyo, Japan) at a rate of 40 Hz and detector time constant of 0.025 s.

### 3.4. Chemistry

#### 3.4.1. Synthesis of Compounds **2**–**5**

Dried isoquercitrin (**1**, 300 mg, 0.65 mmol, 1 equivalents (eq.)) and vinyl ester of the respective acid (**22**–**25**, 3.23 mmol, 5 eq.) were dissolved in anhydrous acetone (15 mL). Novozym 435^®^ (400 mg) and molecular sieves Å4 (300 mg) were added to the solution. The reaction mixture was incubated at 45 °C, 220 rpm. Part of IQ dissolved gradually during the reaction, which was terminated after 12 days by filtering off the enzyme and the solvent was evaporated *in vacuo*. The crude product was purified by silica gel flash chromatography (chloroform/methanol 95:5).

**Figure 5:**
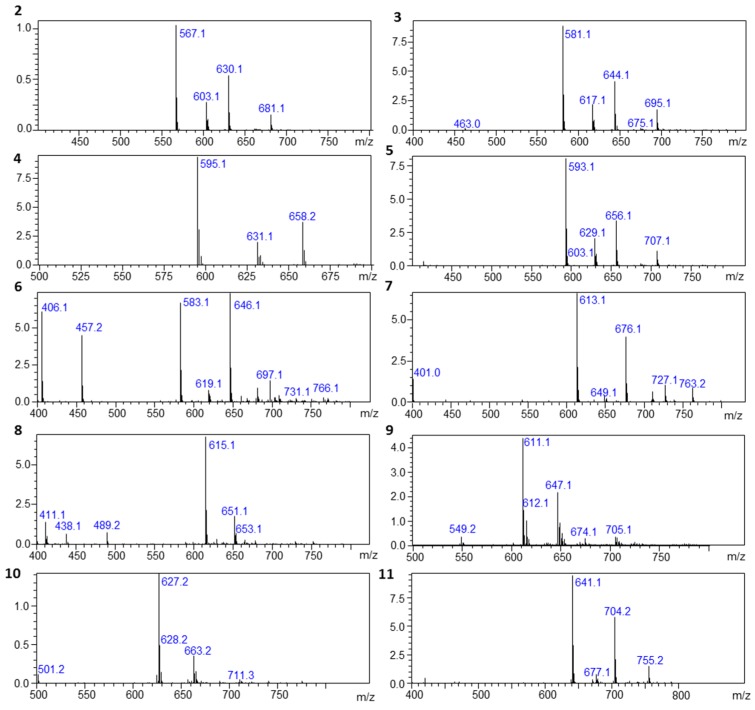
MS-ESI spectra of the isoquercitrin esters **2**–**11**.

*((2*R*,3*S*,4*S*,5*R*,6*S*)-6-((2-(3,4-Dihydroxyphenyl)-5,7-dihydroxy-4-oxo-4*H*-chromen-3-yl)oxy)-3,4,5-trihydroxytetrahydro-2*H*-pyran-2-yl)methyl benzoate* (**2**, IQ benzoate): Yellow solid (yield 29%, 106 mg, 0.19 mmol). For ^1^H and ^13^C NMR data see Tables S1, S2 and Figures S1, S2 in the Supplementary Materials. MS-ESI *m*/*z*: [M − H]^−^ calcd for C_28_H_23_O_13_ 567.1; found 567.1 (Figure 5).

*((2*R*,*3S*,4*S*,5*R*,6*S*)-6-((2-(3,4-Dihydroxyphenyl)-5,7-dihydroxy-4-oxo-4*H*-chromen-3-yl)oxy)-3,4,5-trihydroxytetrahydro-2*H*-pyran-2-yl)methyl 2-phenylacetate* (**3**, IQ phenylacetate): Yellow solid (35%, 133 mg, 0.23 mmol). For ^1^H and ^13^C NMR data see Tables S1, S2 and Figures S3, S4 in the Supplementary Materials. MS-ESI *m*/*z*: [M − H]^−^ calcd for C_29_H_25_O_13_ 581.1; found 581.1 (Figure 5).

*((2*R*,3*S*,4*S*,5*R*,6*S*)-6-((2-(3,4-Dihydroxyphenyl)-5,7-dihydroxy-4-oxo-4*H*-chromen-3-yl)oxy)-3,4,5-trihydroxytetrahydro-2*H*-pyran-2-yl)methyl 3-phenylpropanoate* (**4**, IQ phenylpropanoate): Yellow solid (yield 33%, 128 mg, 0.22 mmol). For ^1^H and ^13^C NMR data see Tables S1, S3 and Figures S5, S6 in the Supplementary Materials. MS-ESI *m*/*z*: [M − H]^−^ calcd for C_30_H_27_O_13_ 595.1; found 595.1 (Figure 5).

*((2*R*,3*S*,4*S*,5*R*,6*S*)-6-((2-(3,4-Dihydroxyphenyl)-5,7-dihydroxy-4-oxo-4*H*-chromen-3-yl)oxy)-3,4,5-trihydroxytetrahydro-2*H*-pyran-2-yl)methyl cinnamate* (**5**, IQ cinnamate): Yellow solid (yield 39%, 152 mg, 0.26 mmol). For ^1^H and ^13^C NMR data see Tables S1, S2 and Figures S7, S8 in the Supplementary Materials. MS-ESI *m*/*z*: [M − H]^−^ calcd for C_30_H_25_O_13_ 593.1; found: 593.1 (Figure 5).

#### 3.4.2. Synthesis of Compounds **32**–**34** (Structures in Figure S21 in the Supplementary Materials)

*3-(((2*S*,3*R*,4*S*,5*S*,6*R*)-6-(((*tert*-Butyldimethylsilyl)oxy)methyl)-3,4,5-trihydroxytetrahydro-2*H*-pyran-2-yl)oxy)-2-(3,4-dihydroxyphenyl)-5,7-dihydroxy-4*H*-chromen-4-one* (**32**): To a stirred solution of **1** (2.0 g, 4.31 mmol) and imidazole (586.6 mg, 8.61 mmol, 2.0 eq.) in dry DMF (20 mL) at 0 °C and under Ar, a solution of TBDMSCl (1.3 g, 8.63 mmol, 2.0 eq.) in DMF (20 mL) was added dropwise. After stirring at RT for 17 h the reaction mixture was diluted with H_2_O (100 mL) and extracted with EtOAc (3 × 300 mL). Combined organic layers were dried over anhydrous Na_2_SO_4_, filtered and concentrated *in vacuo*. The residue was purified by column chromatography using gradient (EtOAc/MeOH from 50:1 to 25:1) to produce **32** as yellow oil (yield 82%, 2.0 g, 3.46 mmol). For ^1^H and ^13^C NMR data see Tables S7, S8 and Figures S22, S23 in the Supplementary Materials. MS-ESI *m*/*z*: [M − H]^−^ calcd for C_27_H_33_O_12_Si 577.2; found 577.2.

*5,7-*bis*(Benzyloxy)-2-(3,4-*bis*(benzyloxy)phenyl)-3-(((2*S,*3*R,*4*S,*5*R,*6*R*)-3,4,5-*tris*(benzyloxy)-6-(((tert-butyldimethylsilyl)oxy)methyl)tetrahydro-2*H*-pyran-2-yl)oxy)-4*H*-chromen-4-one* (**33**): To an argon-purged solution of **32** (2.0 g, 3.46 mmol) and NaH (60% suspension in mineral oil, 2.3 g, 57.5 mmol, 16.6 eq.) in dry DMF (80 mL) at 0 °C, a solution of BnBr (29.0 mL, 24.38 mmol, 7.0 eq.) was added dropwise. The solution was stirred at RT for 24 h and then extracted with EtOAc (3 × 300 mL). The combined organic layers were dried over anhydrous Na_2_SO_4_, filtered and concentrated *in vacuo*. The residue was purified by column chromatography (toluene/EtOAc = 20:1) to produce title compound as a brownish oil (yield 37%, 1.5 g, 1.24 mmol). For ^1^H and ^13^C NMR data see Tables S7, S8 and Figures S24, S25 in the Supplementary Materials. MS-ESI *m*/*z*: [M + H]^+^ calcd for C_76_H_77_O_12_Si 1209.5; found 1209.5.

*5,7-*bis*(Benzyloxy)-2-(3,4-*bis*(benzyloxy)phenyl)-3-(((2*S*,3*R*,4*S*,5*R*,6*R*)-3,4,5-*tris*(benzyloxy)-6-(hydroxyme-thyl)tetrahydro-2*H*-pyran-2-yl)oxy)-4*H*-chromen-4-one* (**34**): To an argon-purged flask containing a solution of **33** (1.5 g, 1.24 mmol) in anhydrous THF (30 mL) at 0 °C a solution of tetrabutylammonium fluoride in THF (1 M, 7.8 mL) was added dropwise. Reaction mixture was stirred under an Ar atmosphere at 0 °C for 30 min and then at RT for next 17 h. The reaction mixture was diluted with H_2_O (100 mL) and then extracted with EtOAc (3 × 300 mL). The combined organic layers were dried over anhydrous Na_2_SO_4_, filtered and concentrated *in vacuo*. The residue was purified by column chromatography (toluene/EtOAc = 10:1) to yield yellow oil (82%, 1.1 g, 1.01 mmol). For ^1^H and ^13^C NMR data see Tables S7, S8 and Figures S26, S27 in the Supplementary Materials. MS-ESI *m*/*z*: [M + H]^+^ calcd for C_70_H_63_O_12_ 1095.4; found 1095.4.

#### 3.4.3. Synthesis of Compounds **35**–**40** (Structures in Figure S21 in the Supplementary Materials)

Anhydrous CH_2_Cl_2_ (10 mL) was added to a flask containing **34** (200 mg, 0.18 mmol, 1 eq.), benzyl-protected acids **26**–**31** (0.46 mmol, 2.5 eq.), DCC (76 mg, 0.36 mmol, 2 eq.) and DMAP (11 mg, 0.09 mmol, 0.5 eq.) and the reaction mixture was stirred at RT for 48 h. Then solvent was evaporated *in vacuo* and the residue was purified by column chromatography using toluene/EtOAc (99:1) and products **35**–**40** were isolated.

*((2*R*,3*R*,4*S*,5*R*,6*S*)-3,4,5-*tris*(Benzyloxy)-6-((5,7-*bis*(benzyloxy)-2-(3,4-*bis*(benzyloxy)phenyl)-4-oxo-4*H*-chromen-3-yl)oxy)tetrahydro-2*H*-pyran-2-yl)methyl 4-(benzyloxy)benzoate* (**35**): Yellow solid (yield 75%, 180 mg, 0.14 mmol). For ^1^H and ^13^C NMR data see Tables S9, S10 and Figures S28, S29 in the Supplementary Materials. MS-ESI *m*/*z*: [M + H]^+^ calcd for C_84_H_73_O_14_ 1305.5; found 1305.7.

*((2*R*,3*R*,4*S*,5*R*,6*S*)-3,4,5-*tris*(Benzyloxy)-6-((5,7-*bis*(benzyloxy)-2-(3,4-*bis*(benzyloxy)phenyl)-4-oxo-4*H*-chromen-3-yl)oxy)tetrahydro-2*H*-pyran-2-yl)methyl 4-(benzyloxy)-3-methoxybenzoate* (**36**): Yellow solid (yield 72%, 175 mg, 0.13 mmol). For ^1^H and ^13^C NMR data see Tables S9, S10 and Figures S30, S31 in the Supplementary Materials. MS-ESI *m*/*z*: [M + H]^+^ calcd for C_85_H_75_O_15_ 1335.5; found 1335.8.

*((2*R*,3*R*,4*S*,5*R*,6*S*)-3,4,5-*tris*(Benzyloxy)-6-((5,7-*bis*(benzyloxy)-2-(3,4-*bis*(benzyloxy)phenyl)-4-oxo-4*H*-chromen-3-yl)oxy)tetrahydro-2*H*-pyran-2-yl)methyl 3,4,5-tris(benzyloxy)benzoate* (**37**): Yellow solid (yield 61%, 170 mg, 0.11 mmol). For ^1^H and ^13^C NMR data see Tables S9, S11 and Figures S32, S33 in the Supplementary Materials. MS-ESI *m*/*z*: [M + H]^+^ calcd for C_98_H_85_O_16_ 1517.6; found 1517.9.

*(*E*)-((2*R*,3*R*,4*S*,5*R*,6*S*)-3,4,5-*tris*(Benzyloxy)-6-((5,7-*bis*(benzyloxy)-2-(3,4-*bis*(benzyloxy)phenyl)-4-oxo-4*H*-chromen-3-yl)oxy)tetrahydro-2*H*-pyran-2-yl)methyl 3-(4-(benzyloxy)phenyl)acrylate* (**38**): Yellow solid (yield 70%, 172 mg, 0.13 mmol). For ^1^H and ^13^C NMR data see Tables S9, S11 and Figures S34, S35 in the Supplementary Materials. MS-ESI *m*/*z*: [M + Na]^+^ calcd for C_86_H_74_O_14_Na 1353.5; found 1353.7.

*(*E*)-((2*R*,3*R*,4*S*,5*R*,6*S*)-3,4,5-*tris*(Benzyloxy)-6-((5,7-*bis*(benzyloxy)-2-(3,4-*bis*(benzyloxy)phenyl)-4-oxo-4*H*-chromen-3-yl)oxy)tetrahydro-2*H*-pyran-2-yl)methyl 3-(3,4-*bis*(benzyloxy)phenyl)acrylate* (**39**): Yellow *solid* (yield 86%, 227 mg, 0.16 mmol). For ^1^H and ^13^C NMR data see Tables S12, S13 and Figures S36, S37 in the Supplementary Materials. MS-ESI *m*/*z*: [M + H]^+^ calcd for C_93_H_81_O_15_ 1437.6; found 1437.7.

*(*E*)-((2*R*,3*R*,4*S*,5*R*,6*S*)-3,4,5-*tris*(Benzyloxy)-6-((5,7-*bis*(benzyloxy)-2-(3,4-*bis*(benzyloxy)phenyl)-4-oxo-4*H*-chromen-3-yl)oxy)tetrahydro-2*H*-pyran-2-yl)methyl 3-(4-(benzyloxy)-3-methoxyphenyl)acrylate* (**40**): Yellow solid (yield 64%, 160 mg, 0.12 mmol). For ^1^H and ^13^C NMR data see Tables S12, S13 and Figures S38, S39 in the Supplementary Materials. MS-ESI *m*/*z*: [M + H]^+^ calcd for C_87_H_77_O_15_ 1362.5; found 1361.7.

#### 3.4.4. Synthesis of Compounds **6**–**11**

Hydrogenolysis of **35**–**40** (200 mg) was performed with Pd/C-H_2_ (200 mg, 10% *w*/*w*) in EtOH/THF 1:1 (40 mL) and was stirred for 12 h. Then reaction mixtures were filtrated and evaporated *in vacuo* to yield **6**–**11**.

*((2*R*,3*S*,4*S*,5*R*,6*S*)-6-((2-(3,4-Dihydroxyphenyl)-5,7-dihydroxy-4-oxo-4*H*-chromen-3-yl)oxy)-3,4,5-trihydroxytetrahydro-2*H*-pyran-2-yl)methyl 4-hydroxybenzoate* (**6**, IQ 4-OH benzoate): Yellow solid (yield 91%, 74 mg, 0.13 mmol). For ^1^H and ^13^C NMR data see Tables S4, S5 and Figures S9, S10 in the Supplementary Materials. MS-ESI *m*/*z*: [M − H]^−^ calcd for C_28_H_23_O_14_ 583.1; found 583.1 (Figure 5).

*((2*R*,3*S*,4*S*,5*R*,6*S*)-6-((2-(3,4-Dihydroxyphenyl)-5,7-dihydroxy-4-oxo-4*H*-chromen-3-yl)oxy)-3,4,5-trihydroxytetrahydro-2*H*-pyran-2-yl)methyl 4-hydroxy-3-methoxybenzoate* (**7**, IQ vanillate): Yellow solid (yield 60%, 50 mg, 0.08 mmol). For ^1^H and ^13^C NMR data see Tables S4, S5 and Figures S11, S12 in the Supplementary Materials. MS-ESI *m*/*z*: [M − H]^−^ calcd for C_29_H_25_O_15_ 613.1; found 613.1 (Figure 5).

*((2*R*,3*S*,4*S*,5*R*,6*S*)-6-((2-(3,4-Dihydroxyphenyl)-5,7-dihydroxy-4-oxo-4*H*-chromen-3-yl)oxy)-3,4,5-trihydroxytetrahydro-2*H*-pyran-2-yl)methyl 3,4,5-trihydroxybenzoate* (**8**, IQ gallate): Yellow solid (yield 75%, 60 mg, 0.10 mmol). For ^1^H and ^13^C NMR data see Tables S4, S5 and Figures S13, S14 in the Supplementary Materials. MS-ESI *m*/*z*: [M − H]^−^ calcd for C_28_H_23_O_16_ 615.1; found 615.1 (Figure 5).

*((2*R*,3*S*,4*S*,5*R*,6*S*)-6-((2-(3,4-Dihydroxyphenyl)-5,7-dihydroxy-4-oxo-4*H*-chromen-3-yl)oxy)-3,4,5-trihydroxytetrahydro-2*H*-pyran-2-yl)methyl 3-(4-hydroxyphenyl)propanoate* (**9**, IQ 4-OHPh propanoate): Yellow solid (yield 71%, 54 mg, 0.09 mmol). For ^1^H and ^13^C NMR data see Tables S4, S6 and Figures S15, S16 in the Supplementary Materials. MS-ESI *m*/*z*: [M − H]^−^ calcd for C_30_H_27_O_14_ 611.1; found 611.1 (Figure 5).

*((2*R*,3*S*,4*S*,5*R*,6*S*)-6-((2-(3,4-Dihydroxyphenyl)-5,7-dihydroxy-4-oxo-4*H*-chromen-3-yl)oxy)-3,4,5-trihydroxytetrahydro-2*H*-pyran-2-yl)methyl 3-(3,4-dihydroxyphenyl)propanoate* (**10**, IQ 3,4-diOHPh propanoate): Yellow solid (yield 64%, 60 mg, 0.10 mmol). For ^1^H and ^13^C NMR data see Tables S4, S6 and Figures S17, S18 in the Supplementary Materials. MS-ESI *m*/*z*: [M − H]^−^ calcd for C_30_H_27_O_15_ 627.1; found 627.2 (Figure 5).

*((2*R*,3*S*,4*S*,5*R*,6*S*)-6-((2-(3,4-Dihydroxyphenyl)-5,7-dihydroxy-4-oxo-4*H*-chromen-3-yl)oxy)-3,4,5-trihydroxytetrahydro-2*H*-pyran-2-yl)methyl 3-(4-hydroxy-3-methoxyphenyl)propanoate* (**11**, IQ 4-OH-3-OMePh propanoate): Yellow solid (yield 80%, 60 mg, 0.09 mmol). For ^1^H and ^13^C NMR data see Table S4, S6 and Figures S19, S20 in the Supplementary Materials. MS-ESI *m*/*z*: [M − H]^−^ calcd for C_31_H_29_O_15_ 641.2; found 641.0 (Figure 5).

### 3.5. Antioxidant Activity Measurement

Reducing capacity using Folin–Ciocalteau reagent [36], antiradical activity by DPPH [37] and ABTS^•^ [38] radical scavenging assays, and anti-lipoperoxidant activity as inhibition of lipid peroxidation of rat liver microsomes induced by *tert*-butylhydroperoxide [39] were determined as described previously with modifications specified in detail in our previous work [32].

### 3.6. Statistical Analysis

All data were analyzed with one-way ANOVA, Scheffé and Least Square Difference tests for post hoc comparisons among pairs of means using the statistical package Statext ver. 2.1 (Statext LLC, Wayne, NJ, USA). Differences were considered statistically significant when *p* < 0.05.

## 4. Conclusions

The chemoenzymatic and chemical approach have been used for the esterification at C-6″ of IQ with acyls containing an aromatic ring. First, the lipase catalyzed preparation of the derivatives was accomplished with Novozym 435 and the vinyl esters of benzoic, phenyl acetic, phenylpropanoic and cinnamic acids, but “hydroxyaromatic” acids or their vinyl esters were not substrates for the lipase. The reaction of protected “hydroxyaromatic” acids with selectively protected IQ lead to the fully protected C-6″ esterified products. The deprotection of the benzyl groups lead to the parallel saturation of the double bond yielding respective hydroxyphenylpropanoic derivatives. Several deprotection methods were attempted to overcome this problem but they proved unsuccessful. We have therefore obtained a panel of following IQ derivatives: IQ benzoate (**2**), phenylacetate (**3**), phenylpropanoate (**4**), cinnamate (**5**), 4-OH benzoate (**6**), vanillate (**7**), gallate (**8**), 4-OHPh propanoate (**9**), 3,4-diOHPh propanoate (**10**), and 4-OH-3-OMePh propanoate (**11**). The FCR reducing and DPPH scavenging capacity of most derivatives was lower or similar to IQ. In contrast, they mostly showed significantly better ABTS scavenging activity—the compounds **2**, **7**, **9**, **10** and **11** displaying the strongest activity. Almost all derivatives were much more efficient inhibitors of microsomal lipid peroxidation. Compounds **8** and **3** (IC_50_ ≈ 7 µM) were the most active, but strong activity was noted also for phenylpropanoates **9**–**11** (IC_50_ ≈ 9.4 µM), which proved to be the most active compounds from the whole series.

## Figures and Tables

**Table 1:** Radical scavenging and anti-lipoperoxidant activity of isoquercitrin and its conjugates **2**–**11**.

Compounds	FCR (GAE)	DPPH (IC_50_, µM)	ABTS (TE)	Lpx (IC_50_, µM)
Isoquercitrin (**1**, IQ)	1.11 ± 0.30 ^a^	1.40 ± 0.06 ^d^	1.98 ± 0.07 ^f^	972 ± 11
IQ benzoate (**2**)	1.24 ± 0.05 ^a^	2.85 ± 0.22	7.34 ± 0.01 ^g^	8.29 ± 0.39 ^h^
IQ phenylacetate (**3**)	1.45 ± 0.03 ^b^	1.89 ± 0.04 ^d^	1.10 ± 0.20	6.68 ± 0.39 ^i^
IQ phenylpropanoate (**4**)	1.47 ± 0.04 ^b^	3.31 ± 0.22	3.65 ± 0.18	10.5 ± 0.7
IQ cinnamate (**5**)	0.51 ± 0.13 ^c^	4.64 ± 0.16	0.64 ± 0.05	295 ± 14
IQ 4-OH benzoate (**6**)	0.90 ± 0.05 ^a^	11.5 ± 0.4	1.81 ± 0.25 ^f^	12.6 ^o^ ± 0.3 ^j^
IQ vanillate (**7**)	1.50 ± 0.04 ^b^	2.18 ± 0.04 ^e^	7.05 ± 0.07 ^g^	14.1 ± 0.6 ^j^
IQ gallate (**8**)	0.52 ± 0.15 ^c^	2.28 ± 0.18 ^e^	5.27 ± 0.20	6.64 ± 0.27 ^i^
IQ 4-OHPh propanoate (**9**)	0.59 ± 0.06 ^c^	1.77 ± 0.06 ^d^	7.18 ± 0.07 ^g^	9.39 ± 0.29 ^h^
IQ 3,4-diOHPh propanoate (**10**)	0.88 ± 0.04 ^a^	1.67 ± 0.09 ^d^	7.20 ± 0.15 ^g^	9.41 ± 0.28 ^h^
IQ 4-OH-3-OMePh propanoate (**11**)	1.48 ± 0.03 ^b^	2.22 ± 0.11 ^e^	7.33 ± 0.05 ^g^	9.36 ± 0.22 ^h^

Data are presented as means ± SE from at least three independent experiments performed in triplicate. ^a-j^ The values marked with the same letter are not significantly different. FCR: Folin-Ciocalteau reduction; GAE: gallic acid equivalents; DPPH: 1,1-diphenyl-2-picrylhydrazyl radical scavenging; ABTS: 2,2′-azinobis-(3-ethylbenzothiazoline-6-sulfonic acid) radical cation scavenging; TE: trolox equivalents; Lpx: lipid peroxidation; IC_50_: the concentration of the tested compound that inhibited the reaction by 50%.

## Supplementary Materials

Supplementary materials containing ^1^H, ^13^C NMR data of the new compounds can be found at www.mdpi.com/1422-0067/18/5/1074/s1.

## Abbreviations

ABTS	2,2′-Azinobis-(3-ethylbenzothiazoline-6-sulfonic acid)
BnBr	Benzyl bromide
CAL-B	Lipase B from *Candida antarctica*
COSY	Correlation spectroscopy
DCC	*N*,*N*′-Dicyclohexylcarbodiimide
DCM	Dichloromethane
DMAP	4-Dimethylaminopyridine
DMF	Dimethylformamide
DMSO	Dimethyl sulfoxide
DPPH	1,1-Diphenyl-2-picrylhydrazyl
eq.	Equivalents
FCR	Folin-ciocalteau reduction
FDA	Food & drug administration
GAE	Gallic acid equivalents
GRAS	Generally recognized as safe
HMBC	Heteronuclear multiple-bond correlation spectroscopy
HSQC	Heteronuclear single-quantum correlation spectroscopy
IC_50_	The concentration of the tested compound that inhibited the reaction by 50%
IQ	Isoquercitrin (quercetin-3-*O*-β-d-glucopyranoside)
Lpx	Lipid peroxidation
MS	Mass spectrometry
MS-ESI	Electron spray ionization mass spectrometry
NMR	Nuclear magnetic resonance
RT	Room temperature
TBARS	Thiobarbituric acid reactive substances
TBDMSCl	*tert*-Butyldimethylsilyl chloride
TE	Trolox-equivalents
THF	Tetrahydrofuran
TOCSY	Two-dimensional nuclear magnetic resonance spectroscopy
UV	Ultra violet
