# Supplementary material for: Synthesis and Antiradical Activity of Isoquercitrin Esters with Aromatic Acids and Their Homologues
Source: Int J Mol Sci. 2017 May 17;18(5):1074. doi: 10.3390/ijms18051074 (PMC5454983; doi:10.3390/ijms18051074)
Supplement: Supplementary file 1 [file ijms-18-01074-s001.pdf]

International Journal of Molecular Sciences

Supplementary Materials

Synthesis and Antiradical Activity of Isoquercitrin Esters with Aromatic Acids and Their Homologues

Eva Heřmánková-Vavříková, Alena Křenková, Lucie Petrásková, Christopher Steven Chambers, Jakub Zápal, Marek Kuzma, Kateřina Valentová*, Vladimír Křen

Laboratory of Biotransformation, Institute of Microbiology, Czech Academy of Sciences, Vídeňská 1083, CZ-142 20 Prague, Czech Republic

*Corresponding author Tel.: +420-296-442-509; E-mail: kata.valentova@email.cz

Content **Page**

Table S1. ^{13}C NMR data of the products **2** - **5** 3

Table S2. ^{1}H NMR data of the products **2** - **3** 4

Table S3. ^{1}H NMR data of prepared compounds **4** - **5** 5

Table S4. ^{13}C NMR data of prepared compounds **6** - **11** 6

Table S5. ^{1}H NMR data of prepared compounds **6** - **8** 7

Table S6. ^{1}H NMR data of prepared compounds **9** - **11** 8

Figure S1. ^{13}C NMR spectrum of compound **2** (DMSO-d_6, 303.2 K). 9

Figure S2. ^{1}H NMR spectrum of compound **2** (DMSO-d_6, 303.2 K). 10

Figure S3. ^{13}C NMR spectrum of compound **3** (DMSO-d_6, 303.2 K). 11

Figure S4. ^{1}H NMR spectrum of compound **3** (DMSO-d_6, 303.2 K). 12

Figure S5. ^{13}C NMR spectrum of compound **4** (DMSO-d_6, 303.2 K). 13

Figure S6. ^{1}H NMR spectrum of compound **4** (DMSO-d_6, 303.2 K). 14

Figure S7. ^{13}C NMR spectrum of compound **5** (DMSO-d_6, 303.2 K). 15

Figure S8. ^{1}H NMR spectrum of compound **5** (DMSO-d_6, 303.2 K). 16

Figure S9. ^{13}C NMR spectrum of compound **6** (DMSO-d_6, 303.2 K). 17

Figure S10. ^{1}H NMR spectrum of compound **6** (DMSO-d_6, 303.2 K). 18

Figure S11. ^{13}C NMR spectrum of compound **7** (DMSO-d_6, 303.2 K). 19

Figure S12. ^{1}H NMR spectrum of compound **7** (DMSO-d_6, 303.2 K). 20

Figure S13. ^{13}C NMR spectrum of compound **8** (DMSO-d_6, 303.2 K). 21

Figure S14. ^{1}H NMR spectrum of compound **8** (DMSO-d_6, 303.2 K). 22

Figure S15. ^{13}C NMR spectrum of compound **9** (DMSO-d_6, 303.2 K). 23

Figure S16. ^{1}H NMR spectrum of compound **9** (DMSO-d_6, 303.2 K). 24
Figure S17. ^{13}C NMR spectrum of compound **10** (DMSO-d_6, 303.2 K). 25
Figure S18. ^{1}H NMR spectrum of compound **10** (DMSO-d_6, 303.2 K). 26
Figure S19. ^{13}C NMR spectrum of compound **11** (DMSO-d_6, 303.2 K). 27
Figure S20. ^{1}H NMR spectrum of compound **11** (DMSO-d_6, 303.2 K). 28
Figure S21. Structures of the intermediates **32** - **40** 29
Table S7. ^{13}C NMR data of the intermediates **32** - **34** 30
Table S8. ^{1}H NMR data of the intermediates **32** - **34** 32
Table S9. ^{13}C NMR data of the intermediates **35** - **38** 34
Table S10. ^{1}H NMR data of the intermediates **35** - **36** 36
Table S11. ^{1}H NMR data of the intermediates **37** - **38** 38
Table S12. ^{13}C NMR data of the intermediates **39** - **40** 40
Table S13. ^{1}H NMR data of the intermediates **39** - **40** 42
Figure S22. ^{13}C NMR spectrum of compound **32** (DMSO-d_6, 303.2 K). 44
Figure S23. ^{1}H NMR spectrum of compound **32** (DMSO-d_6, 303.2 K). 45
Figure S24. ^{13}C NMR spectrum of compound **33** (acetone, 293.2 K). 46
Figure S25. ^{1}H NMR spectrum of compound **33** (acetone, 293.2 K). 47
Figure S26. ^{13}C NMR spectrum of compound **34** (DMSO-d_6, 303.2 K). 48
Figure S27. ^{1}H NMR spectrum of compound **34** (DMSO-d_6, 303.2 K). 49
Figure S28. ^{13}C NMR spectrum of compound **35** (DMSO-d_6, 303.2 K). 50
Figure S29. ^{1}H NMR spectrum of compound **35** (DMSO-d_6, 303.2 K). 51
Figure S30. ^{13}C NMR spectrum of compound **36** (DMSO-d_6, 303.2 K). 52
Figure S31. ^{1}H NMR spectrum of compound **36** (DMSO-d_6, 303.2 K). 53
Figure S32. ^{13}C NMR spectrum of compound **37** (DMSO-d_6, 303.2 K). 54
Figure S33. ^{1}H NMR spectrum of compound **37** (DMSO-d_6, 303.2 K). 55
Figure S34. ^{13}C NMR spectrum of compound **38** (DMSO-d_6, 303.2 K). 56
Figure S35. ^{1}H NMR spectrum of compound **38** (DMSO-d_6, 303.2 K). 57
Figure S36. ^{13}C NMR spectrum of compound **39** (DMSO-d_6, 303.2 K). 58
Figure S37. ^{1}H NMR spectrum of compound **39** (DMSO-d_6, 303.2 K). 59
Figure S38. ^{13}C NMR spectrum of compound **40** (DMSO-d_6, 303.2 K). 60
Figure S39. ^{1}H NMR spectrum of compound **40** (DMSO-d_6, 303.2 K). 61

Table S1. ^{13}C NMR data of the products **2** - **5** (DMSO-d_6, 303.2 K).

**Comp.**	**2**		**3**		**4**		**5**	
**Atom number**	**δ**	**m**	**δ**	**m**	**δ**	**m**	**δ**	**m**
**2**	156.38	s	156.51	s	156.38	s	156.33	s
**3**	132.94	s	133.17	s	133.10	s	133.11	s
**4**	177.40	s	177.43	s	177.40	s	177.42	s
**4a**	103.86	s	103.96	s	103.85	s	103.86	s
**5**	161.29	s	161.33	s	161.23	s	161.19	s
**6**	98.79	d	98.76	d	98.74	d	98.74	d
**7**	164.17	s	164.24	s	164.23	s	164.12	s
**8**	93.58	d	93.62	d	93.52	d	93.50	d
**8a**	156.30	s	156.41	s	156.32	s	156.30	s
**1′**	121.13	s	121.14	s	121.10	s	121.12	s
**2′**	116.12	d	116.26	d	116.18	d	116.24	d
**3′**	144.90	s	144.91	s	144.88	s	144.85	s
**4′**	148.51	s	148.60	s	148.56	s	148.53	s
**5′**	115.20	d	115.23	d	115.18	d	115.22	d
**6′**	121.47	d	121.57	d	121.51	d	121.52	d
**1″**	100.63	d	100.94	d	100.81	d	100.77	d
**2″**	74.02	d	73.98	d	73.97	d	74.02	d
**3″**	76.41	d	76.31	d	76.33	d	76.37	d
**4″**	70.08	d	69.87	d	70.03	d	70.04	d
**5″**	74.25	d	74.13	d	74.17	d	74.30	d
**6″**	63.74	t	63.45	t	63.18	t	63.37	t
**1‴**	165.37	s	170.73	s	171.64	s	165.78	s
**2‴**	-	-	39.95	t	34.91	t	117.58	d
**3‴**	-	-	-	-	30.11	t	144.35	d
ipso	129.56	s	134.15	s	140.30	s	133.85	s
ortho	128.82	d	129.23	d	128.05	d	128.21	d
meta	128.48	d	128.21	d	128.21	d	128.88	d
para	133.07	d	126.73	d	125.94	d	130.40	d

m - the multiplicity of ^{13}C NMR signals was resolved using multiplicity-edited gradient-enhanced ^{1}H-^{13}C HSQC

Table S2. ^{1}H NMR data of the products **2** - **3** (DMSO-d_6, 303.2 K).

Comp.	2				3			
Atom number	δ	n	m	*J* [Hz]	δ	n	m	*J* [Hz]
**2**	-	-	-	-	-	-	-	-
**3**	-	-	-	-	-	-	-	-
**4**	-	-	-	-	-	-	-	-
**4a**	-	-	-	-	-	-	-	-
**5**	-	-	-	-	-	-	-	-
**6**	6.185	1	d	2.1	6.207	1	d	2.1
**7**	-	-	-	-	-			
**8**	6.345	1	d	2.1	6.424	1	d	2.1
**8a**	-	-	-	-	-	-	-	-
**1′**	-	-	-	-	-	-	-	-
**2′**	7.530	1	d	2.2	7.583	1	d	2.2
**3′**	-	-	-	-	-	-	-	-
**4′**	-	-	-	-	-	-	-	-
**5′**	6.787	1	d	8.0	6.860	1	d	8.3
**6′**	7.514	1	dd	8.0, 2.2	7.562	1	dd	8.3, 2.2
**1″**	5.552	1	d	7.5	5.440	1	d	7.5
**2″**	3.34 ^{H}	1	m	-	3.300	1	dd	7.5, 8.6
**3″**	3.30 ^{H}	1	m	-	3.255	1	dd	8.6, 8.6
**4″**	3.26 ^{H}	1	m	-	3.153	1	dd	9.6, 8.6
**5″**	3.458	1	ddd	9.2, 6.7, 2.3	3.327	1	ddd	9.6, 6.4, 2.1
**6″**	4.399	1	dd	11.8, 2.3	4.197	1	dd	11.8, 2.1
	4.210	1	dd	11.8, 6.7	3.966	1	dd	11.8, 6.4
**5-OH**	12.613	1	s	-	12.645	1	s	-
**7-OH**	10.770	1	br.s.	-	10.835	1	s	-
**3′-OH**	9.267	1	br.s.	-	9.206	1	s	-
**4′-OH**	9.612	1	br.s.	-	9.705	1	s	-
**2″-OH**	5.387	1	br.s.	-	n.a.	-	-	-
**3″-OH**	5.233	1	br.s.	-	n.a.	-	-	-
**4″-OH**	5.198	1	br.s.	-	n.a.	-	-	-
**1‴**	-	-	-	-	-	-	-	-
**2‴**	-	-	-	-	3.402	1	d	15.5
	-	-	-	-	3.350	1	d	15.5
**3‴**	-	-	-	-	-	-	-	-
ipso	-	-	-	-	-	-	-	-
ortho	7.687	2	m	-	7.03 ^{H}	2	m	-
meta	7.338	2	m	-	7.20 ^{H}	2	m	-
para	7.551	1	m	-	7.19 ^{H}	1	m	-

^{H} - HSQC readout; n.a. – not asssigned due to signal broadening of the hydroxyl signals

Table S3. ^{1}H NMR data of prepared compounds **4** - **5** (DMSO-d_6, 303.2 K).

	**4**				**5**			
**Atom number**	**δ**	**n**	**m**	***J* [Hz]**	**δ**	**n**	**m**	***J* [Hz]**
**2**	-	-	-	-	-	-	-	-
**3**	-	-	-	-	-	-	-	-
**4**	-	-	-	-	-	-	-	-
**4a**	-	-	-	-	-	-	-	-
**5**	-	-	-	-	-	-	-	-
**6**	6.154	1	d	2.1	6.107	1	d	2.0
**7**	-	-	-	-	-	-	-	-
**8**	6.342	1	d	2.1	6.331	1	d	2.0
**8a**	-	-	-	-	-	-	-	-
**1′**	-	-	-	-	-	-	-	-
**2′**	7.544	1	d	2.3	7.551	1	d	2.2
**3′**	-	-	-	-	-	-	-	-
**4′**	-	-	-	-	-	-	-	-
**5′**	6.838	1	d	8.0	6.830	1	d	8.4
**6′**	7.530	1	dd	8.0, 2.3	7.526	1	dd	8.4, 2.2
**1″**	5.453	1	d	7.5	5.496	1	d	7.4
**2″**	3.29 [H]	1	m	-	3.310	1	m	-
**3″**	3.250	1	t	8.9	3.281	1	m	-
**4″**	3.145	1	t	8.9	3.213	1	dd	9.5, 8.9
**5″**	3.31 [H]	1	m	-	3.405	1	ddd	9.5, 6.7, 2.2
**6″**	4.184	1	dd	11.8, 2.1	4.304	1	dd	11.8, 2.2
	3.919	1	dd	11.8, 6.8	4.090	1	dd	11.8, 6.7
**5-OH**	12.611	1	s	-	12.608	1	s	-
**7-OH**	10.781	1	br.s.	-	10.775	1	s	-
**3′-OH**	9.221	1	br.s.	-	9.207	1	s	-
**4′-OH**	9.674	1	br.s.	-	9.659	1	s	-
**2″-OH**	5.354	1	br.s.	-	n.a.	-	-	-
**3″-OH**	5.156	1	br.s.	-	n.a.	-	-	-
**4″-OH**	5.156	1	br.s.	-	n.a.	-	-	-
**1‴**	-	-	-	-	-	-	-	-
**2‴**	2.340	2	m	-	6.343	1	d	16.0
**3‴**	2.575	2	m	-	7.413	1	d	16.0
ipso	-	-	-	-	-	-	-	-
ortho	7.017	2	m	-	7.509	2	m	-
meta	7.189	2	m	-	7.416	2	m	-
para	7.131	1	m	-	7.419	1	m	-

[H] - HSQC readout; n.a. – not asssigned due to signal broadening of the hydroxyl signals

Table S4. ^{13}C NMR data of prepared compounds **6** - **11** (DMSO-d_6, 303.2 K).

	**6**		**7**		**8**		**9**		**10**		**11**	
**Atom number**	**δ**	**m**	**δ**	**m**	**δ**	**m**	**δ**	**m**	**δ**	**m**	**δ**	**m**
**2**	156.22	s	156.17	s	156.47	s	156.43	s	156.39	s	156.39	s
**3**	132.96	s	132.99	s	133.42	s	133.11	s	133.15	s	133.12	s
**4**	177.37	s	177.27	s	177.31	s	177.42	s	177.37	s	177.38	s
**4a**	103.76	s	103.61	s	103.94	s	103.88	s	103.80	s	103.83	s
**5**	161.26	s	161.21	s	161.22	s	161.25	s	161.24	s	161.23	s
**6**	98.85	d	98.92	d	98.74	d	98.73	d	98.82	d	98.73	d
**7**	164.38	s	164.75	s	164.21	s	164.18	s	164.43	s	164.29	s
**8**	93.54	d	93.57	d	93.56	d	93.53	d	93.59	d	93.52	d
**8a**	156.31	s	156.32	s	156.34	s	156.34	s	156.37	s	156.34	s
**1′**	121.06	s	121.03	s	120.96	s	121.11	s	121.09	s	121.08	s
**2′**	116.04	d	115.98	d	115.81	d	116.19	d	116.16	d	116.17	d
**3′**	144.91	s	144.92	s	144.84	s	144.88	s	144.88	s	144.88	s
**4′**	148.53	s	148.59	s	148.54	s	148.54	s	148.57	s	148.56	s
**5′**	115.19	d	115.18	d	115.32	d	115.18	d	115.17	d	115.16	d
**6′**	121.49	d	121.52	d	121.89	d	121.51	d	121.51	d	121.51	d
**1″**	100.65	d	100.79	d	101.44	d	100.80	d	100.92	d	100.88	d
**2″**	74.01	d	74.03	d	74.09	d	73.97	d	73.99	d	73.98	d
**3″**	76.43	d	76.42	d	76.35	d	76.34	d	76.34	d	76.33	d
**4″**	69.98	d	70.08	d	69.56	d	70.02	d	69.99	d	69.97	d
**5″**	74.43	d	74.44	d	74.31	d	74.19	d	74.18	d	74.20	d
**6″**	63.29	t	63.50	t	63.22	t	63.07	t	63.03	t	63.05	t
**1‴**	165.24	s	165.28	s	165.72	s	171.75	s	171.83	s	171.80	s
**2‴**	-	-	120.40	s	-	-	35.36	t	35.24	t	35.36	t
**3‴**	-	-	123.12	d	-	-	29.34	t	29.50	t	29.79	t
**4‴**	-	-	115.10	d	-	-	-	-	131.23	s	131.18	s
**5‴**	-	-	151.44	s	-	-	-	-	118.66	d	120.17	d
**6‴**	-	-	147.27	s	-	-	-	-	115.45	d	115.29	d
**7‴**	-	-	112.34	d	-	-	-	-	143.42	s	144.68	s
**8‴**	-	-	-	-	-	-	-	-	144.98	s	147.35	s
**9‴**	-	-	-	-	-	-	-	-	115.58	d	112.29	d
ipso	120.20	s	-	-	119.37	s	130.40	s	-	-	-	-
ortho	131.11	d	-	-	108.63	d	128.93	d	-	-	-	-
meta	115.19	d	-	-	145.48	s	115.05	d	-	-	-	-
para	161.88	s	-	-	138.44	s	155.50	s	-	-	-	-
6‴-MeO	-	-	55.45	q	-	-	-	-	-	-	-	-
8‴-MeO	-	-	-	-	-	-	-	-	-	-	55.51	q

m - the multiplicity of ^{13}C NMR signals was resolved using multiplicity-edited gradient-enhanced ^{1}H-^{13}C HSQC

Table S5. ^{1}H NMR data of prepared compounds **6** - **8** (DMSO-d_6, 303.2 K).

Comp.	6				7				8			
Atom number	δ	n	m	*J* [Hz]	δ	n	m	*J* [Hz]	δ	n	m	*J* [Hz]
2	-	-	-	-	-	-	-	-	-	-	-	-
3	-	-	-	-	-	-	-	-	-	-	-	-
4	-	-	-	-	-	-	-	-	-	-	-	-
4a	-	-	-	-	-	-	-	-	-	-	-	-
5	-	-	-	-	-	-	-	-	-	-	-	-
6	6.198	1	d	2.1	6.163	1	d	2.0	6.176	1	d	2.1
7	-	-	-	-	-	-	-	-	-	-	-	-
8	6.355	1	d	2.1	6.327	1	d	2.0	6.360	1	d	2.1
8a	-	-	-	-	-	-	-	-	-	-	-	-
1′	-	-	-	-	-	-	-	-	-	-	-	-
2′	7.518	1	d	2.2	7.504	1	d	2.2	7.436	1	d	2.2
3′	-	-	-	-	-	-	-	-	-	-	-	-
4′	-	-	-	-	-	-	-	-	-	-	-	-
6′	7.518	1	d	2.2	7.504	1	d	2.2	7.436	1	d	2.2
1′	-	-	-	-	-	-	-	-	-	-	-	-
5′	6.789	1	d	8.2	6.766	1	d	8.4	6.724	1	d	8.4
6′	7.528	1	dd	8.2, 2.2	7.527	1	dd	8.4, 2.2	7.570	1	dd	8.4, 2.2
1″	5.558	1	d	7.6	5.535	1	d	7.6	5.434	1	d	7.5
2″	3.331	1	dd	8.8, 7.6	3.318	1	dd	9.0, 7.6	3.288	1	m	-
3″	3.288	1	dd	8.8, 8.6	3.287	1	dd	9.0, 8.5	3.288	1	m	-
4″	3.237	1	dd	9.6, 8.6	3.232	1	dd	9.6, 8.5	3.360	1	dd	9.7, 8.3
5″	3.419	1	ddd	9.6, 6.6, 2.2	3.438	1	ddd	9.6, 6.7, 2.2	3.428	1	ddd	9.7, 4.5, 2.0
6″	4.358	1	dd	11.8, 2.2	4.386	1	dd	11.8, 2.2	4.257	1	dd	11.9, 2.0
	4.094	1	dd	11.8, 6.6	4.117	1	dd	11.8, 6.7	4.169	1	dd	11.9, 4.5
5-OH	12.626			-	12.582	1	s	-	12.540	1	s	-
7-OH	9.949	3	br.s	-	n.a.	-	-	-	n.a.	-	-	-
4′-OH									n.a.	-	-	-
3′-OH									n.a.	-	-	-
2″-OH	5.189	2	br.s	-					n.a.	-	-	-
3″-OH				-					n.a.	-	-	-
4″-OH	5.351	1	br.s	-					n.a.	-	-	-
3‴	-	-	-	-	7.179	1	dd	8.2, 2.0	-	-	-	-
4‴	-	-	-	-	6.690	1	d	8.2	-	-	-	-
7‴	-	-	-	-	7.262	1	d	2.0	-	-	-	-
ortho	7.559	2	m	-	-	-	-	-	6.889	2	s	-
meta	6.699	2	m	-	-	-	-	-	-	-	-	-
5‴-OH	-	-	-	-	n.a.	-	-	-	-	-	-	-
6‴-MeO	-	-	-	-	3.696	3	s	-	-	-	-	-
meta-OH	-	-	-	-	-	-	-	-	n.a.	-	-	-
para-OH	-	-	-	-	-	-	-	-	n.a.	-	-	-

n.a. - not assigned due to the broadening of the hydroxyl signals

Table S6. ^{1}H NMR data of prepared compounds **9** - **11** (DMSO-d_6, 303.2 K).

Comp.	9				10				11			
**Atom number**	**δ**	**n**	**m**	***J* [Hz]**	**δ**	**n**	**m**	***J* [Hz]**	**δ H**	**n**	**m**	***J* [Hz]**
**2**	-	-	-	-	-	-	-	-	-	-	-	-
**3**	-	-	-	-	-	-	-	-	-	-	-	-
**4**	-	-	-	-	-	-	-	-	-	-	-	-
**4a**	-	-	-	-	-	-	-	-	-	-	-	-
**5**	-	-	-	-	-	-	-	-	-	-	-	-
**6**	6.167	1	d	2.1	6.168	1	d	2.1	6.154	1	d	2.1
**7**	-	-	-	-	-	-	-	-	-	-	-	-
**8**	6.363	1	d	2.1	6.367	1	d	2.1	6.352	1	d	2.1
**8a**	-	-	-	-	-	-	-	-	-	-	-	-
**1′**	-	-	-	-	-	-	-	-	-	-	-	-
**2′**	7.540	1	m	-	7.534	1	m	-	7.537	1	m	-
**3′**	-	-	-	-	-	-	-	-	-	-	-	-
**4′**	-	-	-	-	-	-	-	-	-	-	-	-
**5′**	6.835	1	d	8.0	6.828	1	m	-	6.831	1	m	-
**6′**	7.530	1	m	-	7.529	1	m	-	7.530	1	m	-
**1″**	5.444	1	d	7.4	5.415	1	d	7.6	5.436	1	d	7.6
**2″**	3.28 ^{H}	1	m	-	3.279	1	dd	9.0, 7.6	3.285	1	m	-
**3″**	3.26 ^{H}	1	m	-	3.242	1	dd	9.0, 8.6	3.248	1	t	8.7
**4″**	3.143	1	m	-	3.143	1	dd	9.8, 8.6	3.154	1	dd	8.7, 9.8
**5″**	3.30 ^{H}	1	m	-	3.308	1	ddd	9.8, 6.7, 2.1	3.320	1	ddd	9.8, 6.7, 2.2
**6″**	4.175	1	dd	11.8, 2.0	4.166	1	dd	11.8, 2.1	4.176	1	dd	11.8, 2.2
	3.913	1	dd	11.8, 6.8	3.924	1	dd	11.8, 6.7	3.937	1	dd	11.8, 6.7
**5-OH**	12.611	1	s	-	12.599	1	s	-	12.603	1	s	-
**7-OH**	10.807	1	s	-	n.a.	-	-	-	n.a.	-	-	-
**4'-OH**	9.678	1	s	-	n.a.	-	-	-				
**3′-OH**	9.186	1	s	-	n.a.	-	-	-				
**2″-OH**	5.349	1	d	4.4	5.339	1	br.s.	-				
**3″-OH**	5.157	1	d	5.9	5.142	2	br.s.	-				
**4″-OH**	5.143	1	d	4.8								
**2‴**	2.262	2	m	-	2.242	2	m	-	2.301	2	m	-
**3‴**	2.460	2	m	-	2.423	2	m	-	2.486	2	m	-
**4‴**	-	-	-	-	-	-	-	-	-	-	-	-
**5‴**	-	-	-	-	6.254	1	dd	8.0, 2.1	6.404	1	dd	8.0, 2.0
**6‴**	-	-	-	-	6.554	1	d	8.0	6.595	1	d	8.0
**9‴**	-	-	-	-	6.480	1	d	2.1	6.630	1	d	2.0
ipso	-	-	-	-	n.a.	-	-	-	-	-	-	-
ortho	6.807	2	m	-	n.a.	-	-	-	-	-	-	-
meta	6.591	2	m	-	-	-	-	-	-	-	-	-
5‴-OH	-	-	-	-	6.480	1	d	2.1	-	-	-	-
para-OH	9.088	1	s	-	-	-	-	-	-	-	-	-
**7‴-OH**	-	-	-	-	n.a.	-	-	-	n.a.	-	-	-
**8‴-OH**	-	-	-	-	n.a.	-	-	-	-	-	-	-
8‴-MeO	-	-	-	-	-	-	-	-	3.703	3	s	-

^{H} - HSQC readout; n.a. - not assigned due to the broadening of the hydroxyl signals

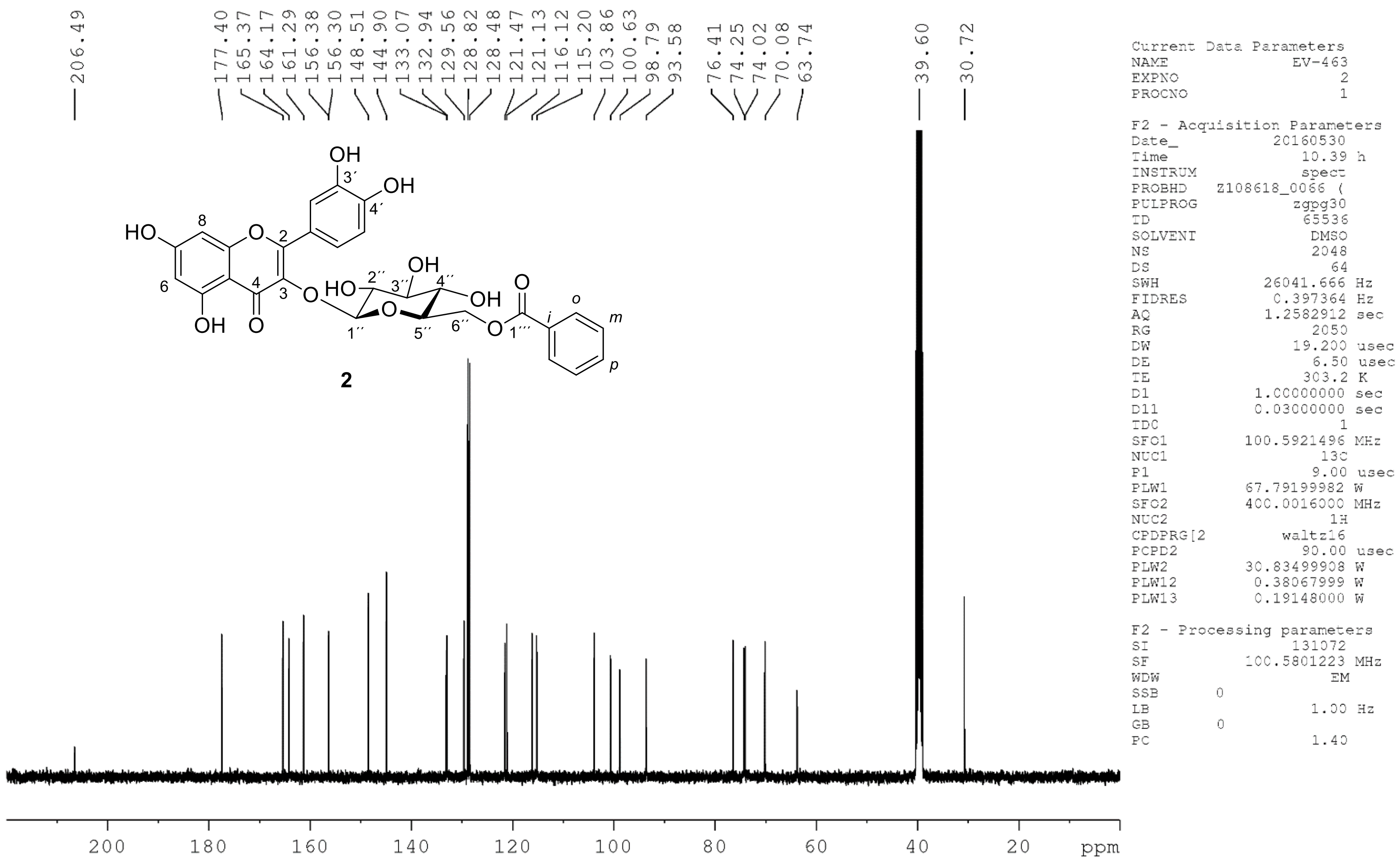

Figure S1. ^{13}C NMR spectrum of compound **2** (DMSO-d_6, 303.2 K).

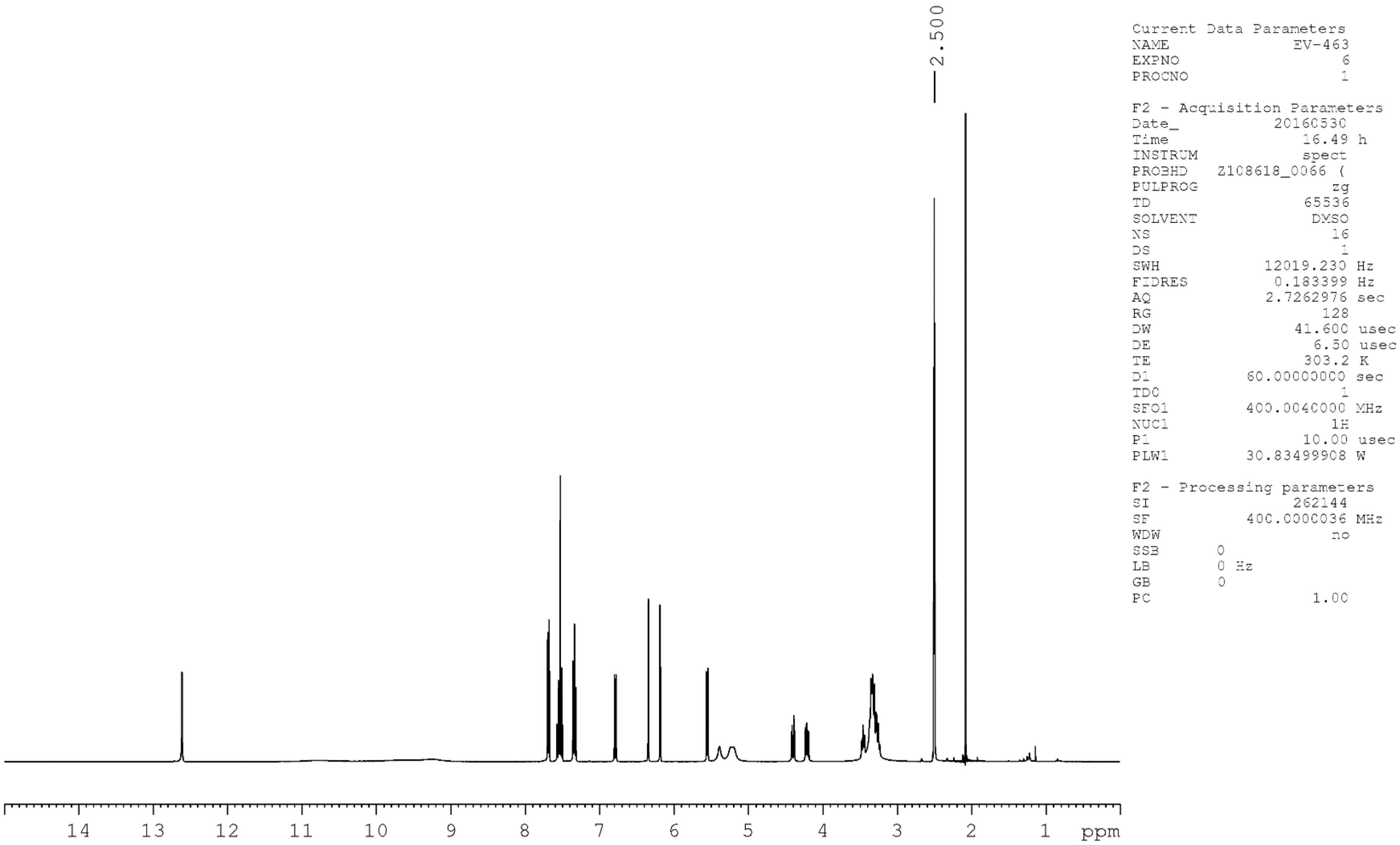

Figure S2. ^{1}H NMR spectrum of compound **2** (DMSO-d_6, 303.2 K).

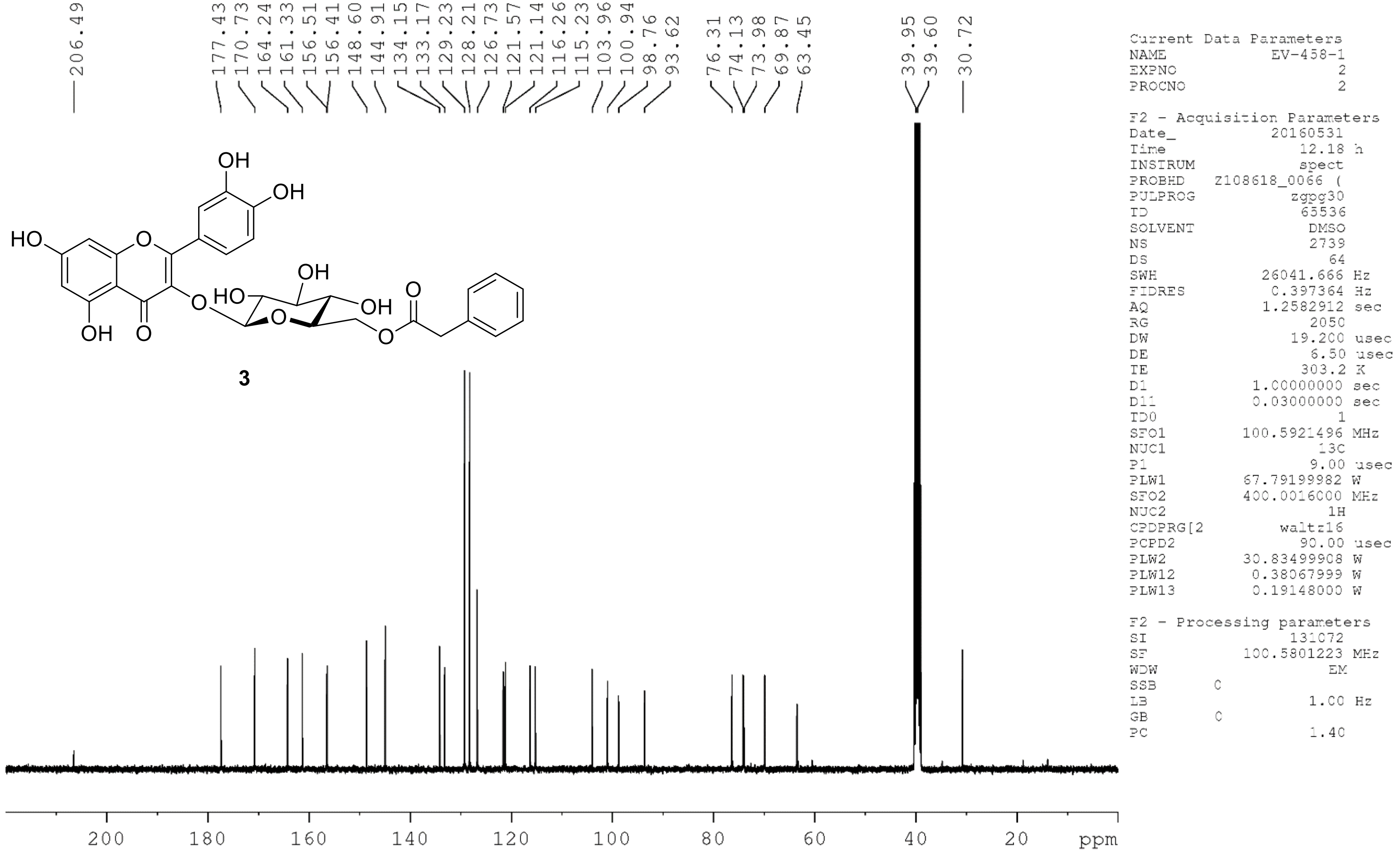

Figure S3. ^{13}C NMR spectrum of compound **3** (DMSO-d_6, 303.2 K).

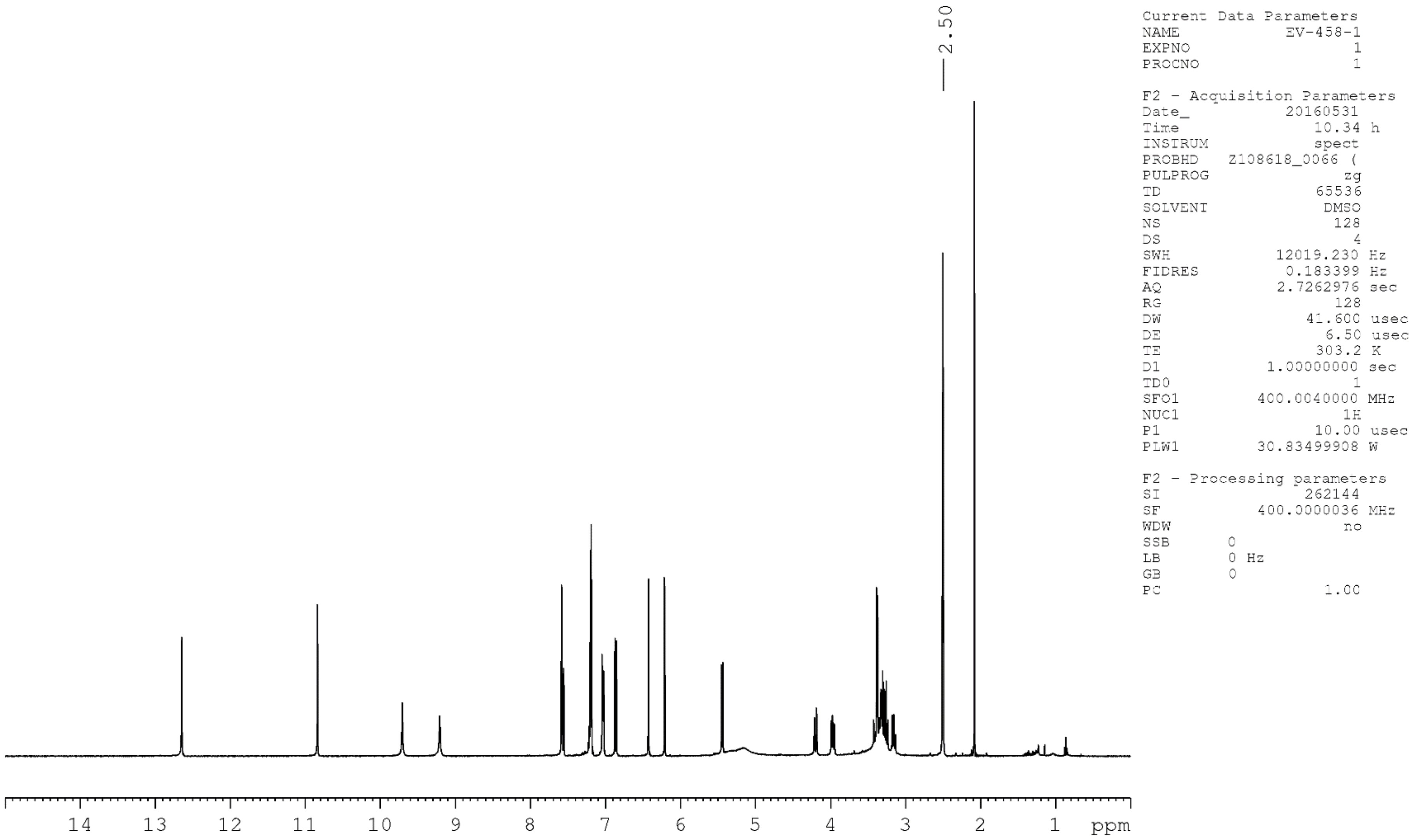

Figure S4. ^{1}H NMR spectrum of compound **3** (DMSO-d_6, 303.2 K).

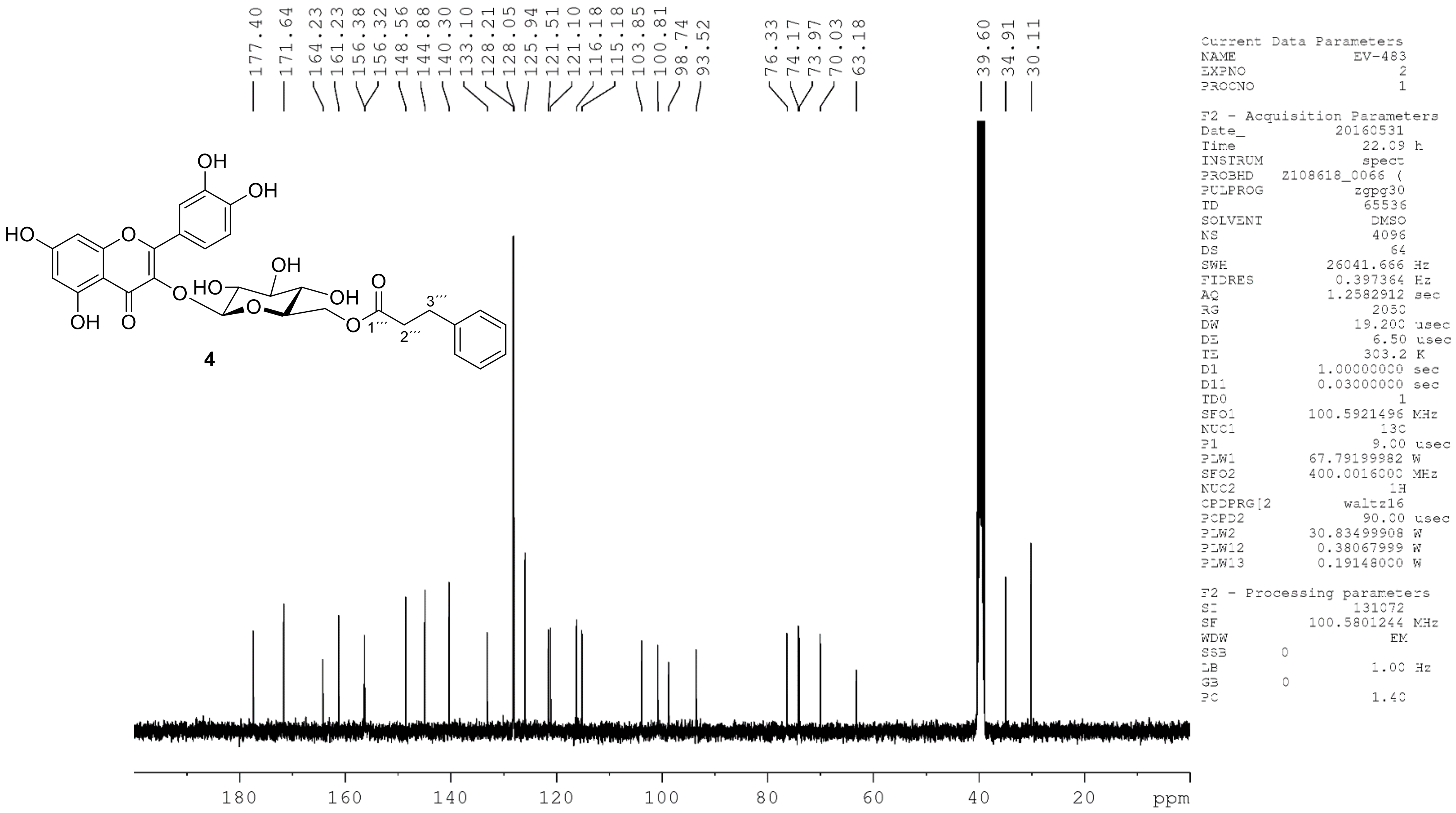

Figure S5. ^{13}C NMR spectrum of compound **4** (DMSO-d_6, 303.2 K).

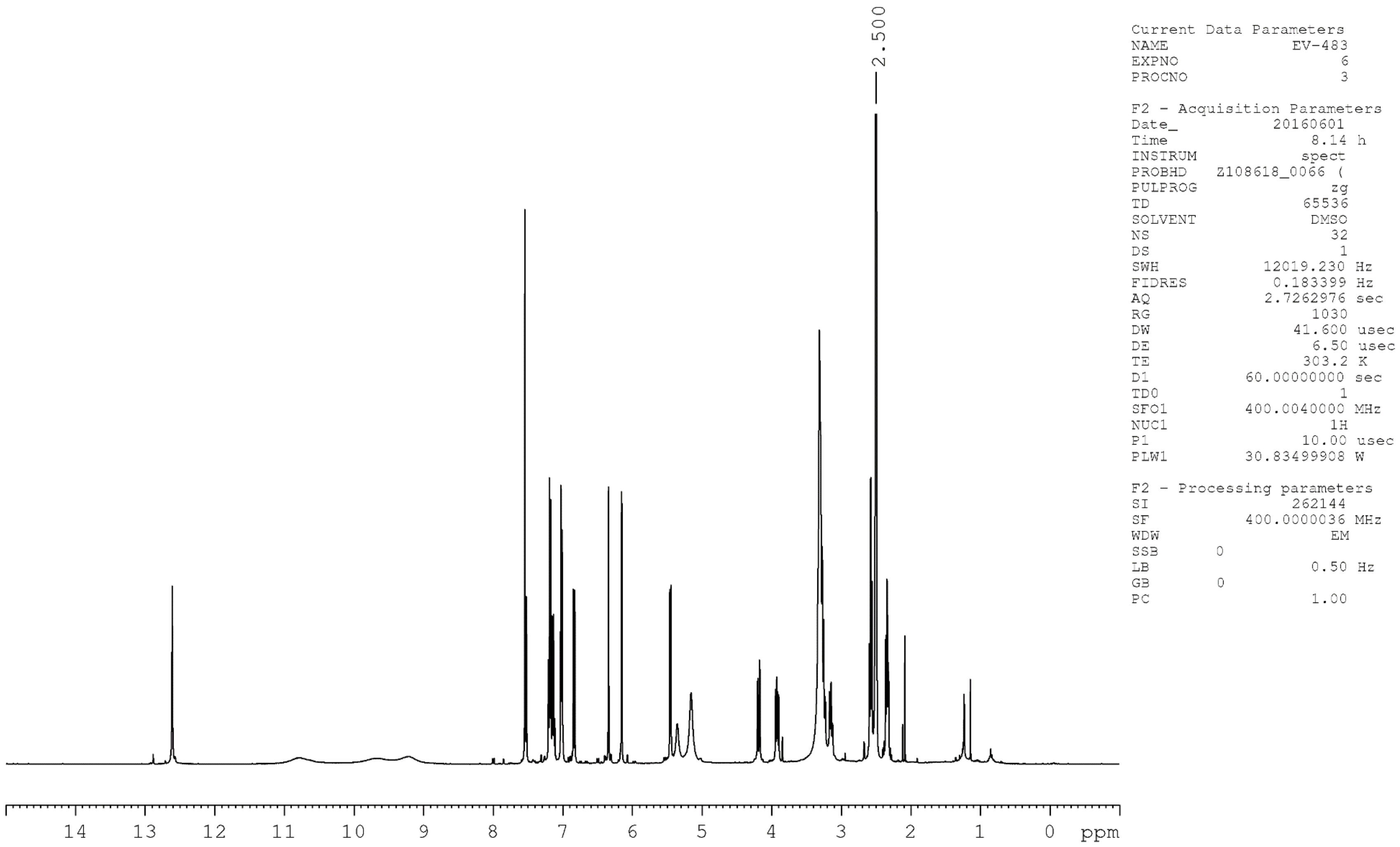

Figure S6. ^{1}H NMR spectrum of compound **4** (DMSO-d_6, 303.2 K).

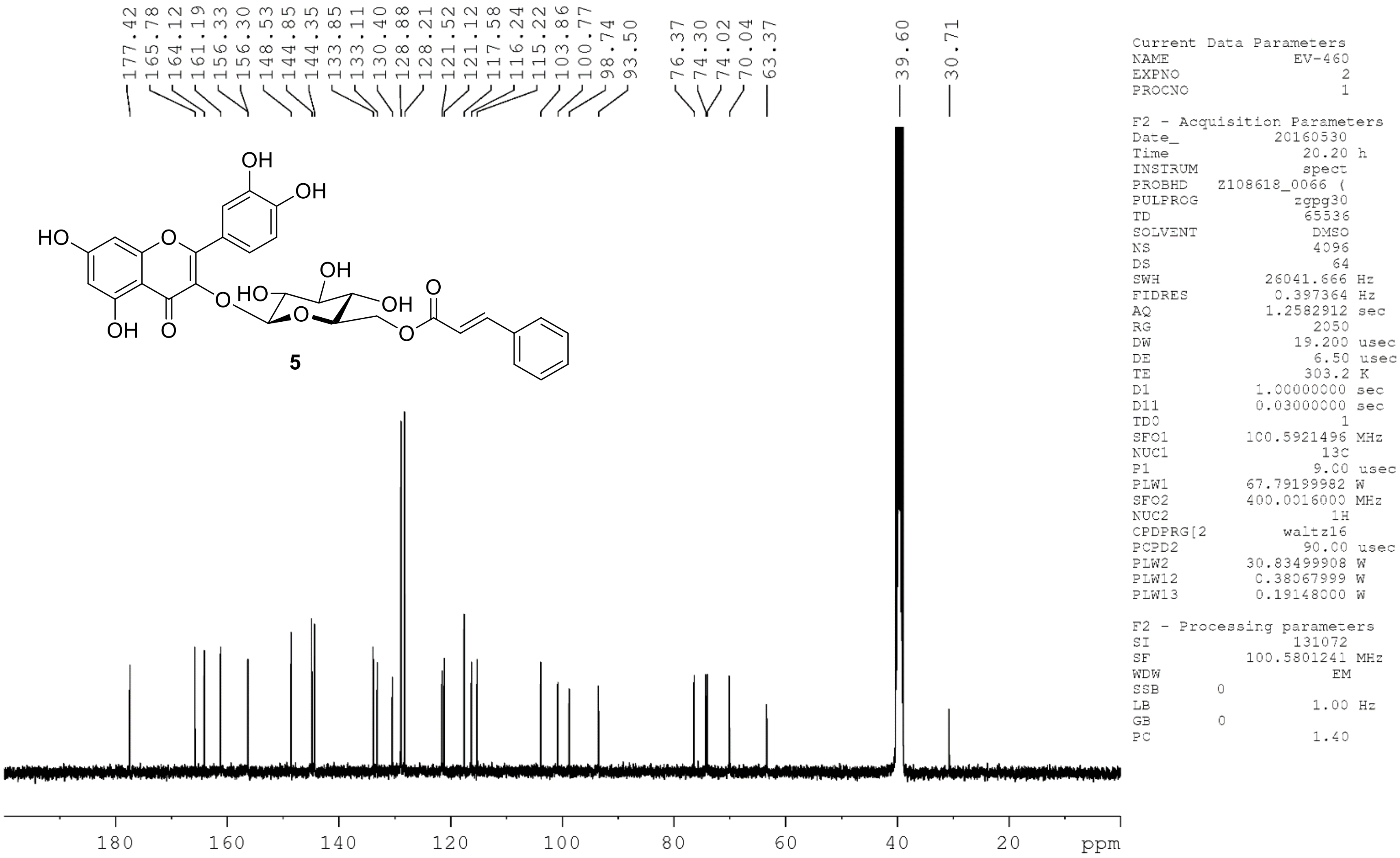

Figure S7. ^{13}C NMR spectrum of compound **5** (DMSO-d_6, 303.2 K).

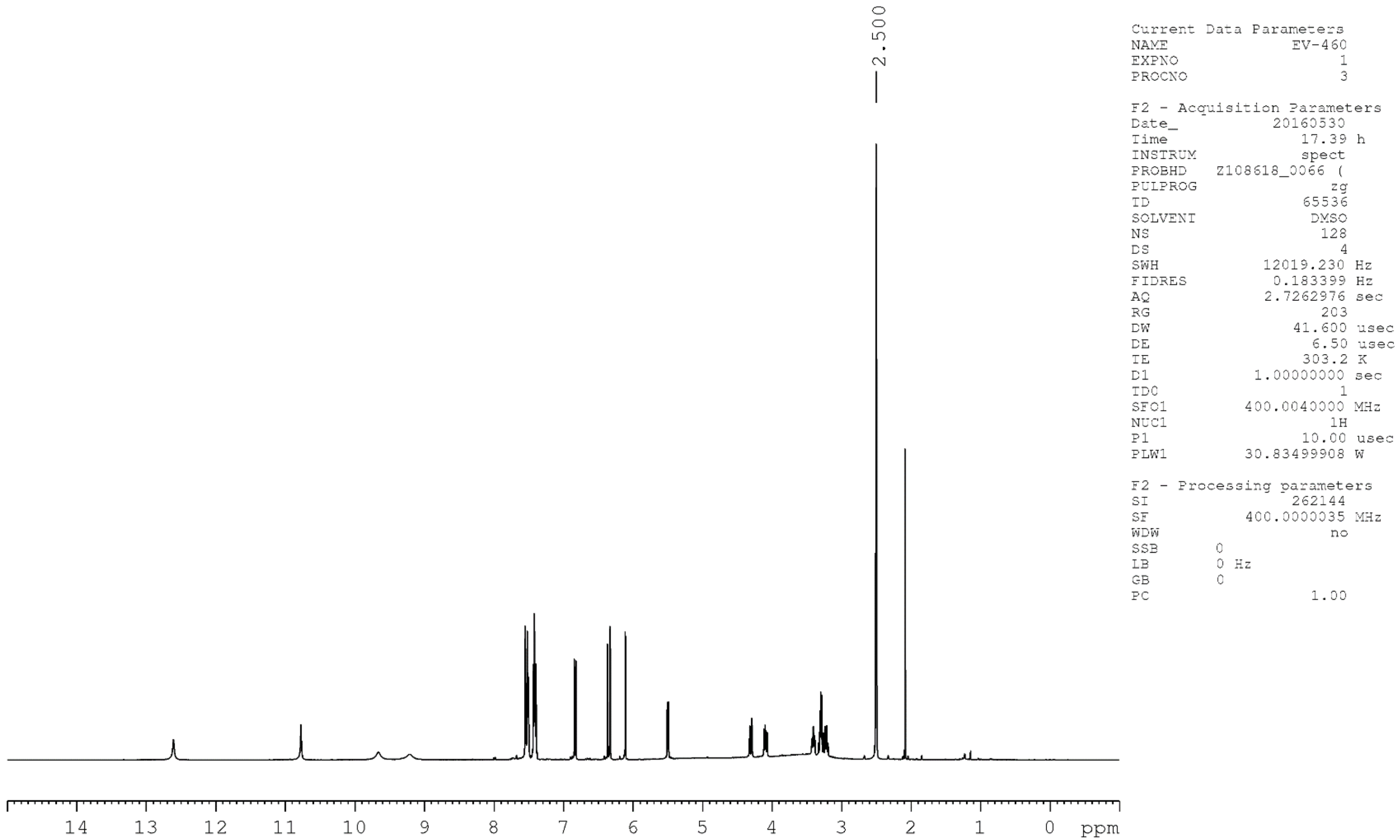

Figure S8. ^1H NMR spectrum of compound **5** (DMSO-d_6, 303.2 K).

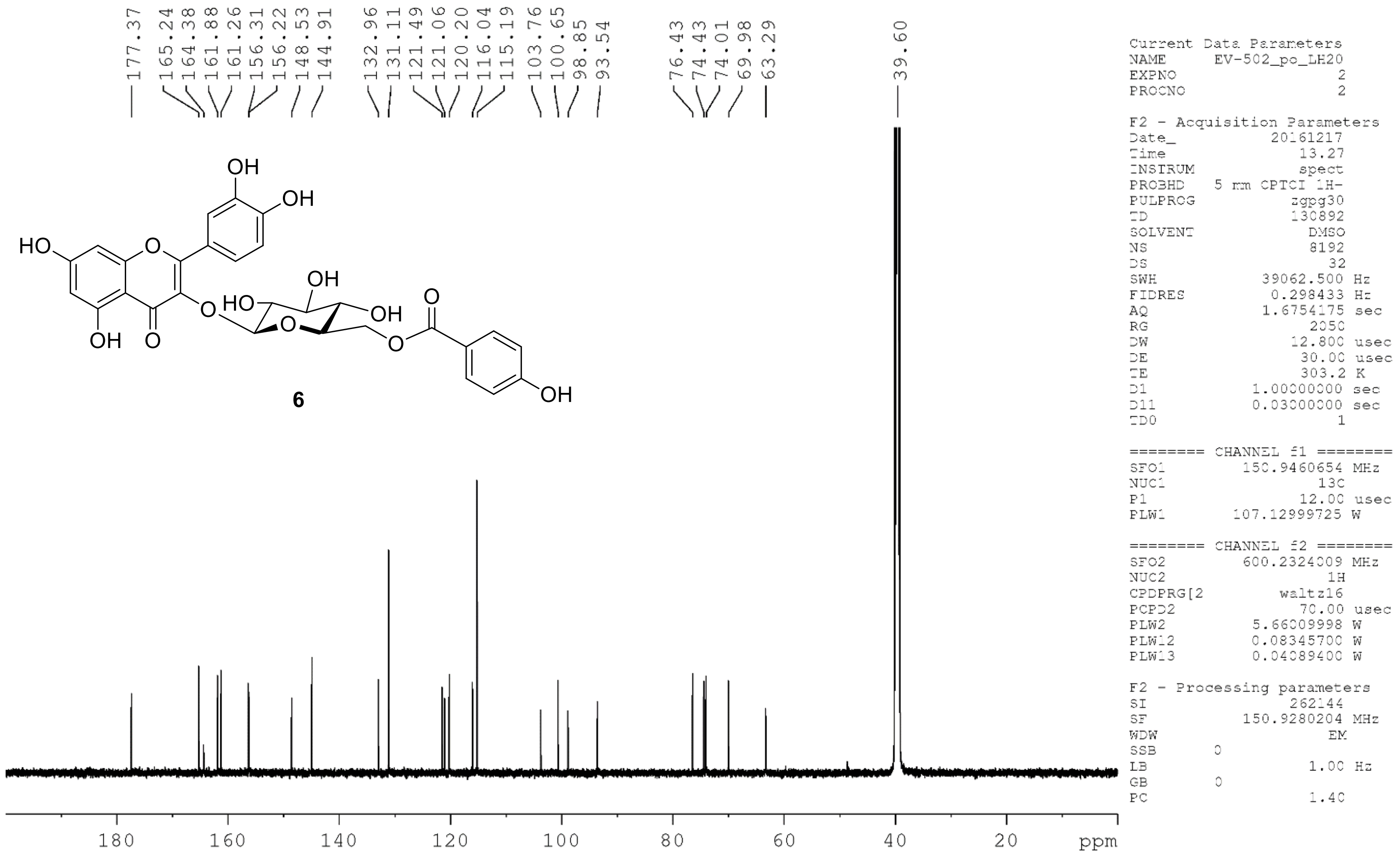

Figure S9. ^{13}C NMR spectrum of compound **6** (DMSO-d_6, 303.2 K).

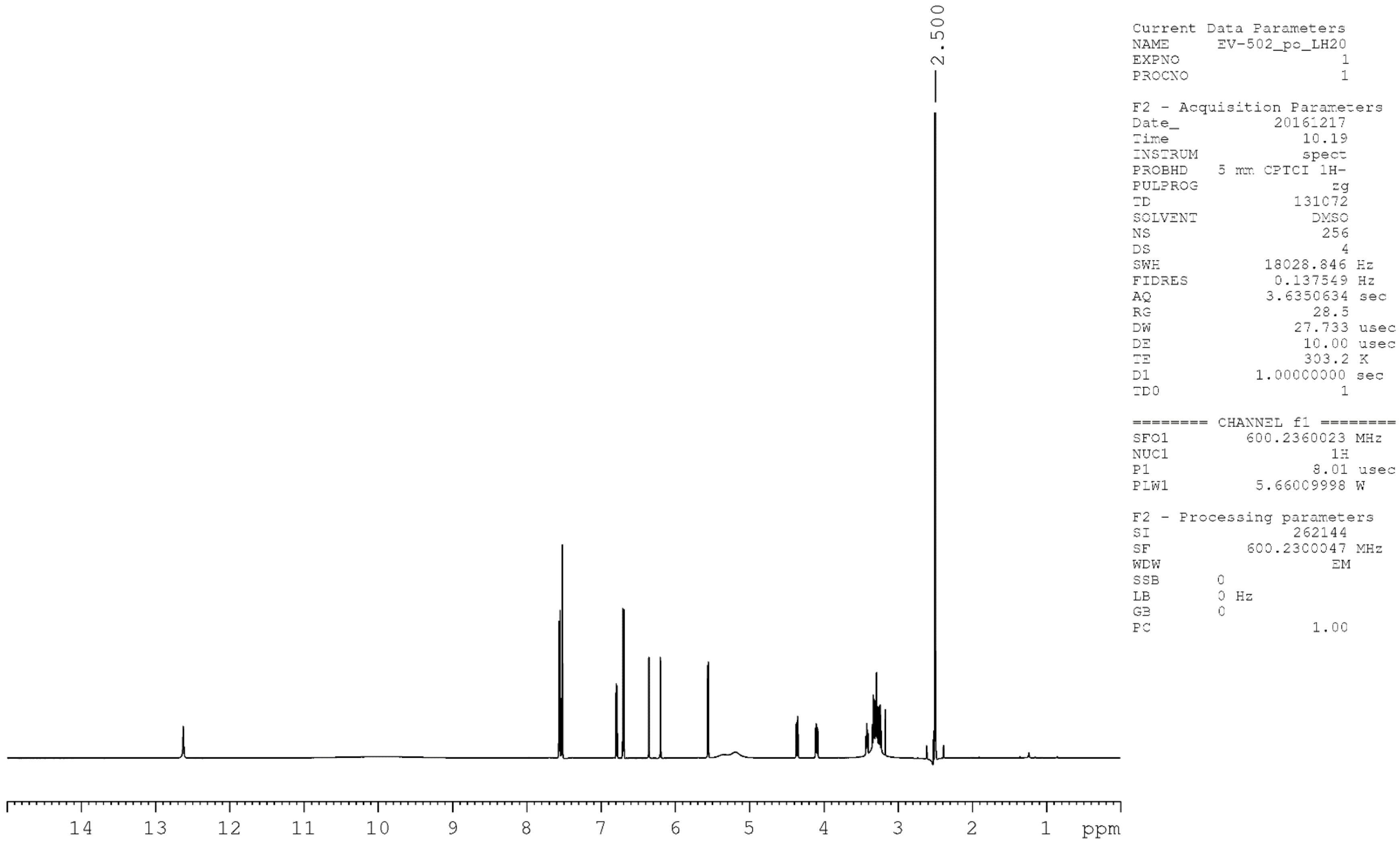

Figure S10. ^{1}H NMR spectrum of compound **6** (DMSO-d_6, 303.2 K).

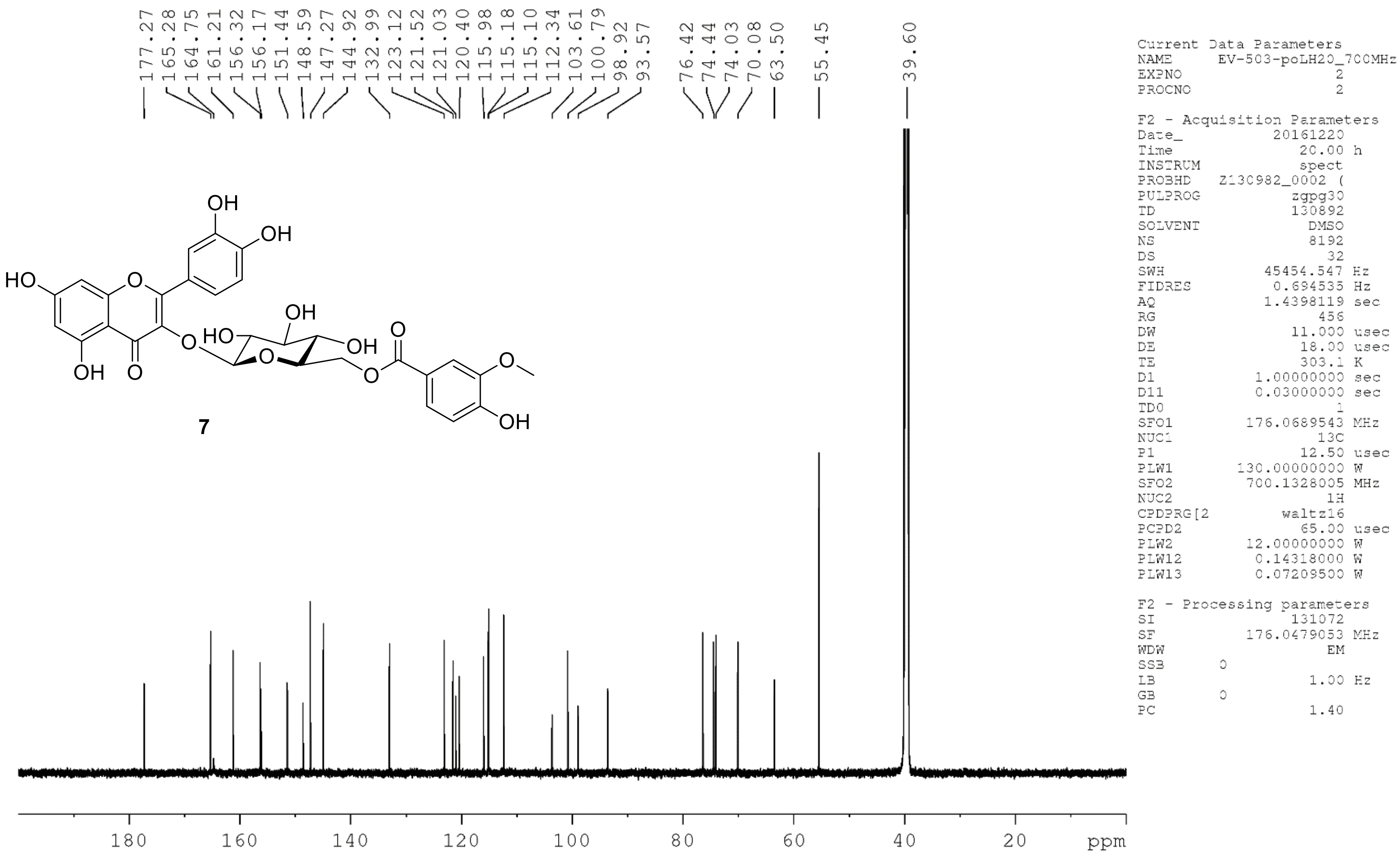

Figure S11. ^{13}C NMR spectrum of compound **7** (DMSO-d_6, 303.2 K).

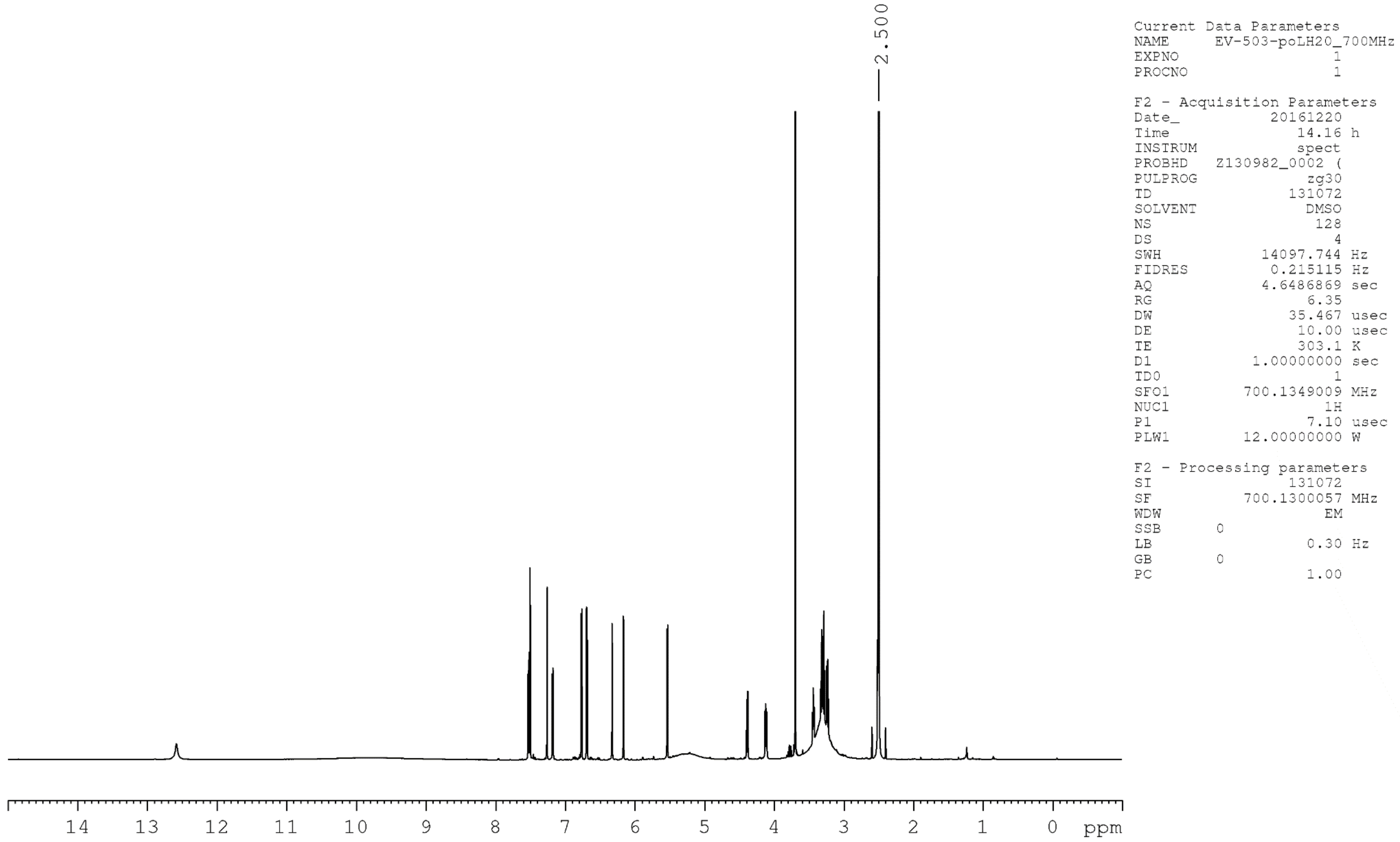

Figure S12. ^{1}H NMR spectrum of compound **7** (DMSO-d_6, 303.2 K).

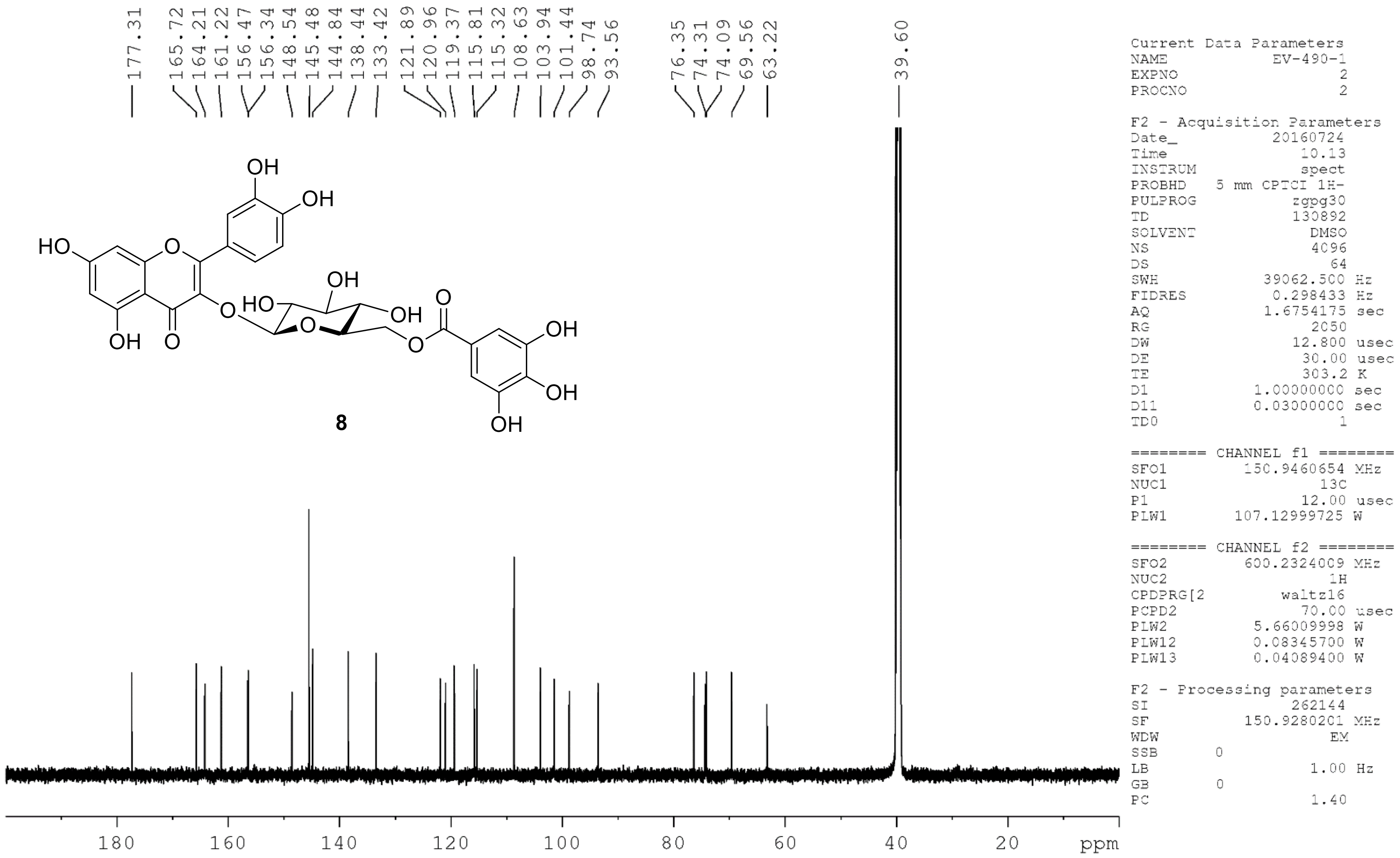

Figure S13. ^{13}C NMR spectrum of compound **8** (DMSO-d_6, 303.2 K).

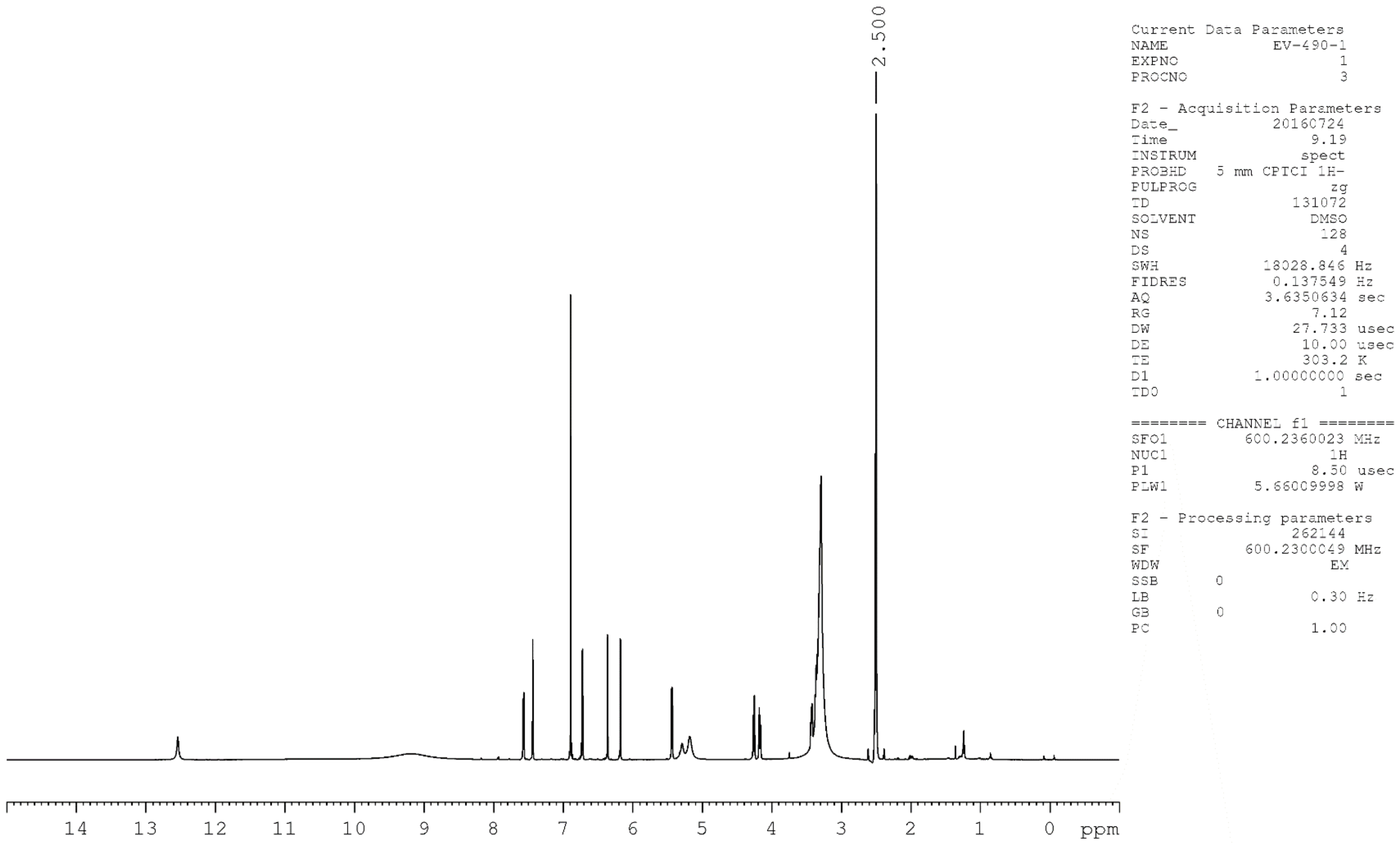

Figure S14. ^{1}H NMR spectrum of compound **8** (DMSO-d_6, 303.2 K).

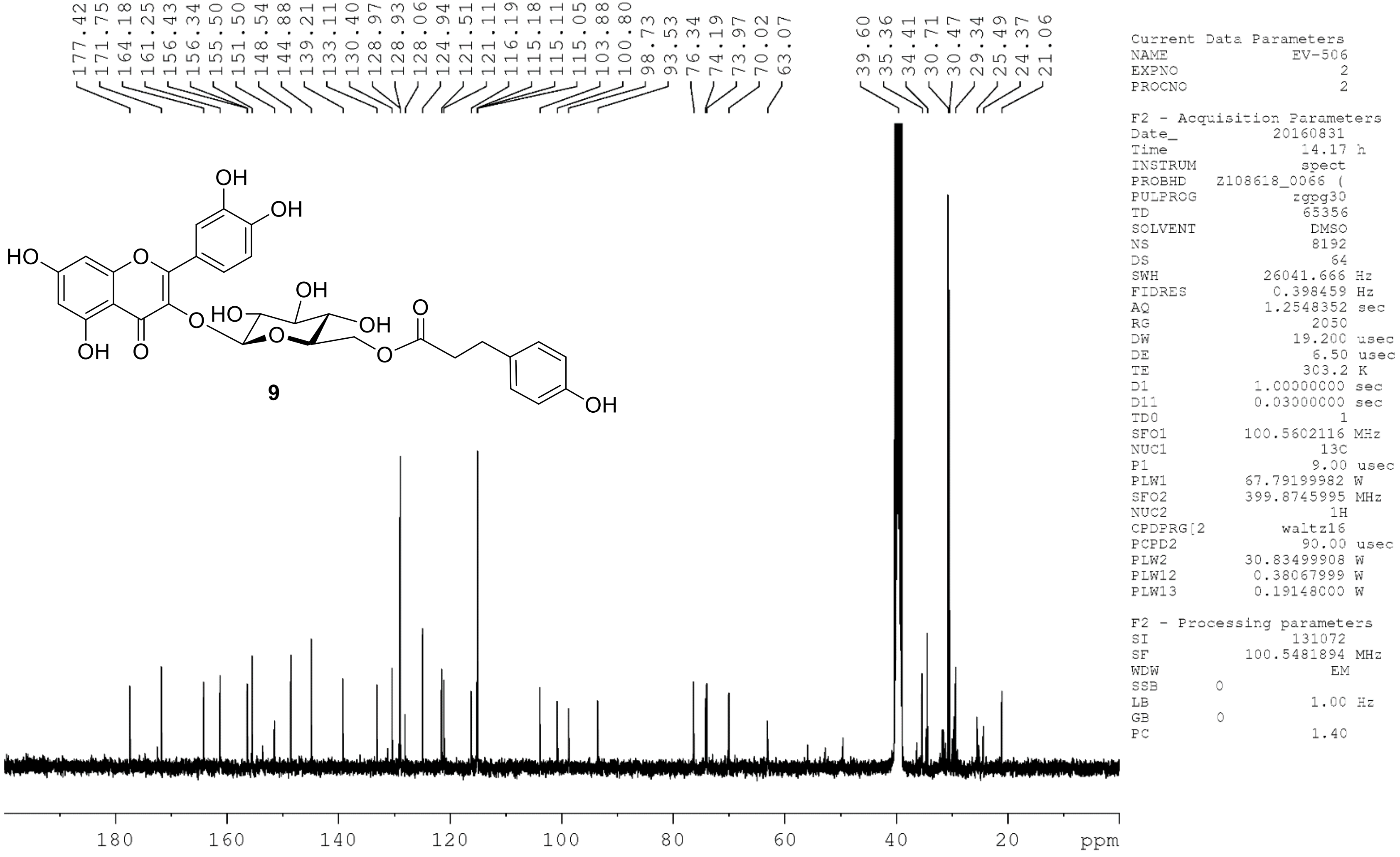

Figure S15. ^{13}C NMR spectrum of compound **9** (DMSO-d_6, 303.2 K).

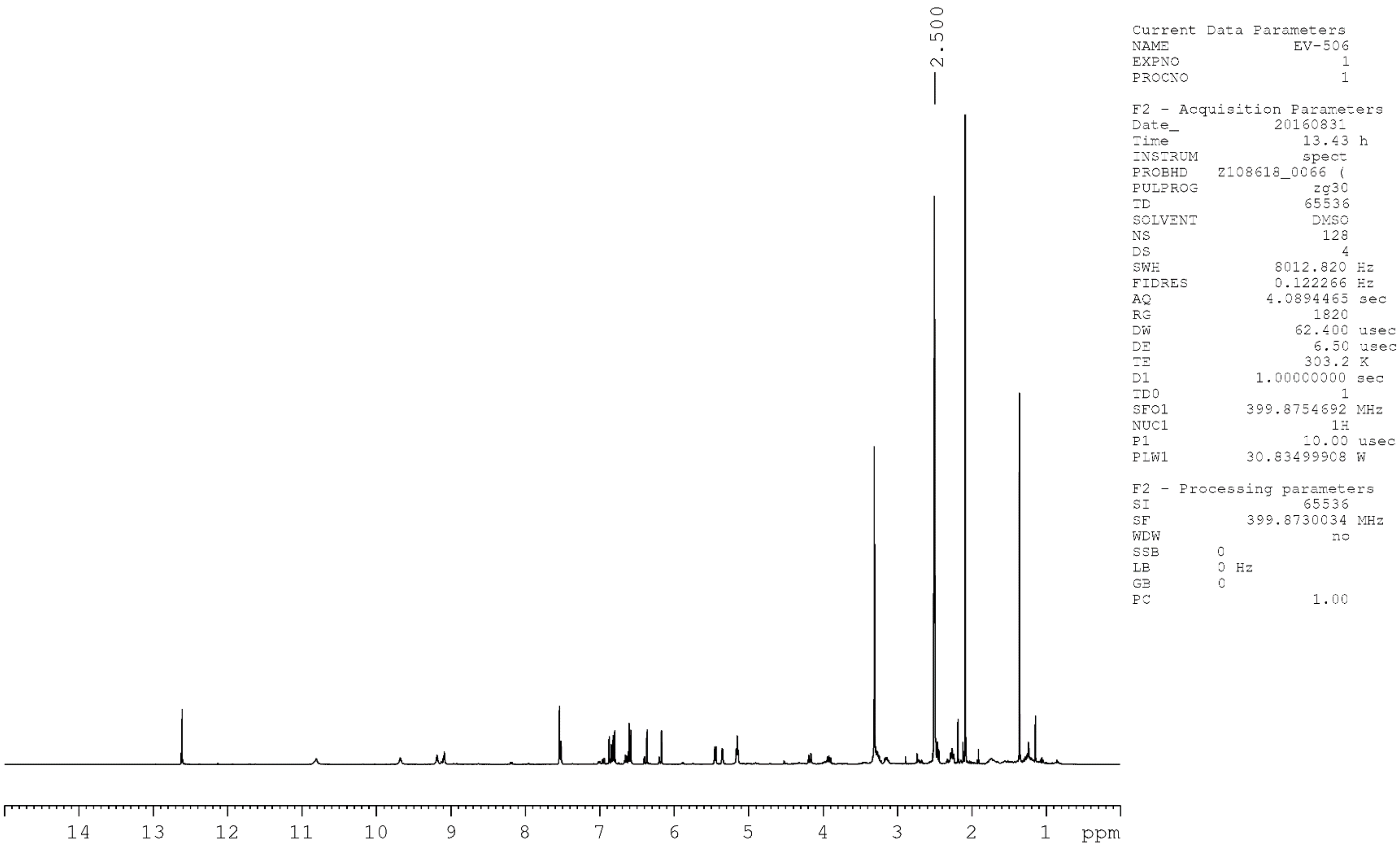

Figure S16. ^{1}H NMR spectrum of compound **9** (DMSO-d_6, 303.2 K).

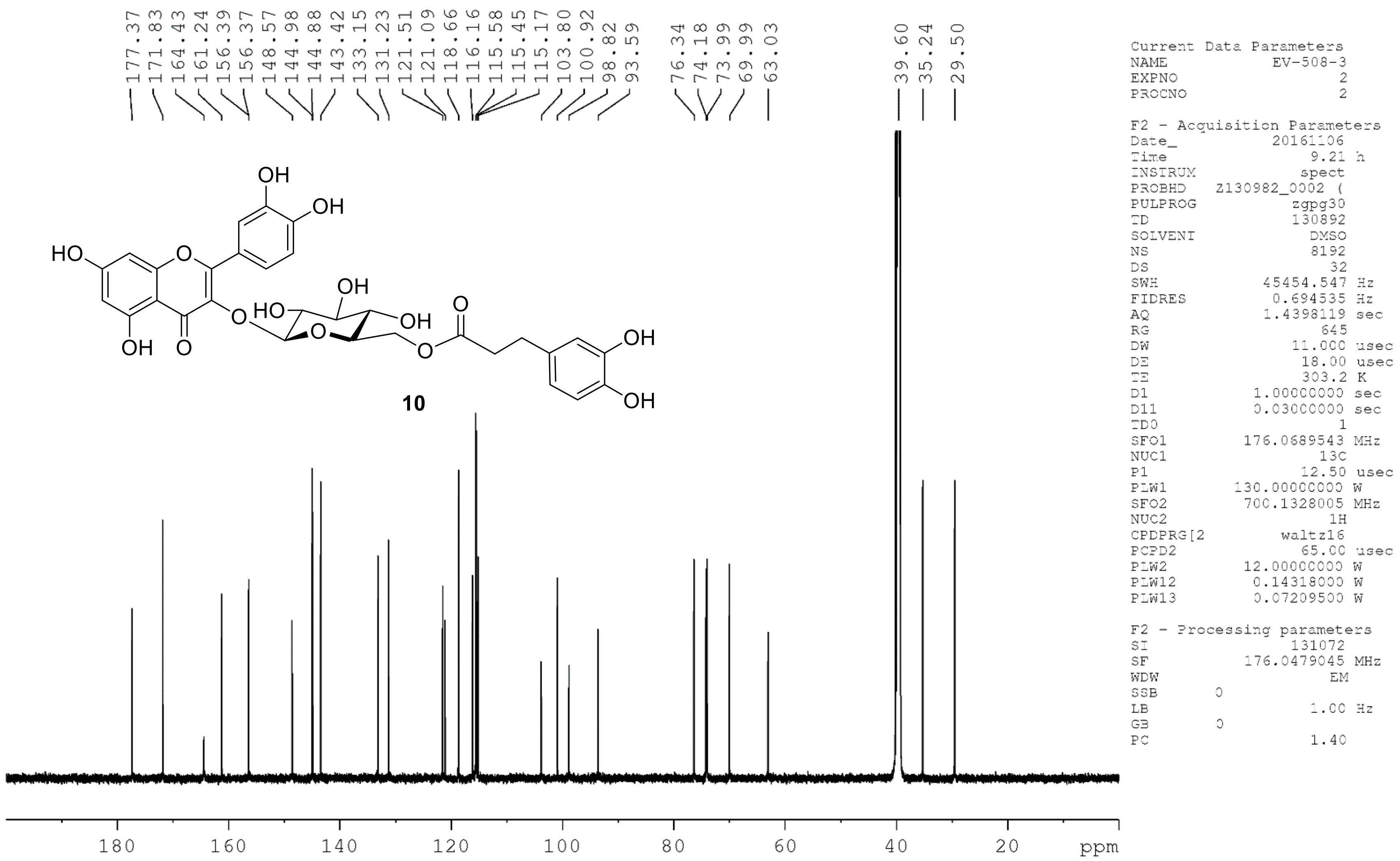

Figure S17. ^{13}C NMR spectrum of compound **10** (DMSO-d_6, 303.2 K).

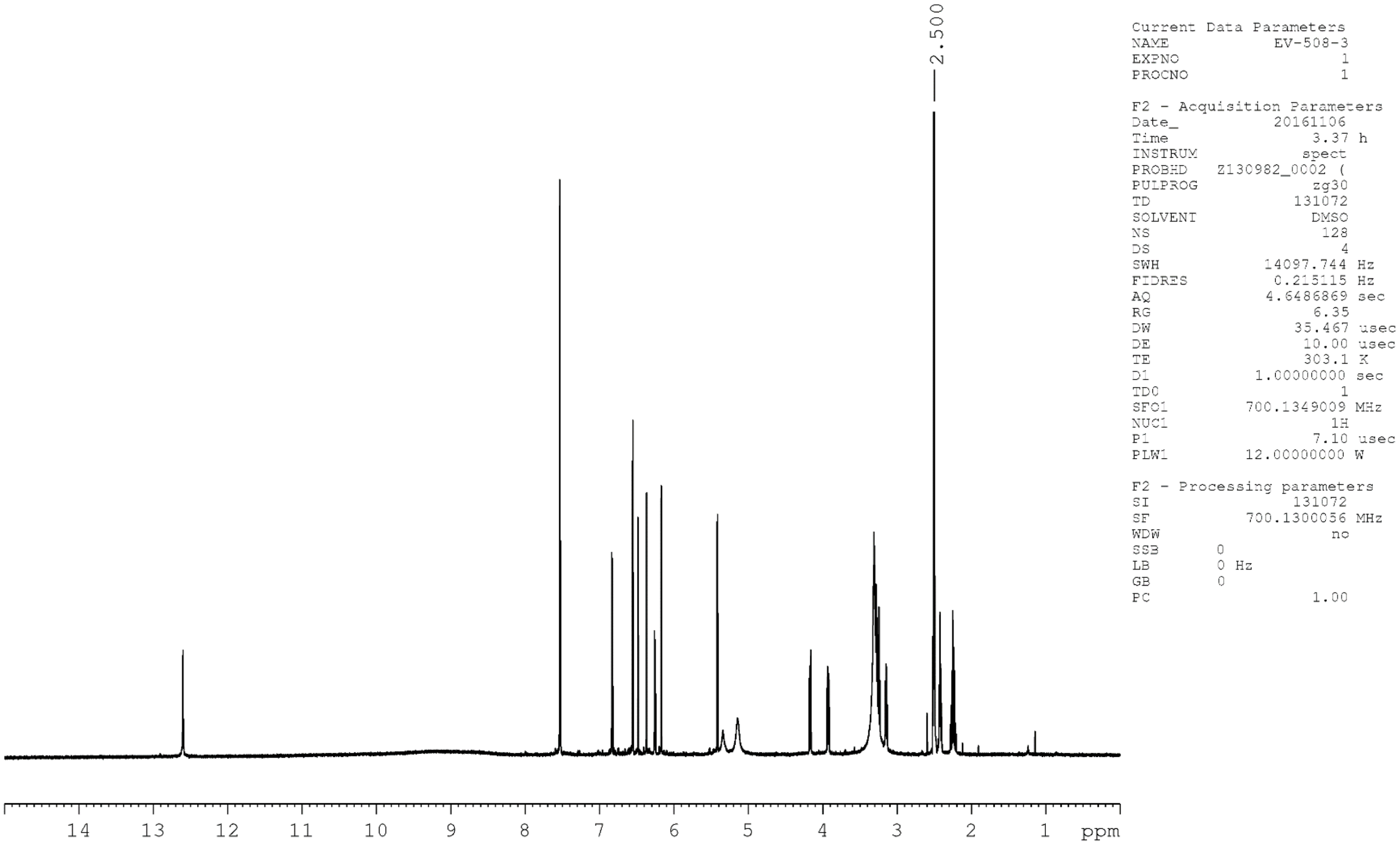

Figure S18. ^{1}H NMR spectrum of compound **10** (DMSO-d_6, 303.2 K).

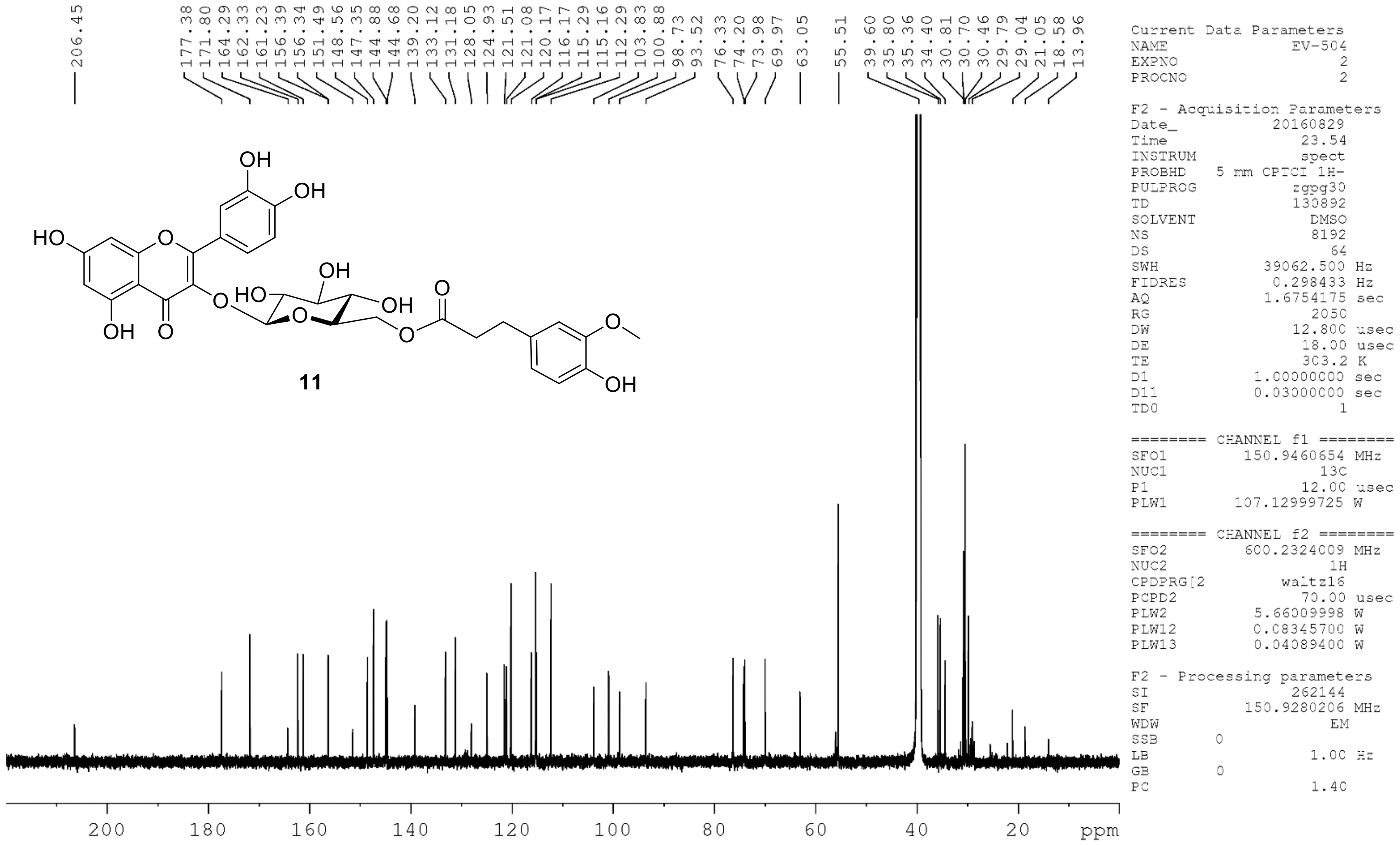

Figure S19. ^{13}C NMR spectrum of compound **11** (DMSO-d_6, 303.2 K).

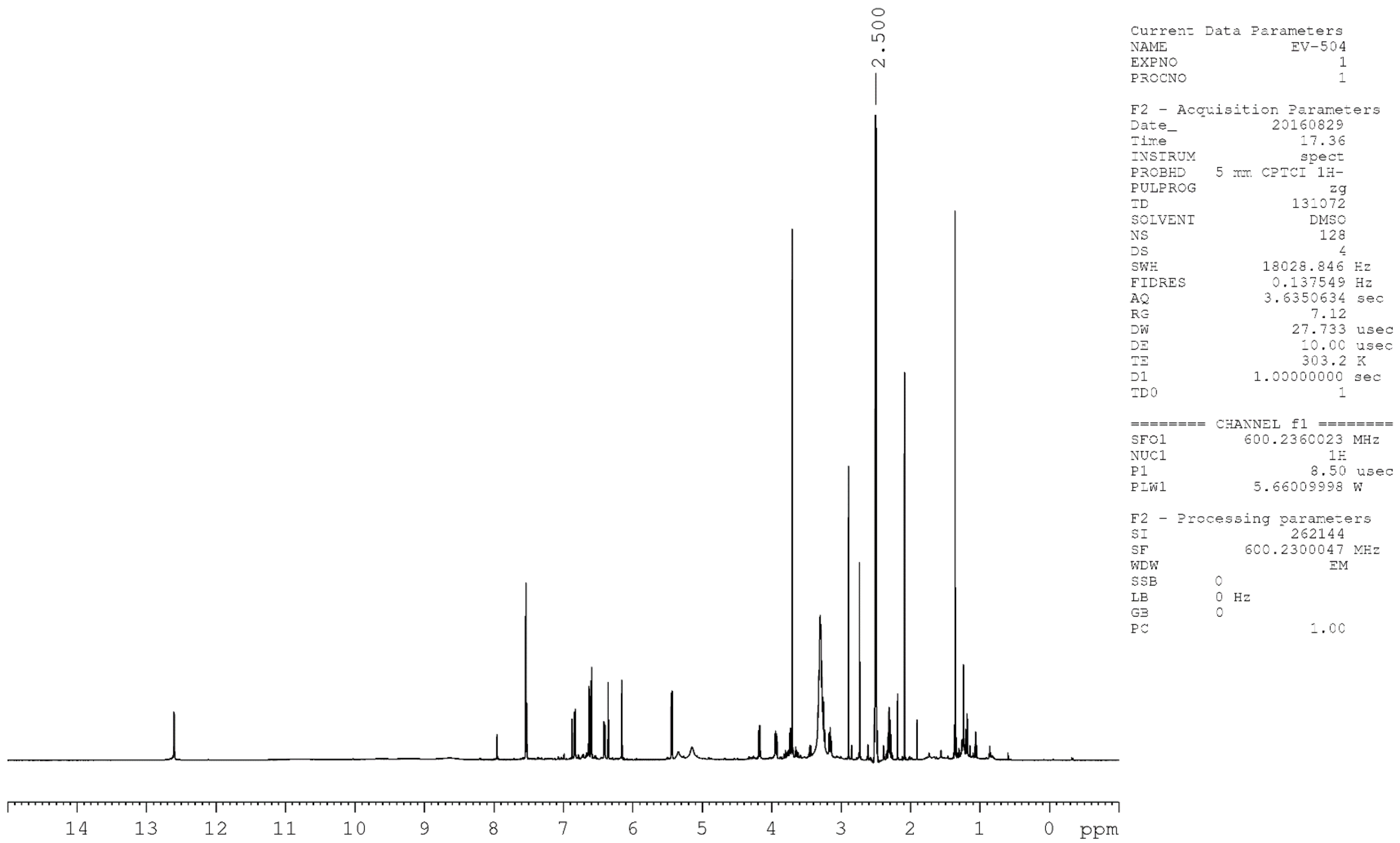

Figure S20. ^{1}H NMR spectrum of compound **11** (DMSO-d_6, 303.2 K).

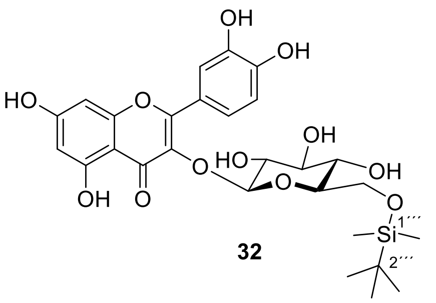

32

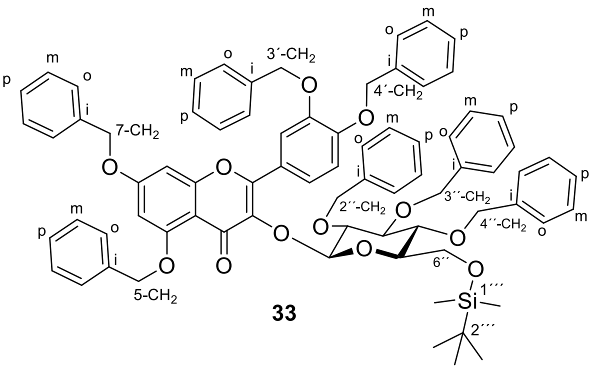

33

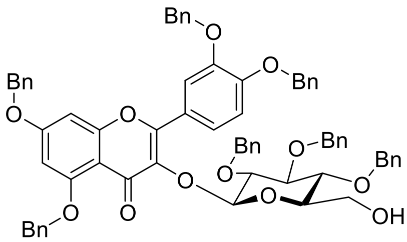

34

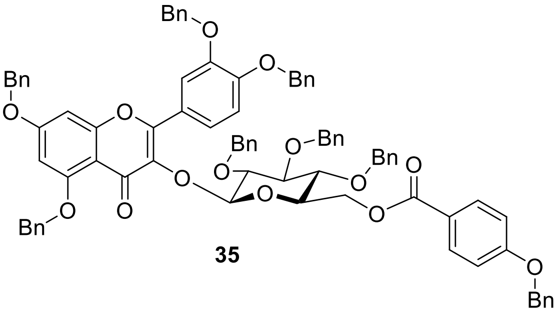

35

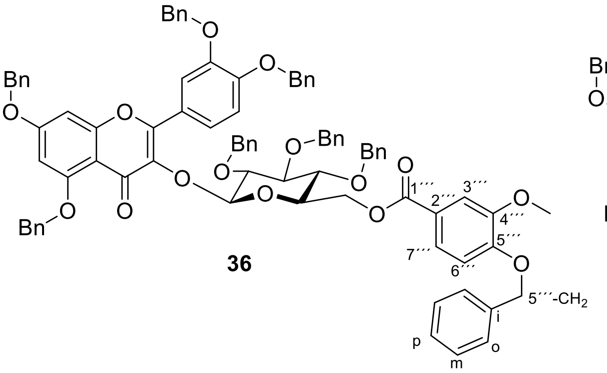

36

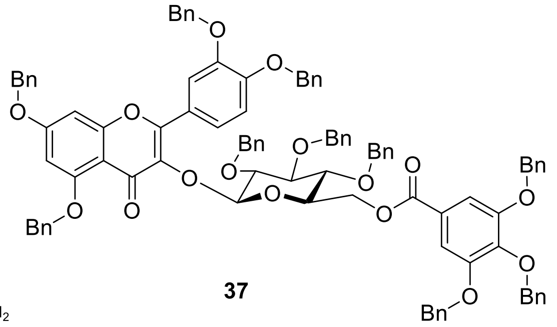

37

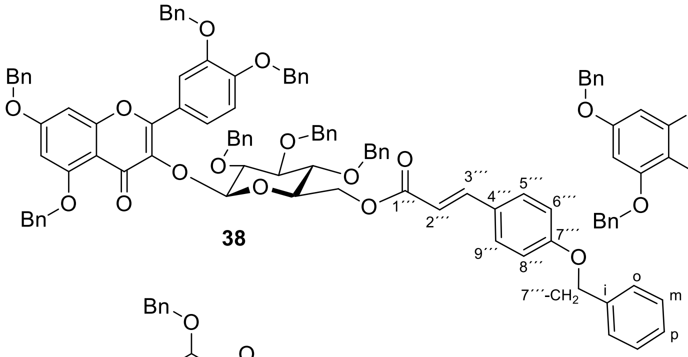

38

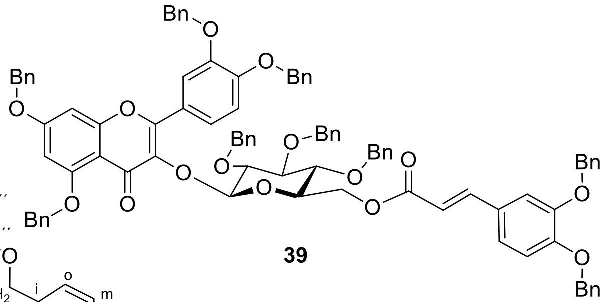

39

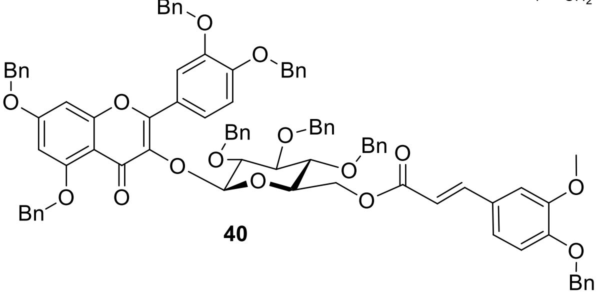

40

Figure S21. Structures of the intermediates **32** - **40**

Table S7. ^{13}C NMR data of the intermediates **32** - **34** (DMSO-d_6, 303.2 K), **33** (acetone, 293.2 K).

**Comp.**	**32**		**33**		**34**	
**Atom number**	**δ**	**m**	**δ**	**m**	**δ**	**m**
**2**	155.97	s	154.36	s	152.90	s
**3**	132.99	s	137.57	s	135.42	s
**4**	177.36	s	173.60	s	171.80	s
**4a**	103.80	s	111.04	s	108.86	s
**5**	161.34	s	161.33	s	159.24	s
**6**	98.78	d	99.26	d	97.99	d
**7**	164.89	s	164.63	s	162.75	s
**8**	93.42	d	95.53	d	94.21	d
**8a**	156.46	s	160.23	s	158.18	s
**1′**	121.15	s	125.43	s	123.16	s
**2′**	116.05	d	116.96	d	115.01	d
**3′**	144.95	s	149.76	s	147.65	s
**4′**	148.60	s	152.25	s	150.00	s
**5′**	115.21	d	115.06	d	113.58	d
**6′**	121.57	d	123.91	d	121.76	d
**1″**	100.17	d	102.21	d	100.09	d
**2″**	74.03	d	84.22	d	82.03	d
**3″**	76.62	d	86.00	d	83.40	d
**4″**	69.96	d	79.18	d	77.35	d
**5″**	77.72	d	77.29	d	75.34	d
**6″**	62.95	t	63.60	t	59.70	t
**1‴**	-	-	-	-	-	-
**2‴**	17.65	s	19.39	s	-	-
**1‴-CH_3a**	-5.84	q	-4.86	q	-	-
**1‴-CH_3b**	-5.72	q	-4.59	q	-	-
**2‴-CH_3**	25.43	q	26.84	q	-	-
**5-CH_2**	-	-	71.85	t	70.13	t
***i*-**	-	-	138.70	s	136.77	s
***o*-**	-	-	128.38*	d	127.05	d
***m*-**	-	-	129.86*	d	128.39	d
***p*-**	-	-	128.96	d	127.62	d
**7-CH_2**	-	-	71.81	t	70.12	t
***i*-**	-	-	137.98	s	136.12	s
***o*-**	-	-	129.43*	d	128.03	d
***m*-**	-	-	130.08*	d	128.57	d
***p*-**	-	-	129.69	d	128.22	d
**3′-CH_2**	-	-	72.43	t	70.29	t
***i*-**	-	-	138.99	s	137.11	s
***o*-**	-	-	128.76*	d	127.43	d
***m*-**	-	-	129.91*	d	128.39	d
***p*-**	-	-	129.22	d	127.83	d
**4′-CH_2**	-	-	71.87	t	69.94	t
***i*-**	-	-	138.90	s	137.01	s
***o*-**	-	-	128.91*	s	127.47	d
***m*-**	-	-	129.91*	d	128.47	d
***p*-**	-	-	129.33	d	127.88	d
**2″-CH_2**	-	-	75.09	t	73.06	t
***i*-**	-	-	140.47	s	138.42	s
***o*-**	-	-	129.83*	d	127.95	d

Comp.	32		33		34	
Atom number	δ	m	δ	m	δ	m
m-	-	-	129.56*	d	127.99	d
p-	-	-	128.83	d	127.36	d
**3″-CH$_2$**	-	-	76.66	t	74.29	t
i-	-	-	140.81	s	138.78	s
o-	-	-	129.14*	d	127.40	d
m-	-	-	129.67*	d	128.16	d
p-	-	-	128.78	d	127.34	d
**4″-CH$_2$**	-	-	75.94	t	73.84	t
i-	-	-	140.35	s	138.35	s
o-	-	-	128.91*	d	127.75	d
m-	-	-	129.70*	d	128.21	d
p-	-	-	128.95	d	127.54	d

m - the multiplicity of ^{13}C NMR signals was resolved using multiplicity-edited gradient-enhanced ^{1}H-^{13}C HSQC

* - 2C

Table S8. ^{1}H NMR data of the intermediates **32** - **34** (DMSO-d_6, 303.2 K), **33** (acetone, 293.2 K).

**Comp.**	**32**				**33**				**34**			
**Atom number**	**δ**	**n**	**m**	***J* [Hz]**	**δ**	**n**	**m**	***J* [Hz]**	**δ H**	**n**	**m**	***J* [Hz]**
**2**	-	-	-	-	-	-	-	-	-	-	-	-
**3**	-	-	-	-	-	-	-	-	-	-	-	-
**4**	-	-	-	-	-	-	-	-	-	-	-	-
**4a**	-	-	-	-	-	-	-	-	-	-	-	-
**5**	-	-	-	-	-	-	-	-	-	-	-	-
**6**	6.150	1	d	2.0	6.701	1	d	2.3	6.736	1	d	2.3
**7**	-	-	-	-	-	-	-	-	-	-	-	-
**8**	6.356	1	d	2.0	6.869	1	d	2.3	6.931	1	d	2.3
**8a**	-	-	-	-	-	-	-	-	-	-	-	-
**1′**	-	-	-	-	-	-	-	-	-	-	-	-
**2′**	7.526	1	d	2.1	8.138	1	d	2.1	8.137	1	d	2.1
**3′**	-	-	-	-	-	-	-	-	-	-	-	-
**4′**	-	-	-	-	-	-	-	-	-	-	-	-
**5′**	6.821	1	d	8.3	7.217	1	d	8.6	7.247	1	d	8.6
**6′**	7.546	1	dd	8.3, 2.1	7.818	1	dd	8.6, 2.1	7.658	1	dd	8.6, 2.1
**1″**	5.520	1	d	7.6	6.010	1	d	7.7	5.861	1	d	7.6
**2″**	3.239	1	m	-	3.731	1	dd	8.9, 7.7	3.54 [H]	1	m	-
**3″**	3.239	1	m	-	3.831	1	dd	9.0, 8.9	3.787	1	dd	8.8, 8.8
**4″**	3.015	1	m	-	3.602	1	dd	9.8, 9.0	3.54 [H]	1	m	-
**5″**	3.091	1	ddd	9.8, 6.6, 1.5	3.386	1	ddd	9.8, 4.0, 2.2	3.379	1	ddd	9.8, 4.0, 2.1
**6″**	3.771	1	dd	11.4, 1.5	3.747	1	dd	11.5, 2.2	3.578	1	ddd	11.9, 4.6, 2.1
	3.435	1	dd	11.4, 6.6	3.721	1	dd	11.5, 4.0	3.510	1	ddd	11.9, 5.2, 4.0
**5-OH**	12.642	1	s	-	-	-	-	-	-	-	-	-
**7-OH**	n.a.	-	-	-	-	-	-	-	-	-	-	-
**4'-OH**	n.a.	-	-	-	-	-	-	-				
**3′-OH**	n.a.	-	-	-	-	-	-	-				
**2″-OH**	n.a.	-	-	-	-	-	-	-				
**3″-OH**	n.a.	-	-	-	-	-	-	-				
**4″-OH**	n.a.	-	-	-								
**1‴**	-	-	-	-	-	-	-	-	-	-	-	-
**2‴**	-	-	-	-	-	-	-	-	-	-	-	-
**1‴-CH_3a**	-0.326	3	s	-	-0.244	3	s	-	-	-	-	-
**1‴-CH_3b**	-0.252	3	s	-	-0.233	3	s	-	-	-	-	-
**2‴-CH_3**	0.592	9	s	-	0.677	9	s	-	-	-	-	-
**5-CH_2**	-	-	-	-	5.317	1	d	12.6	5.278	1	d	12.8
	-	-	-	-	5.284	1	d	12.6	5.243	1	d	12.8
i-	-	-	-	-	-	-	-	-	-	-	-	-
o-	-	-	-	-	7.761	2	m	-	7.630	2	m	-
m-	-	-	-	-	7.42 [H]	2	m	-	7.419	2	m	-
p-	-	-	-	-	7.33 [H]	1	m	-	7.335	1	m	-
**7-CH_2**	-	-	-	-	5.262	2	s	-	5.255	2	s	-
i-	-	-	-	-	-	-	-	-	-	-	-	-
o-	-	-	-	-	7.53 [H]	2	m	-	7.50 [H]	2	m	-
m-	-	-	-	-	7.43 [H]	2	m	-	7.430	2	m	-
p-	-	-	-	-	7.38 [H]	1	m	-	7.374	1	m	-
**3′-CH_2**	-	-	-	-	5.262	1	d	11.8	5.300	1	d	11.9
	-	-	-	-	5.157	1	d	11.8	5.115	1	d	11.9
i-	-	-	-	-	-	-	-	-	-	-	-	-
o-	-	-	-	-	7.55 [H]	2	m	-	7.50 [H]	2	m	-

Comp.	32				33				34			
**Atom number**	**δ**	**n**	**m**	***J* [Hz]**	**δ**	**n**	**m**	***J* [Hz]**	**δ H**	**n**	**m**	***J* [Hz]**
m-	-	-	-	-	7.36 ^{H}	2	m	-	7.362	2	m	-
p-	-	-	-	-	7.31 ^{H}	1	m	-	7.322	1	m	-
**4’-CH_2**	-	-	-	-	5.288	2	s	-	5.264	2	s	-
i-	-	-	-	-	-	-	-	-	-	-	-	-
o-	-	-	-	-	7.54 ^{H}	2	m	-	7.489	2	m	-
m-	-	-	-	-	7.40 ^{H}	2	m	-	7.402	2	m	-
p-	-	-	-	-	7.34 ^{H}	1	m	-	7.333	1	m	-
**2”-CH_2**	-	-	-	-	5.130	1	d	11.4	4.897	1	d	11.5
	-	-	-	-	4.809	1	d	11.4	4.643	1	d	11.5
i-	-	-	-	-	-	-	-	-	-	-	-	-
o-	-	-	-	-	7.45 ^{H}	2	m	-	7.311	2	m	-
m-	-	-	-	-	7.22 ^{H}	2	m	-	7.21 ^{H}	2	m	-
p-	-	-	-	-	7.21 ^{H}	1	m	-	7.21 ^{H}	1	m	-
**3”-CH_2**	-	-	-	-	5.014	1	d	11.0	4.838	1	d	11.3
	-	-	-	-	4.858	1	d	11.0	4.745	1	d	11.3
i-	-	-	-	-	-	-	-	-	-	-	-	-
o-	-	-	-	-	7.37 ^{H}	2	m	-	7.26 ^{H}	2	m	-
m-	-	-	-	-	7.33 ^{H}	2	m	-	7.292	2	m	-
p-	-	-	-	-	7.29 ^{H}	1	m	-	7.274	1	m	-
**4”-CH_2**	-	-	-	-	4.828	1	d	11.2	4.638	1	d	11.0
	-	-	-	-	4.645	1	d	11.2	4.561	1	d	11.0
i-	-	-	-	-	-	-	-	-	-	-	-	-
o-	-	-	-	-	7.26 ^{H}	2	m	-	7.21 ^{H}	2	m	-
m-	-	-	-	-	7.27 ^{H}	2	m	-	7.271	2	m	-
p-	-	-	-	-	7.24 ^{H}	1	m	-	7.255	1	m	-
**6''-OH**	-	-	-	-	-	-	-	-	4.722	1	dd	5.2, 4.6

n.a. - not assigned due to the broadening of the hydroxyl signals

^{H} - HSQC readout

Table S9. ^{13}C NMR data of the intermediates **35** - **38** (DMSO-d_6, 303.2 K).

**Comp.**	**35**		**36**		**37**		**38**	
**Atom number**	**δ**	**m**	**δ**	**m**	**δ**	**m**	**δ**	**m**
**2**	153.15	s	153.16	s	153.24	s	153.18	s
**3**	135.35	s	135.29	s	135.33	s	135.35	s
**4**	171.65	s	171.65	s	171.58	s	171.68	s
**4a**	108.77	s	108.73	s	108.64	s	108.76	s
**5**	159.22	s	159.17	s	159.05	s	159.16	s
**6**	97.93	d	97.89	d	97.80	d	97.93	d
**7**	162.75	s	162.70	s	162.65	s	162.73	s
**8**	94.22	d	94.09	d	93.94	d	94.14	d
**8a**	158.14	s	158.10	s	158.00	s	158.12	s
**1'**	123.07	s	123.11	s	123.11	s	123.05	s
**2'**	114.78	d	114.80	d	114.81	d	115.16	d
**3'**	147.45	s	147.47	s	147.48	s	147.43	s
**4'**	150.19	s	150.15	s	150.11	s	150.21	s
**5'**	113.23	d	113.27	d	113.05	d	113.36	d
**6'**	122.51	d	122.44	d	122.47	d	122.32	d
**1''**	100.24	d	100.16	d	100.31	d	100.27	d
**2''**	82.08	d	82.01	d	81.88	d	82.00	d
**3''**	83.45	d	83.43	d	83.44	d	83.32	d
**4''**	77.42	d	77.76	d	77.98	d	77.22	d
**5''**	72.05	d	72.19	d	71.91	d	71.94	d
**6''**	62.37	t	62.57	t	62.82	t	61.93	t
**5-CH$_2$**	70.12	t	70.07	t	70.03	t	70.10	t
i-	136.79	s	136.82	s	136.78	s	136.79	s
o-	126.99	d	126.96	d	126.90	d	127.01	d
m-	128.37	d	128.37	d	128.34	d	128.38	d
p-	127.60	d	127.59	d	127.55	d	127.61	d
**7-CH$_2$**	70.20	t	70.17	t	70.02	t	70.10	t
i-	136.00	s	136.00	s	135.97	s	136.06	s
o-	128.04	d	128.06	d	127.98	d	128.04	d
m-	128.52	d	128.51	d	128.45	d	128.55	d
p-	128.20	d	128.19	d	128.12	d	128.22	d
**3'-CH$_2$**	70.51	t	70.49	t	70.47	t	70.51	t
i-	136.99	s	136.99	s	137.00	s	137.01	s
o-	127.24	d	127.27	d	127.27	d	127.15	d
m-	128.37	d	128.39	d	128.37	d	128.43	d
p-	127.82	d	127.83	d	127.80	d	127.84	d
**4'-CH$_2$**	69.91	t	69.91	t	69.88	t	69.86	t
i-	136.79	s	136.84	s	136.75	s	136.84	s
o-	127.51	d	127.50	d	127.48	d	127.41	d
m-	128.43	d	128.43	d	128.37	d	128.38	d
p-	127.89	d	127.89	d	127.84	d	127.82	d
**2''-CH$_2$**	73.13	t	73.10	t	73.09	t	73.11	t
i-	138.40	s	138.37	s	138.38	s	138.40	s
o-	128.05	d	128.04	d	128.05	d	128.06	d
m-	128.07	d	128.06	d	128.05	d	128.05	d
p-	127.45	d	127.45	d	127.44	d	127.44	d
**3''-CH$_2$**	74.62	t	74.59	t	74.63	t	74.52	t
i-	138.63	s	138.63	s	138.63	s	138.67	s
o-	127.54	d	127.53	d	127.57	d	127.49	d

Comp.	35		36		37		38	
**Atom number**	**δ**	**m**	**δ**	**m**	**δ**	**m**	**δ**	**m**
m-	128.23	d	128.23	d	128.23	d	128.23	d
p-	127.47	d	127.46	d	127.48	d	127.45	d
**4''-CH_2**	73.88	t	73.90	t	73.94	t	73.92	t
i-	137.84	s	137.89	s	137.96	s	137.89	s
o-	127.94	d	127.92	d	127.89	d	127.97	d
m-	128.23	d	128.26	d	128.25	d	128.24	d
p-	127.69	d	127.71	d	127.69	d	127.71	d
**1'''**	164.78	s	164.85	s	164.53	s	165.85	s
2'''	121.79	s	121.79	s	124.46	s	114.72	d
3'''	131.00	d	111.73	d	107.86	d	144.27	d
4'''	114.78	d	148.52	s	151.84	s	126.66	s
5'''	162.13	s	151.87	s	141.43	s	129.99	d
**6'''**	-	d	111.94	d	-	-	115.03	d
**7'''**	-	d	122.66	d	-	-	160.18	s
**4'''-MeO**	-	-	55.34	q	-	-	-	-
***4'''*-CH_2**	-	-	-	-	70.07	t	-	-
i-	-	-	-	-	136.63	s	-	-
o-	-	-	-	-	127.35	d	-	-
m-	-	-	-	-	128.41	d	-	-
p-	-	-	-	-	127.86	d	-	-
***5'''*-CH_2**	69.50	t	69.89	t	74.31	t	-	-
i-	136.25	s	136.26	s	137.34	s	-	-
o-	127.77	d	127.90	d	128.07	d	-	-
m-	128.43	d	128.40	d	128.06	d	-	-
p-	128.01	d	128.02	d	127.84	d	-	-
**7'''-CH_2**	-	-	-	-	-	-	69.32	t
i-	-	-	-	-	-	-	136.66	s
o-	-	-	-	-	-	-	127.68	d
m-	-	-	-	-	-	-	128.47	d
p-	-	-	-	-	-	-	127.94	d

m - the multiplicity of ^{13}C NMR signals was resolved using multiplicity-edited gradient-enhanced ^{1}H-^{13}C HSQC

Table S10. ^{1}H NMR data of the intermediates **35** - **36** (DMSO-d_6, 303.2 K).

	**35**				**36**			
**Atom number**	**δ**	**n**	**m**	***J* [Hz]**	**δ**	**n**	**m**	***J* [Hz]**
**2**	-	-	-	-	-	-	-	-
**3**	-	-	-	-	-	-	-	-
**4**	-	-	-	-	-	-	-	-
**4a**	-	-	-	-	-	-	-	-
**5**	-	-	-	-	-	-	-	-
**6**	6.723	1	d	2.3	6.696	1	d	2.2
**7**	-	-	-	-	-	-	-	-
**8**	6.912	1	d	2.3	6.862	1	d	2.2
**8a**	-	-	-	-	-	-	-	-
**1'**	-	-	-	-	-	-	-	-
**2'**	7.857	1	d	2.1	7.844	1	d	2.1
**3'**	-	-	-	-	-	-	-	-
**4'**	-	-	-	-	-	-	-	-
**5'**	7.086	1	d	8.7	7.088	1	d	8.7
**6'**	7.747	1	dd	8.7, 2.1	7.700	1	dd	8.7, 2.1
**1''**	5.791	1	d	7.7	5.804	1	d	7.7
**2''**	3.625	1	dd	8.9, 7.7	3.610	1	dd	8.8, 7.7
**3''**	3.886	1	dd	8.9, 8.9	3.888	1	dd	8.9, 8.8
**4''**	3.531	1	dd	9.8, 8.9	3.506	1	dd	9.7, 8.9
**5''**	3.735	1	ddd	9.8, 5.3, 2.5	3.755	1	ddd	9.7, 6.0, 2.5
**6''**	4.298	1	dd	11.9, 2.5	4.335	1	dd	11.9, 2.5
	4.201	1	dd	11.9, 5.3	4.223	1	dd	11.9, 6.0
**5-CH₂**	5.260	1	d	12.6	5.253	1	d	12.8
	5.218	1	d	12.6	5.209	1	d	12.8
i-	-	-	-	-	-	-	-	-
o-	7.632	2	m	-	7.629	2	m	-
m-	7.41 ^{H}	2	m	-	7.41 ^{H}	2	m	-
p-	7.34 ^{H}	1	m	-	7.33 ^{H}	1	m	-
**7-CH₂**	5.233	1	d	11.4	5.214	2	s	-
	5.209	1	d	11.4				
i-	-	-	-	-	-	-	-	-
o-	7.45 ^{H}	2	m	-	7.44 ^{H}	2	m	-
m-	7.39 ^{H}	2	m	-	7.39 ^{H}	2	m	-
p-	7.36 ^{H}	1	m	-	7.35 ^{H}	1	m	-
**3'-CH₂**	5.111	1	d	12.0	5.110	1	d	11.8
	5.073	1	d	12.0	5.070	1	d	11.8
i-	-	-	-	-	-	-	-	-
o-	7.42 ^{H}	2	m	-	7.42 ^{H}	2	m	-
m-	7.33 ^{H}	2	m	-	7.34 ^{H}	2	m	-
p-	7.31 ^{H}	1	m	-	7.30 ^{H}	1	m	-
**4'-CH₂**	5.132	1	d	11.9	5.144	1	d	12.0
	5.088	1	d	11.9	5.106	1	d	12.0
i-	-	-	-	-	-	-	-	-
o-	7.45 ^{H}	2	m	-	7.45 ^{H}	2	m	-
m-	7.36 ^{H}	2	m	-	7.37 ^{H}	2	m	-
p-	7.31 ^{H}	1	m	-	7.31 ^{H}	1	m	-
**2''-CH₂**	5.024	1	d	11.5	5.004	1	d	11.5
	4.726	1	d	11.5	4.714	1	d	11.5
i-	-	-	-	-	-	-	-	-

	35				36			
Atom number	δ	n	m	*J* [Hz]	δ	n	m	*J* [Hz]
o-	7.39 [H]	2	m	-	7.37 [H]	2	m	-
m-	7.24 [H]	2	m	-	7.23 [H]	2	m	-
p-	7.24 [H]	1	m	-	7.23 [H]	1	m	-
**3''-CH₂**	4.923	1	d	11.1	4.917	1	d	11.2
	4.788	1	d	11.1	4.784	1	d	11.2
i-	-	-	-	-	-	-	-	-
o-	7.33 [H]	2	m	-	7.33 [H]	2	m	-
m-	7.34 [H]	2	m	-	7.34 [H]	2	m	-
p-	7.30 [H]	1	m	-	7.31 [H]	1	m	-
**4''-CH₂**	4.718	1	d	11.1	4.726	1	d	11.1
	4.502	1	d	11.1	4.530	1	d	11.1
i-	-	-	-	-	-	-	-	-
o-	7.18 [H]	2	m	-	7.20 [H]	2	m	-
m-	7.21 [H]	2	m	-	7.24 [H]	2	m	-
p-	7.20 [H]	1	m	-	7.22 [H]	1	m	-
**1'''**					-	-	-	-
**2'''**	-	-	-	-	-	-	-	-
**3'''**	7.624	2	m	-	7.218	1	m	-
**4'''**	6.872	2	m	-	-	-	-	-
**5'''**	-	-	-	-	-	-	-	-
**6'''**	-	-	-	-	6.768	1	m	-
**7'''**	-	-	-	-	7.213	1	m	-
**4'''-MeO**	-	-	-	-	3.620	3	s	-
**5'''-CH₂**	5.001	1	d	11.9	4.961	1	d	11.6
	4.982	1	d	11.9	4.934	1	d	11.6
i-	-	-	-	-	-	-	-	-
o-	7.33 [H]	2	m	-	7.33 [H]	2	m	-
m-	7.34 [H]	2	m	-	7.33 [H]	2	m	-
p-	7.33 [H]	1	m	-	7.32 [H]	1	m	-

[H] - HSQC readout

Table S11. ^{1}H NMR data of the intermediates **37** - **38** (DMSO-d_6, 30 °C).

	**37**				**38**			
**Atom number**	**δ**	**n**	**m**	***J* [Hz]**	**δ**	**n**	**m**	***J* [Hz]**
**2**	-	-	-	-	-	-	-	-
**3**	-	-	-	-	-	-	-	-
**4**	-	-	-	-	-	-	-	-
**4a**	-	-	-	-	-	-	-	-
**5**	-	-	-	-	-	-	-	-
**6**	6.611	1	d	2.2	6.660	1	d	2.2
**7**	-	-	-	-	-	-	-	-
**8**	6.712	1	d	2.2	6.839	1	d	2.2
**8a**	-	-	-	-	-	-	-	-
**1'**	-	-	-	-	-	-	-	-
**2'**	7.803	1	d	2.1	7.977	1	d	2.1
**3'**	-	-	-	-	-	-	-	-
**4'**	-	-	-	-	-	-	-	-
**5'**	6.933	1	d	8.6	7.171	1	d	8.6
**6'**	7.624	1	dd	8.6, 2.1	7.712	1	dd	8.6, 2.1
**1''**	5.787	1	d	7.6	5.779	1	d	7.6
**2''**	3.602	1	dd	8.8, 7.6	3.608	1	dd	8.8, 7.6
**3''**	3.888	1	dd	8.8, 8.8	3.877	1	dd	8.9, 8.8
**4''**	3.495	1	dd	9.7, 8.8	3.553	1	dd	9.8, 8.9
**5''**	3.784	1	ddd	9.7, 5.9, 2.7	3.702	1	ddd	9.8, 4.0, 3.2
**6''**	4.393	1	dd	11.9, 2.7	4.163	2	m	-
	4.260	1	dd	11.9, 5.9				
**5-CH$_2$**	5.187	1	d	12.6	5.238	1	d	12.7
	5.109	1	d	12.6	5.189	1	d	12.7
i-	-	-	-	-	-	-	-	-
o-	7.594	2	m	-	7.635	2	m	-
m-	7.40 [H]	2	m	-	7.42 [H]	2	m	-
p-	7.32 [H]	1	m	-	7.34 [H]	1	m	-
**7-CH$_2$**	5.119	1	d	11.4	5.206	1	d	11.6
	5.095	1	d	11.4	5.165	1	d	11.6
i-	-	-	-	-	-	-	-	-
o-	7.35 H	2	m	-	7.495	2	m	-
m-	7.36 H	2	m	-	7.41 [H]	2	m	-
p-	7.35 H	1	m	-	7.36 [H]	1	m	-
**3'-CH$_2$**	5.102	1	d	12.0	5.216	1	d	12.0
	5.067	1	d	12.0	5.145	1	d	12.0
i-	-	-	-	-	-	-	-	-
o-	7.40 [H]	2	m	-	7.461	2	m	-
m-	7.31 [H]	2	m	-	7.33 [H]	2	m	-
p-	7.28 [H]	1	m	-	7.30 [H]	1	m	-
**4'-CH$_2$**	5.102	1	d	12.0	5.114	1	d	12.2
	5.067	1	d	12.0	5.078	1	d	12.2
i-	-	-	-	-	-	-	-	-
o-	7.41 [H]	2	m	-	7.39 [H]	2	m	-
m-	7.32 [H]	2	m	-	7.34 [H]	2	m	-
p-	7.28 [H]	1	m	-	7.30 [H]	1	m	-
**2''-CH$_2$**	5.001	1	d	11.5	5.003	1	d	11.4
	4.704	1	d	11.5	4.695	1	d	11.4
i-	-	-	-	-	-	-	-	-

	37				38			
**Atom number**	**δ**	**n**	**m**	***J* [Hz]**	**δ**	**n**	**m**	***J* [Hz]**
o-	7.37 ^{H}	2	m	-	7.37 ^{H}	2	m	-
m-	7.22 ^{H}	2	m	-	7.23 ^{H}	2	m	-
p-	7.23 ^{H}	1	m	-	7.23 ^{H}	1	m	-
**3''-CH₂**	4.933	1	d	11.0	4.919	1	d	11.1
	4.796	1	d	11.0	4.787	1	d	11.1
i-	-	-	-	-	-	-	-	-
o-	7.34 ^{H}	2	m	-	7.33 ^{H}	2	m	-
m-	7.34 ^{H}	2	m	-	7.34 ^{H}	2	m	-
p-	7.31 ^{H}	1	m	-	7.30 ^{H}	1	m	-
**4''-CH₂**	4.739	1	d	11.1	4.722	1	d	10.9
	4.542	1	d	11.1	4.504	1	d	10.9
i-	-	-	-	-	-	-	-	-
o-	7.20 ^{H}	2	m	-	7,185	2	m	-
m-	7.23 ^{H}	2	m	-	7.23 ^{H}	2	m	-
p-	7.22 ^{H}	1	m	-	7.22 ^{H}	1	m	-
**1'''**	-	-	-	-	-	-	-	-
2'''	-	-	-	-	6.171	1	d	16.0
3'''	7.064	2	s	-	7.355	1	d	16.0
4'''	-	-	-	-	-	-	-	-
5'''	-	-	-	-	7.412	2	m	-
**6'''**	-	-	-	-	6.910	2	m	-
**7'''**	-	-	-	-	-	-	-	-
***4'''*-CH₂**	4.923	2	d	11.8	-	-	-	-
	4.853	2	d	11.8	-	-	-	-
i-	-	-	-	-	-	-	-	-
o-	7.33 ^{H}	4	m	-	-	-	-	-
m-	7.32 ^{H}	4	m	-	-	-	-	-
p-	7.28 ^{H}	2	m	-	-	-	-	-
***5'''*-CH₂**	4.951	2	s	-	-	-	-	-
i-	-	-	-	-	-	-	-	-
o-	7.30 ^{H}	2	m	-	-	-	-	-
m-	7.21 ^{H}	2	m	-	-	-	-	-
p-	7.26 ^{H}	1	m	-	-	-	-	-
**7'''-CH₂**	-	-	-	-	5.031	1	d	11.9
	-	-	-	-	5.011	1	d	11.9
i-	-	-	-	-	-	-	-	-
o-	-	-	-	-	7.39 ^{H}	2	m	-
m-	-	-	-	-	7.39 ^{H}	2	m	-
p-	-	-	-	-	7.34 ^{H}	1	m	-

^{H} - HSQC readout

Table S12. ^{13}C NMR data of the intermediates **39** - **40** (DMSO-d_6, 30 °C).

**Comp.**	**39**		**40**	
**Atom number**	**δ**	**m**	**δ**	**m**
**2**	153.24	s	153.22	s
**3**	135.39	s	135.37	s
**4**	171.68	s	171.68	s
**4a**	108.77	s	108.77	s
**5**	159.15	s	159.16	s
**6**	97.90	d	97.88	d
**7**	162.72	s	162.72	s
**8**	94.10	d	94.11	d
**8a**	158.12	s	158.12	s
**1'**	123.02	s	123.01	s
**2'**	115.08	d	115.14	d
**3'**	147.42	s	147.44	s
**4'**	150.15	s	150.17	s
**5'**	113.28	d	113.29	d
**6'**	122.34	d	122.31	d
**1''**	100.34	d	100.33	d
**2''**	81.99	d	81.97	d
**3''**	83.29	d	83.28	d
**4''**	77.26	d	77.24	d
**5''**	71.94	d	71.93	d
**6''**	61.98	t	61.96	t
**5-CH$_2$**	70.10	t	70.10	t
i-	136.78	s	136.78	s
o-	126.99	d	127.00	d
m-	128.37	d	128.37	d
p-	127.60	d	127.61	d
**7-CH$_2$**	70.09	t	70.09	t
i-	136.03	s	136.06	s
o-	128.05	d	128.06	d
m-	128.52	d	128.55	d
p-	128.19	d	128.22	d
**3'-CH$_2$**	70.54	t	70.60	t
i-	136.98	s	136.97	s
o-	127.32	d	127.36	d
m-	128.44	d	128.45	d
p-	127.87	d	127.91	d
**4'-CH$_2$**	69.78	t	69.81	t
i-	136.78	s	136.78	s
o-	127.38	d	127.40	d
m-	128.35	d	128.36	d
p-	127.80	d	127.82	d
**2''-CH$_2$**	73.11	t	73.10	t
i-	138.40	s	138.40	s
o-	128.06	d	128.06	d
m-	128.05	d	128.04	d
p-	127.43	d	127.44	d
**3''-CH$_2$**	74.49	t	74.47	t
i-	138.65	s	138.65	s
o-	127.49	d	127.48	d

Comp.	39		40	
Atom number	δ	m	δ	m
m-	128.22	d	128.22	d
p-	127.46	d	127.44	d
**4''-CH$_2$**	73.94	t	73.93	t
i-	137.90	s	137.90	s
o-	127.98	d	127.97	d
m-	128.25	d	128.25	d
p-	127.72	d	127.72	d
**1'''**	165.93	s	165.98	s
2'''	115.04	d	114.84	d
3'''	144.64	d	144.80	d
4'''	126.96	s	127.01	s
5'''	112.90	d	110.51	d
**6'''**	148.33	s	149.20	s
**7'''**	150.41	s	150.01	s
**8'''**	113.68	d	112.91	d
**9'''**	123.13	d	122.79	d
**6'''-MeO**	-	-	55.53	q
**6'''-CH$_2$**	70.10	t	-	-
i-	137.09	s	-	-
o-	127.56	d	-	-
m-	128.35	d	-	-
p-	127.79	d	-	-
**7'''-CH$_2$**	69.86	t	69.81	t
i-	136.89	s	136.71	s
o-	127.40	d	127.78	d
m-	128.40	d	128.43	d
p-	127.84	d	127.94	d

m - the multiplicity of ^{13}C NMR signals was resolved using multiplicity-edited gradient-enhanced ^{1}H-^{13}C HSQC

Table S13. ^{1}H NMR data of the intermediates **39** - **40** (DMSO-d_6, 30 °C).

	**39**				**40**			
**Atom number**	**δ**	**n**	**m**	***J* [Hz]**	**δ**	**n**	**m**	***J* [Hz]**
**2**	-	-	-	-	-	-	-	-
**3**	-	-	-	-	-	-	-	-
**4**	-	-	-	-	-	-	-	-
**4a**	-	-	-	-	-	-	-	-
**5**	-	-	-	-	-	-	-	-
**6**	6.647	1	d	2.3	6.654	1	d	2.2
**7**	-	-	-	-	-	-	-	-
**8**	6.799	1	d	2.3	6.807	1	d	2.2
**8a**	-	-	-	-	-	-	-	-
**1'**	-	-	-	-	-	-	-	-
**2'**	7.947	1	d	2.2	7.970	1	d	2.1
**3'**	-	-	-	-	-	-	-	-
**4'**	-	-	-	-	-	-	-	-
**5'**	7.135	1	d	8.7	7.152	1	d	8.6
**6'**	7.701	1	dd	8.7, 2.2	7.699	1	dd	8.6, 2.1
**1''**	5.756	1	d	7.6	5.766	1	d	7.6
**2''**	3.584	1	dd	8.8, 7.6	3.591	1	dd	8.6, 7.6
**3''**	3.871	1	dd	8.8, 8.8	3.872	1	dd	9.2, 8.6
**4''**	3.550	1	dd	9.8, 8.8	3.554	1	dd	9.8, 9.2
**5''**	3.699	1	ddd	9.8, 3.7, 3.5	3.702	1	ddd	9.8, 4.5, 2.5
**6''**	4.180	2	m	-	4.202	1	dd	12.1, 4.5
					4.169	1	dd	12.1, 2.5
**5-CH₂**	5.231	1	d	12.6	5.234	1	d	12.7
	5.176	1	d	12.6	5.180	1	d	12.7
i-	-	-	-	-	-	-	-	-
o-	7.627	2	m	-	7.636	2	m	-
m-	7.41 H	2	m	-	7.42 H	2	m	-
p-	7.33 H	1	m	-	7.34 H	1	m	-
**7-CH₂**	5.182	1	d	11.6	5.192	1	d	11.5
	5.143	1	d	11.6	5.155	1	d	11.5
i-	-	-	-	-	-	-	-	-
o-	7.475	2	m	-	7.494	2	m	-
m-	7.39 H	2	m	-	7.41 H	2	m	-
p-	7.35 H	2	m	-	7.36 H	1	m	-
**3'-CH₂**	5.222	1	d	11.9	5.228	1	d	11.8
	5.153	1	d	11.9	5.148	1	d	11.8
i-	-	-	-	-	-	-	-	-
o-	7.455	2	m	-	7.464	2	m	-
m-	7.32 H	2	m	-	7.34 H	2	m	-
p-	7.29 H	1	m	-	7.31 H	1	m	-
**4'-CH₂**	5.070	1	d	12.1	5.087	1	d	12.2
	5.031	1	d	12.1	5.055	1	d	12.2
i-	-	-	-	-	-	-	-	-
o-	7.34 H	2	m	-	7.35 H	2	m	-
m-	7.31 H	2	m	-	7.33 H	2	m	-
p-	7.27 H	1	m	-	7.29 H	1	m	-
**2''-CH₂**	4.999	1	d	11.4	5.002	1	d	11.4
	4.677	1	d	11.4	4.682	1	d	11.4
i-	-	-	-	-	-	-	-	-

	39				40			
**Atom number**	**δ**	**n**	**m**	***J* [Hz]**	**δ**	**n**	**m**	***J* [Hz]**
o-	7.37 [H]	2	m	-	7.37 [H]	2	m	-
m-	7.23 [H]	2	m	-	7.23 [H]	2	m	-
p-	7.23 [H]	1	m	-	7.23 [H]	1	m	-
**3''-CH_2**	4.906	1	d	11.1	4.906	1	d	11.2
	4.778	1	d	11.1	4.778	1	d	11.2
i-	-	-	-	-	-	-	-	-
o-	7.31 [H]	2	m	-	7.31 [H]	2	m	-
m-	7.32 [H]	2	m	-	7.32 [H]	2	m	-
p-	7.29 [H]	1	m	-	7.29 [H]	1	m	-
**4''-CH_2**	4.726	1	d	11.0	4.725	1	d	11.1
	4.520	1	d	11.0	4.519	1	d	11.1
i-	-	-	-	-	-	-	-	-
o-	7.196	2	m	-	7.20 [H]	2	m	-
m-	7.25 [H]	2	m	-	7.25 [H]	2	m	-
p-	7.22 [H]	1	m	-	7.23 [H]	1	m	-
**1'''**	-	-	-	-	-	-	-	-
2'''	6.293	1	d	16.0	6.294	1	d	16.0
3'''	7.346	1	d	16.0	7.369	1	d	16.0
4'''	-	-	-	-	-	-	-	-
5'''	7.335	1	d	1.9	7.172	1	d	2.0
**6'''**	-	-	-	-	-	-	-	-
**7'''**	-	-	-	-	-	-	-	-
**8'''**	6.945	1	d	8.5	6.925	1	d	8,4
**9'''**	7.012	1	dd	8.5, 1.9	6.992	1	dd	8.4, 2.0
**6'''-MeO**	-	-	-	-	3.670	3	s	-
**6'''-CH_2**	5.087	1	d	11.9	-	-	-	-
	5.001	1	d	11.9	-	-	-	-
i-	-	-	-	-	-	-	-	-
o-	7.39 [H]	2	m	-	-	-	-	-
m-	7.34 [H]	2	m	-	-	-	-	-
p-	7.30 [H]	1	m	-	-	-	-	-
**7'''-CH_2**	5.053	2	m	-	5.014	1	d	12.0
					4.995	1	d	12.0
i-	-	-	-	-	-	-	-	-
o-	7.38 [H]	2	m	-	7.38 [H]	2	m	-
m-	7.36 [H]	2	m	-	7.38 [H]	2	m	-
p-	7.31 [H]	1	m	-	7.33 [H]	1	m	-

[H] - HSQC readout

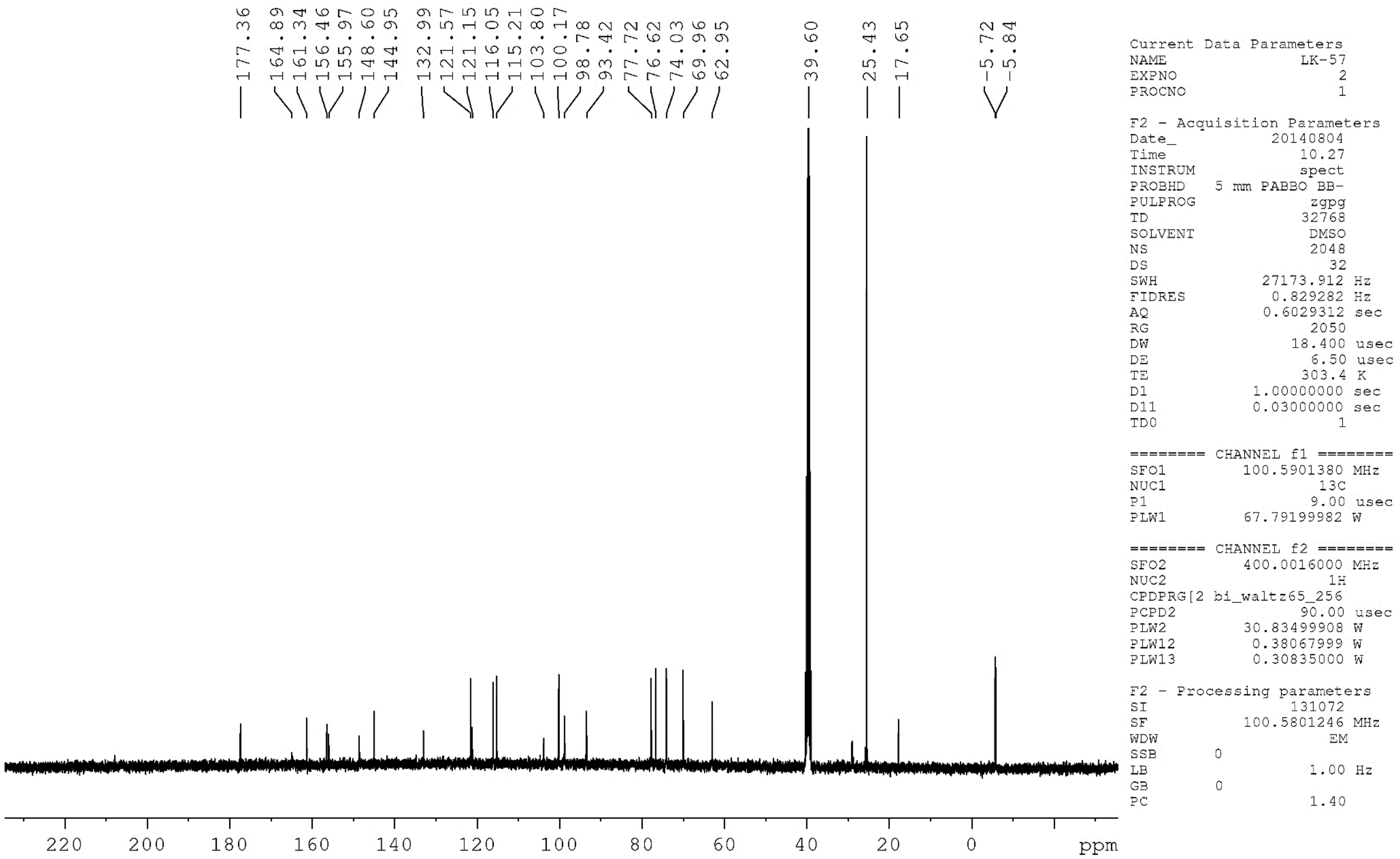

Figure S22. ^{13}C NMR spectrum of compound **32** (DMSO-d_6, 303.2 K).

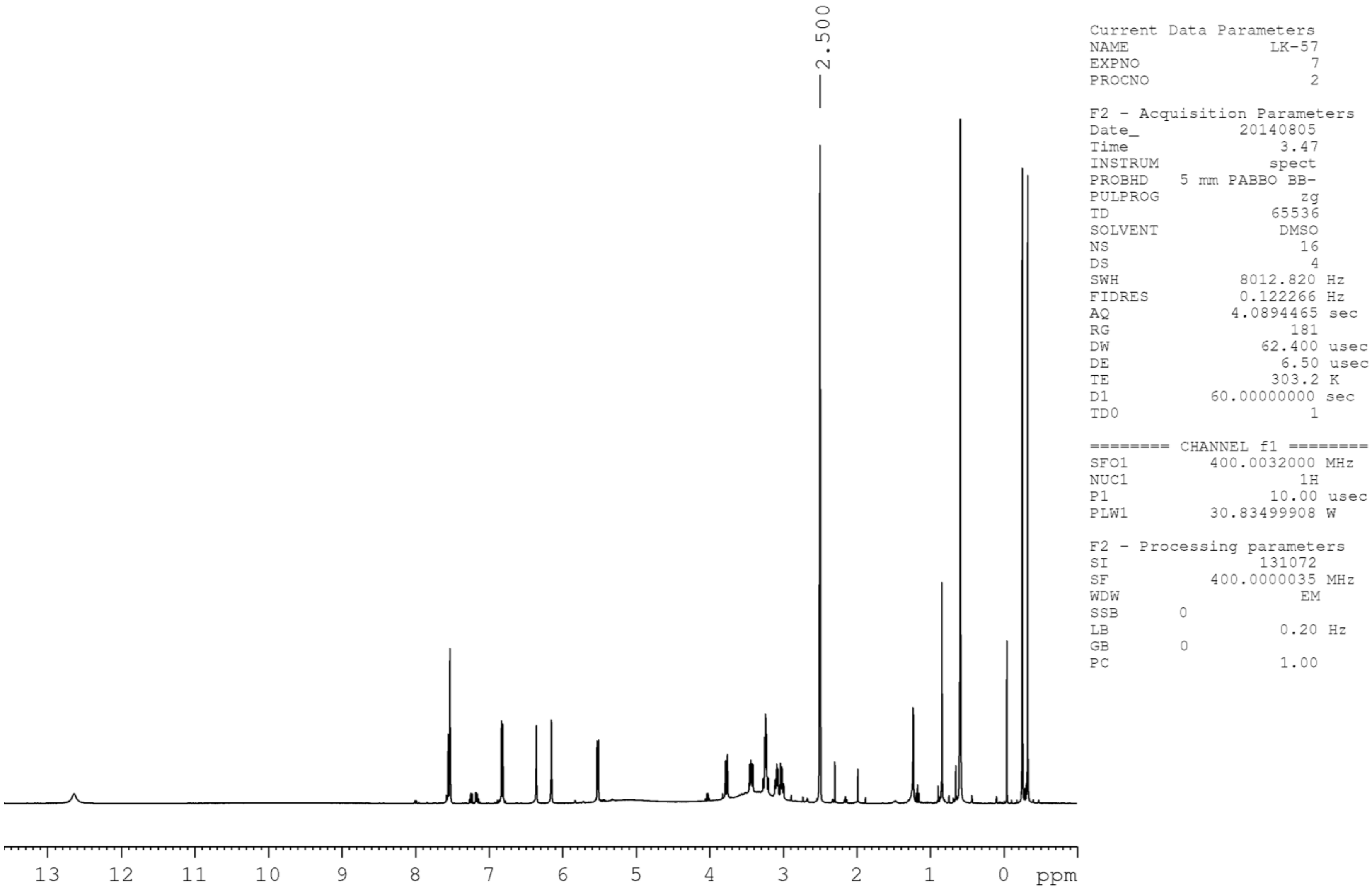

Figure S23. ^{1}H NMR spectrum of compound **32** (DMSO-d_6, 303.2 K).

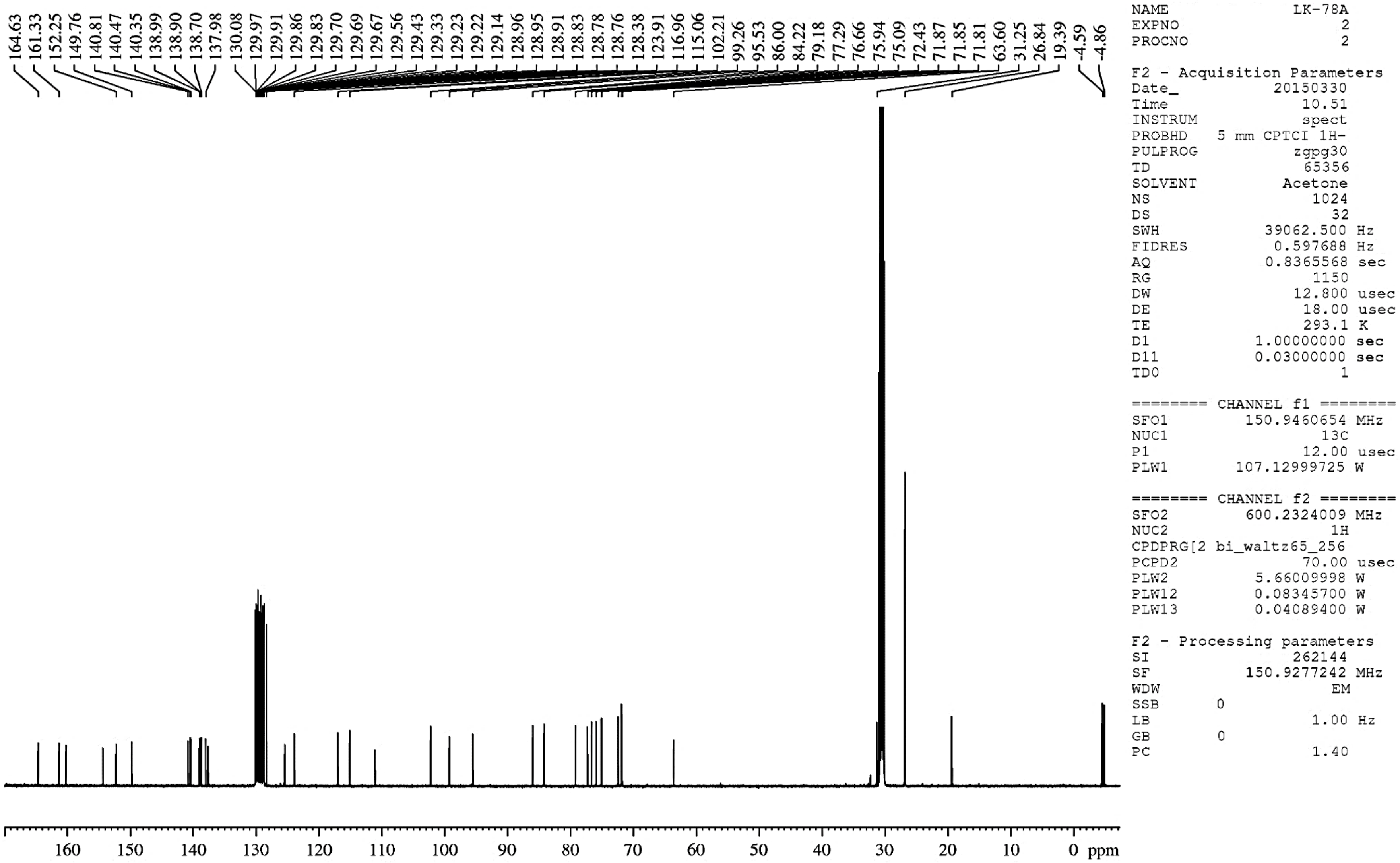

Figure S24. ^{13}C NMR spectrum of compound **33** (acetone, 293.2 K).

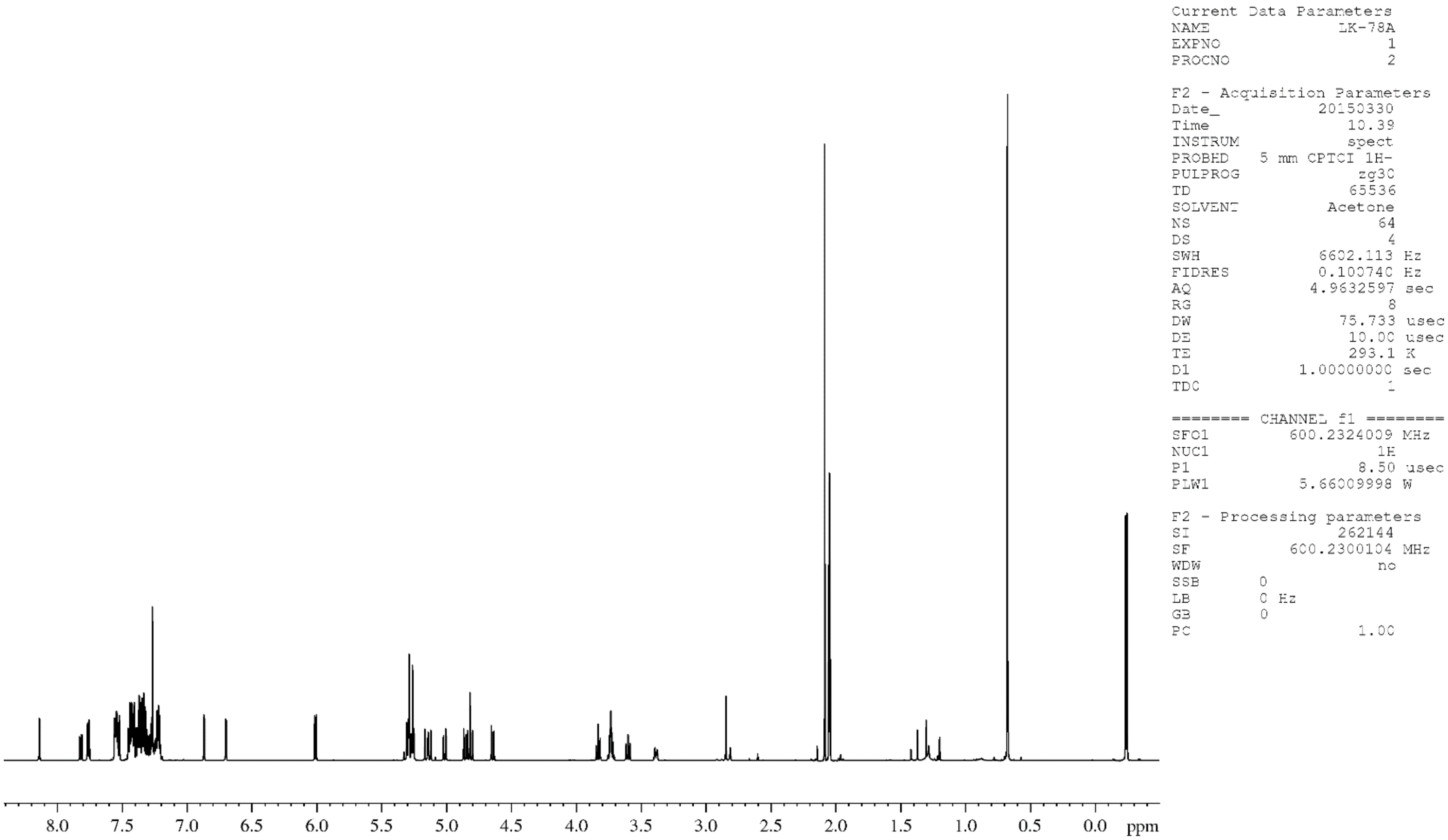

Figure S25. ^{1}H NMR spectrum of compound **33** (acetone, 293.2 K).

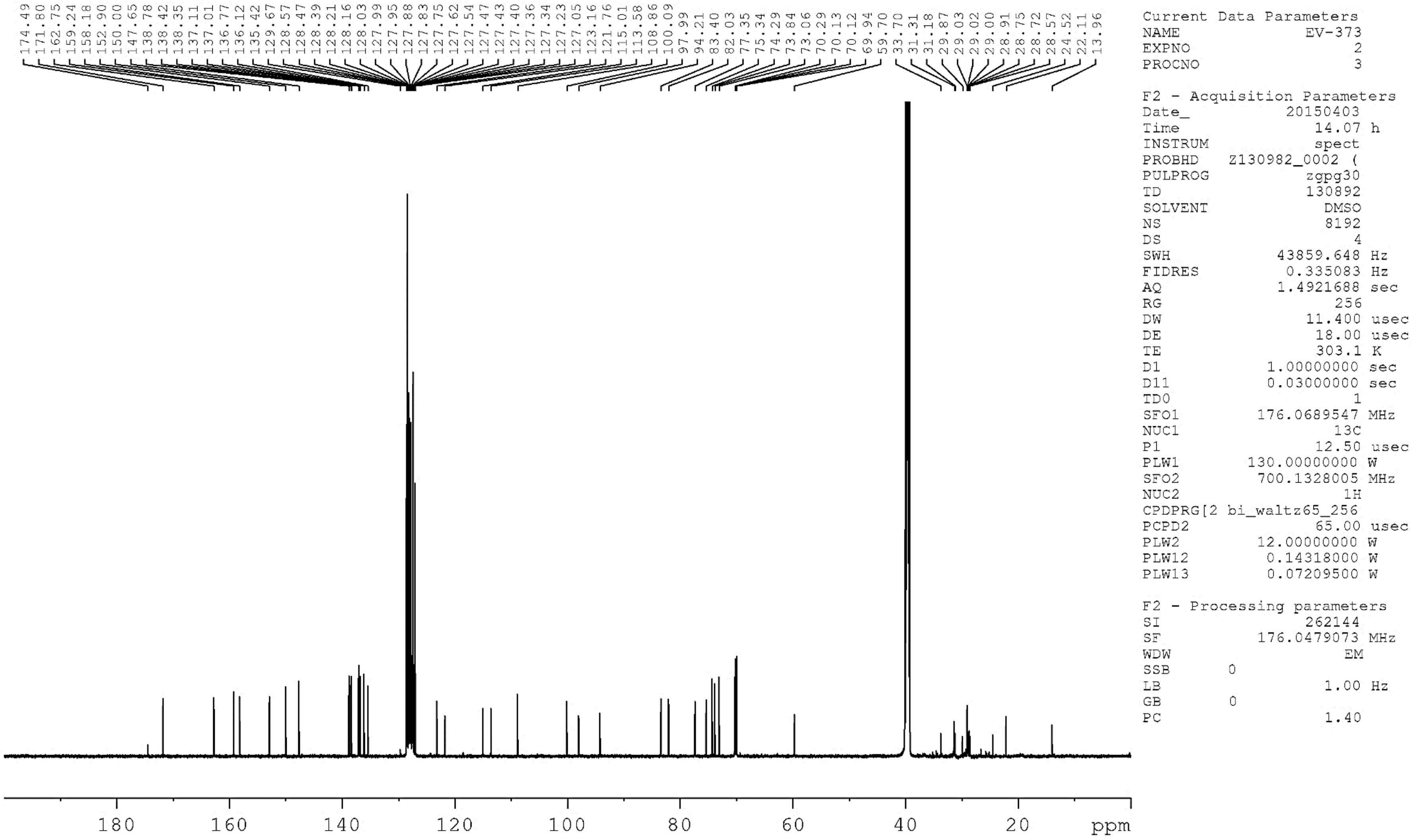

Figure S26. ^{13}C NMR spectrum of compound **34** (DMSO-d_6, 303.2 K).

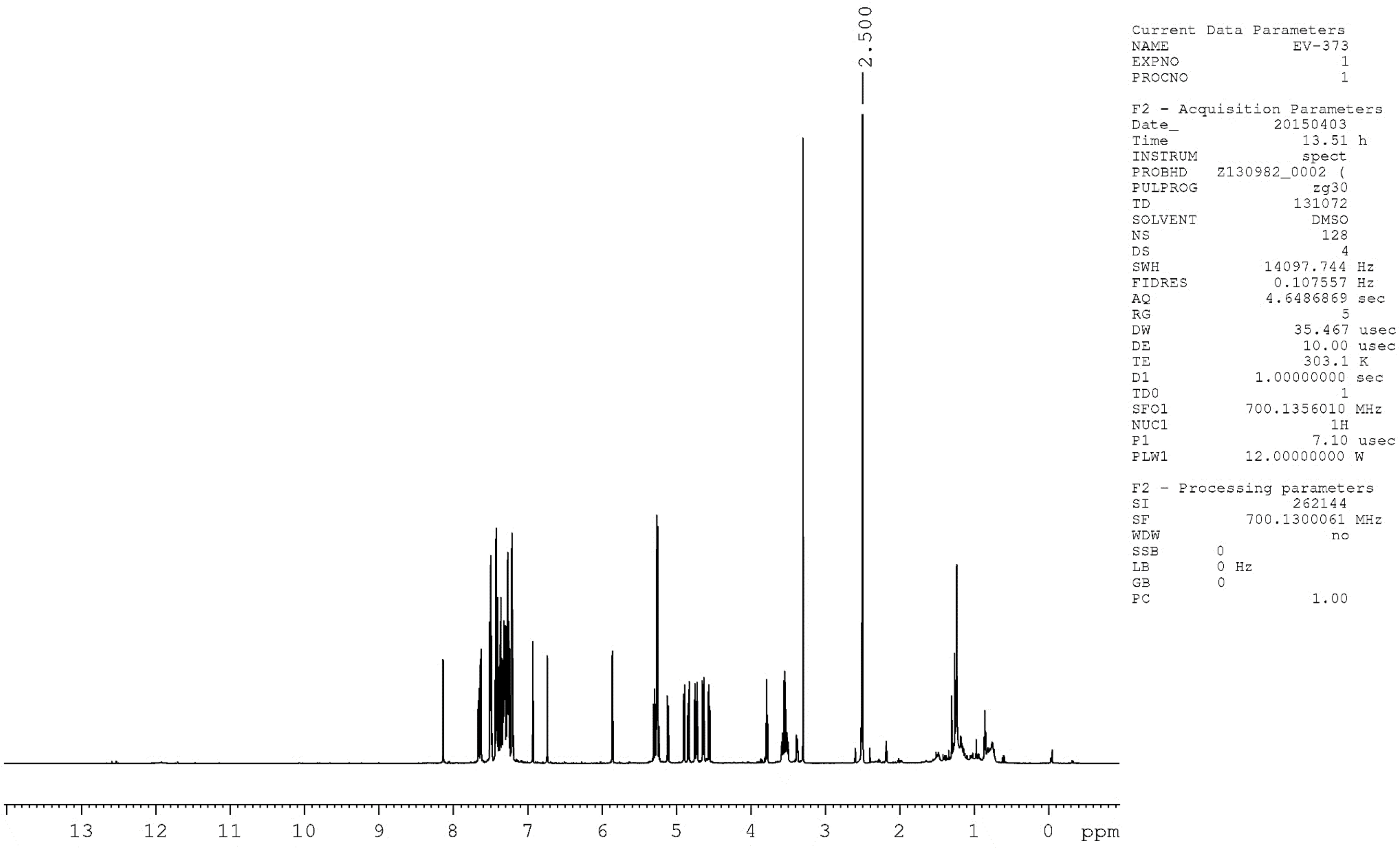

Figure S27. ^{1}H NMR spectrum of compound **34** (DMSO-d_6, 303.2 K).

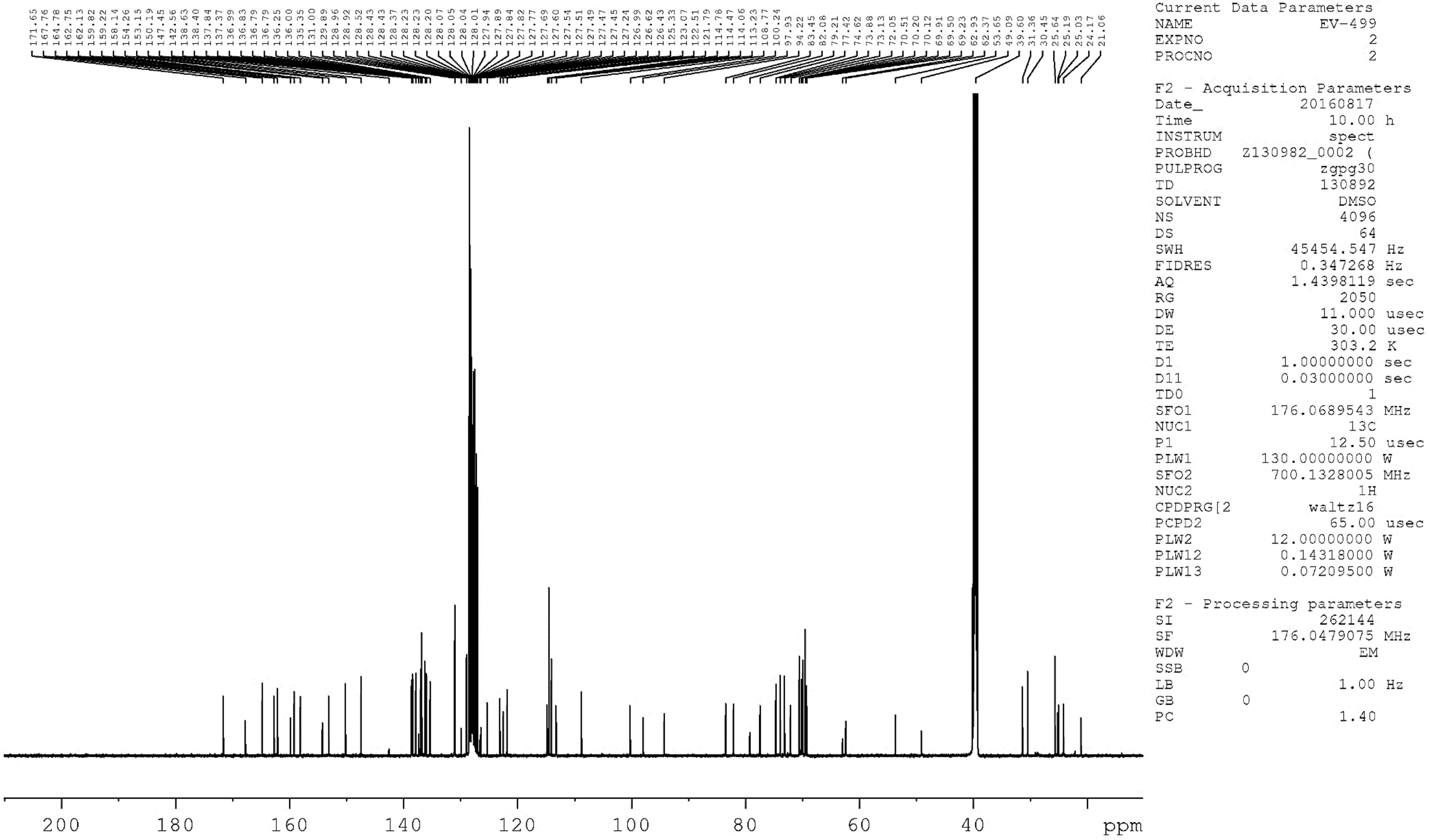

Figure S28. ^{13}C NMR spectrum of compound **35** (DMSO-d_6, 303.2 K).

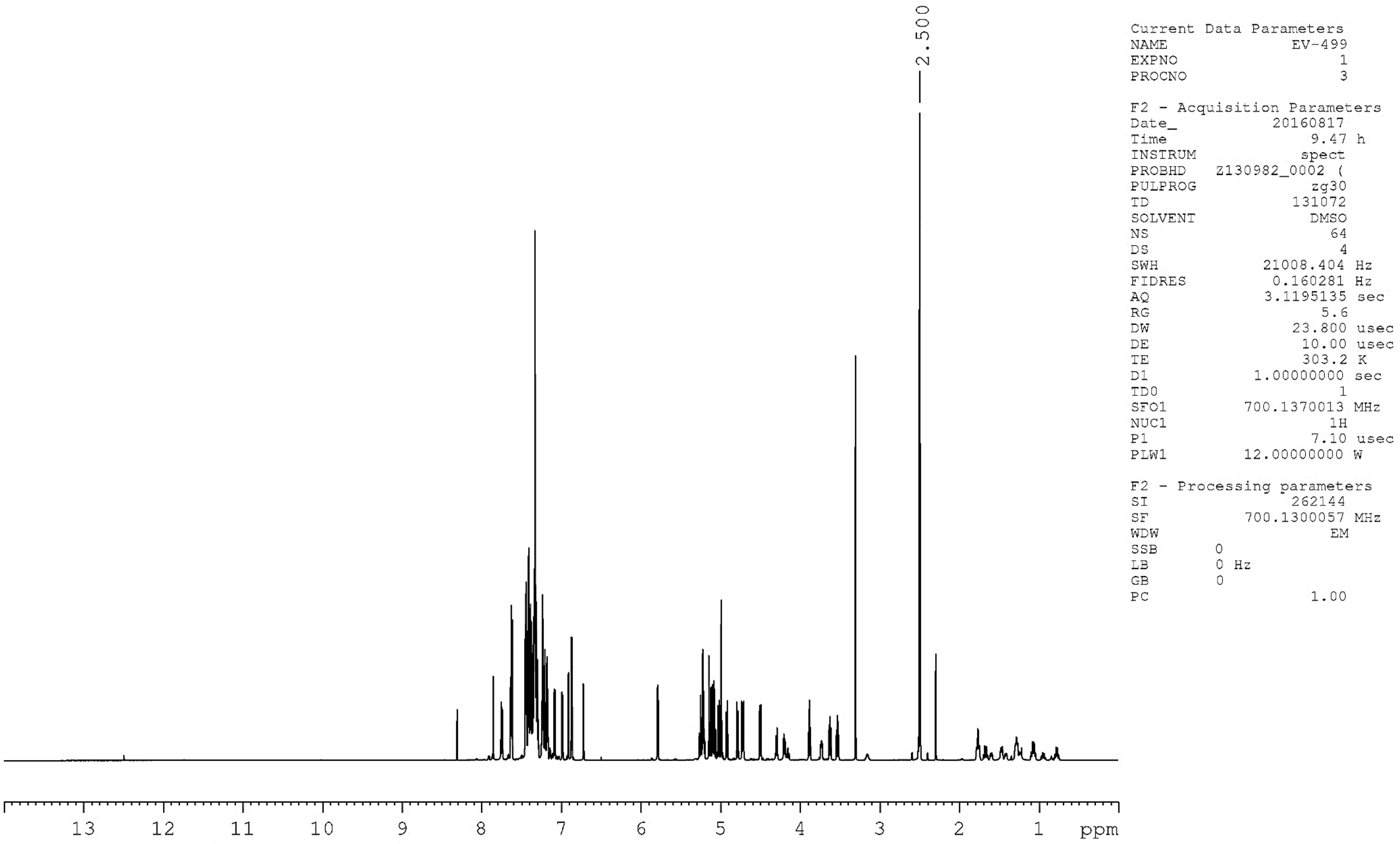

Figure S29. ^{1}H NMR spectrum of compound **35** (DMSO-d_6, 303.2 K).

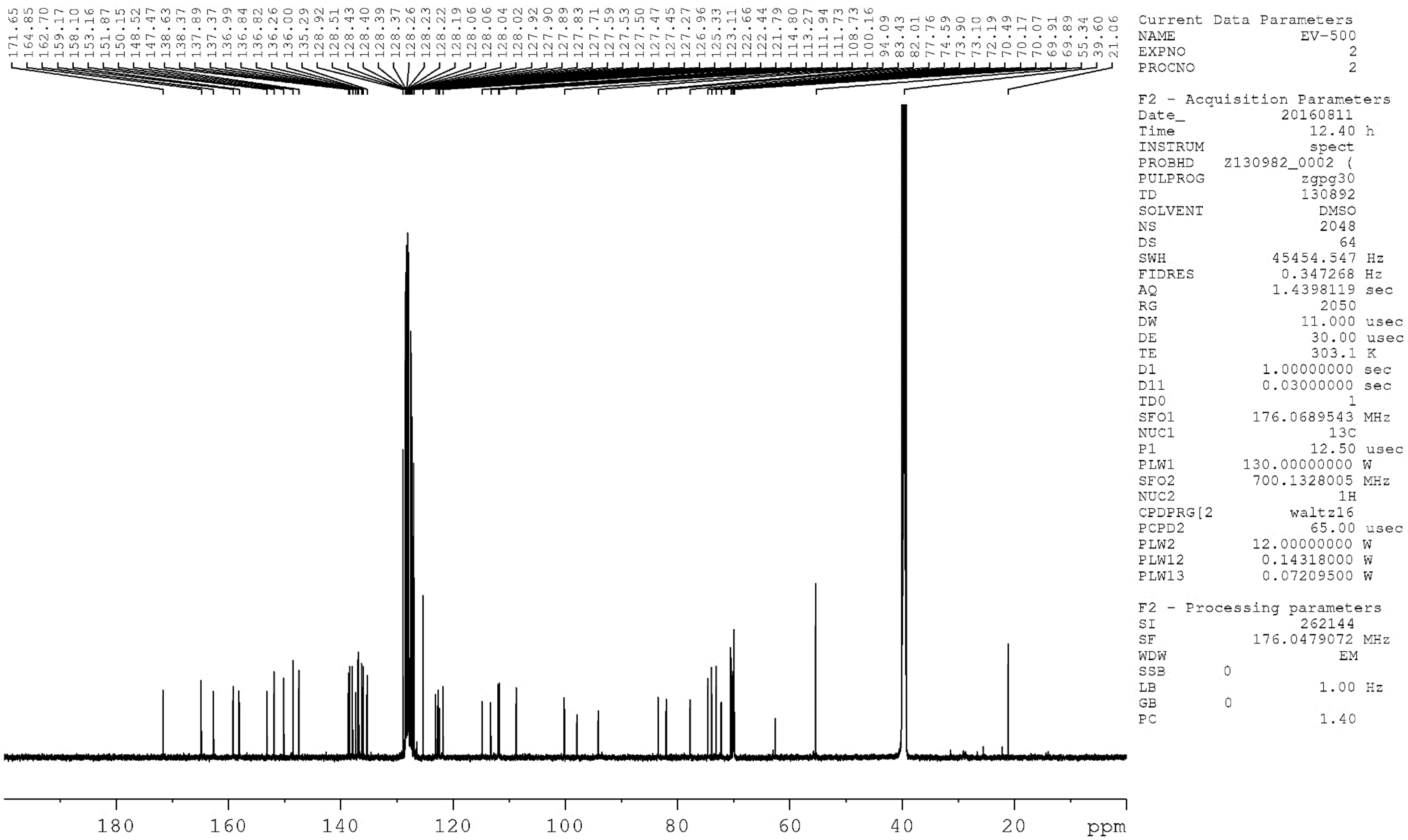

Figure S30. ^{13}C NMR spectrum of compound **36** (DMSO-d_6, 303.2 K).

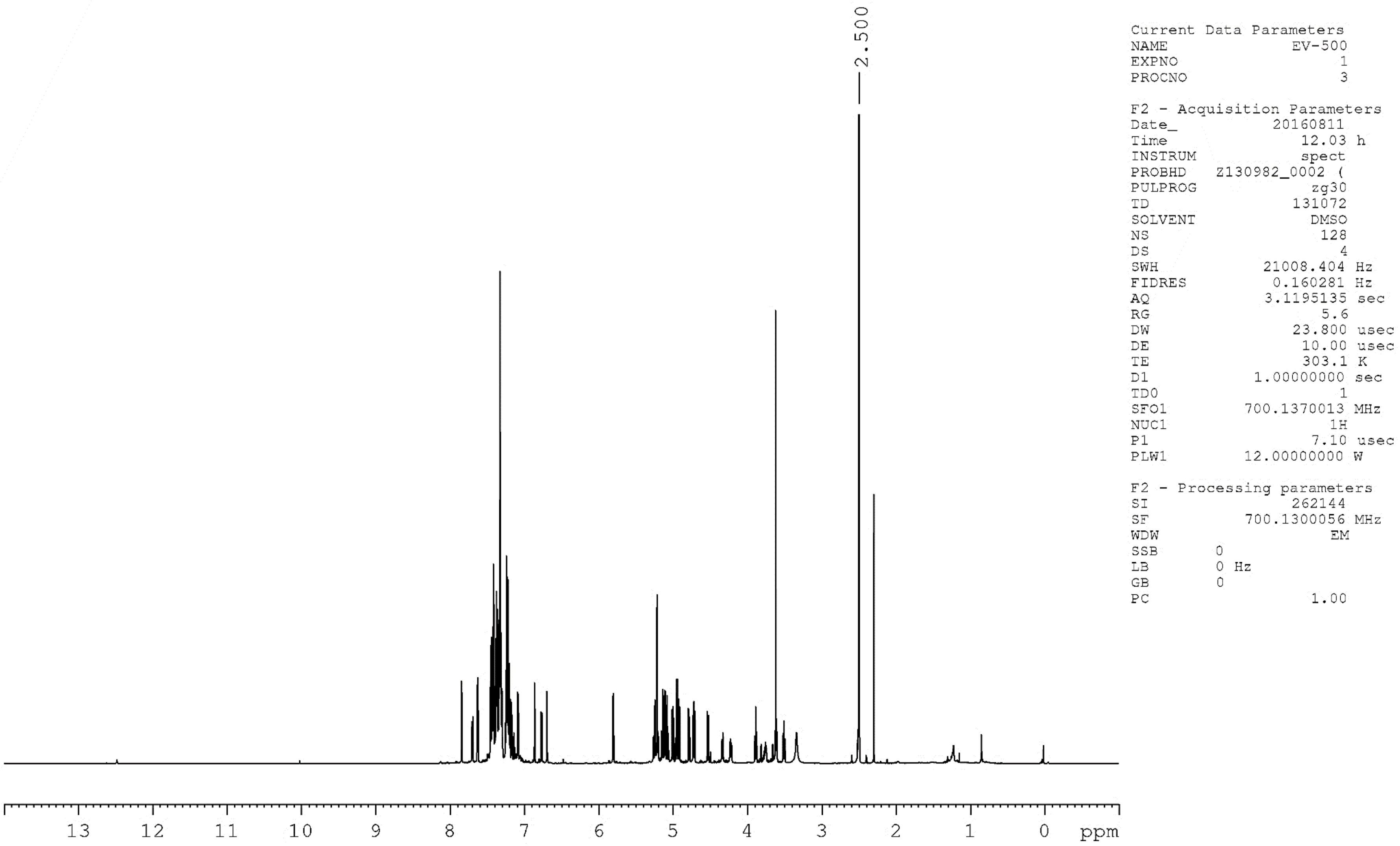

Figure S31. ^{1}H NMR spectrum of compound **36** (DMSO-d_6, 303.2 K).

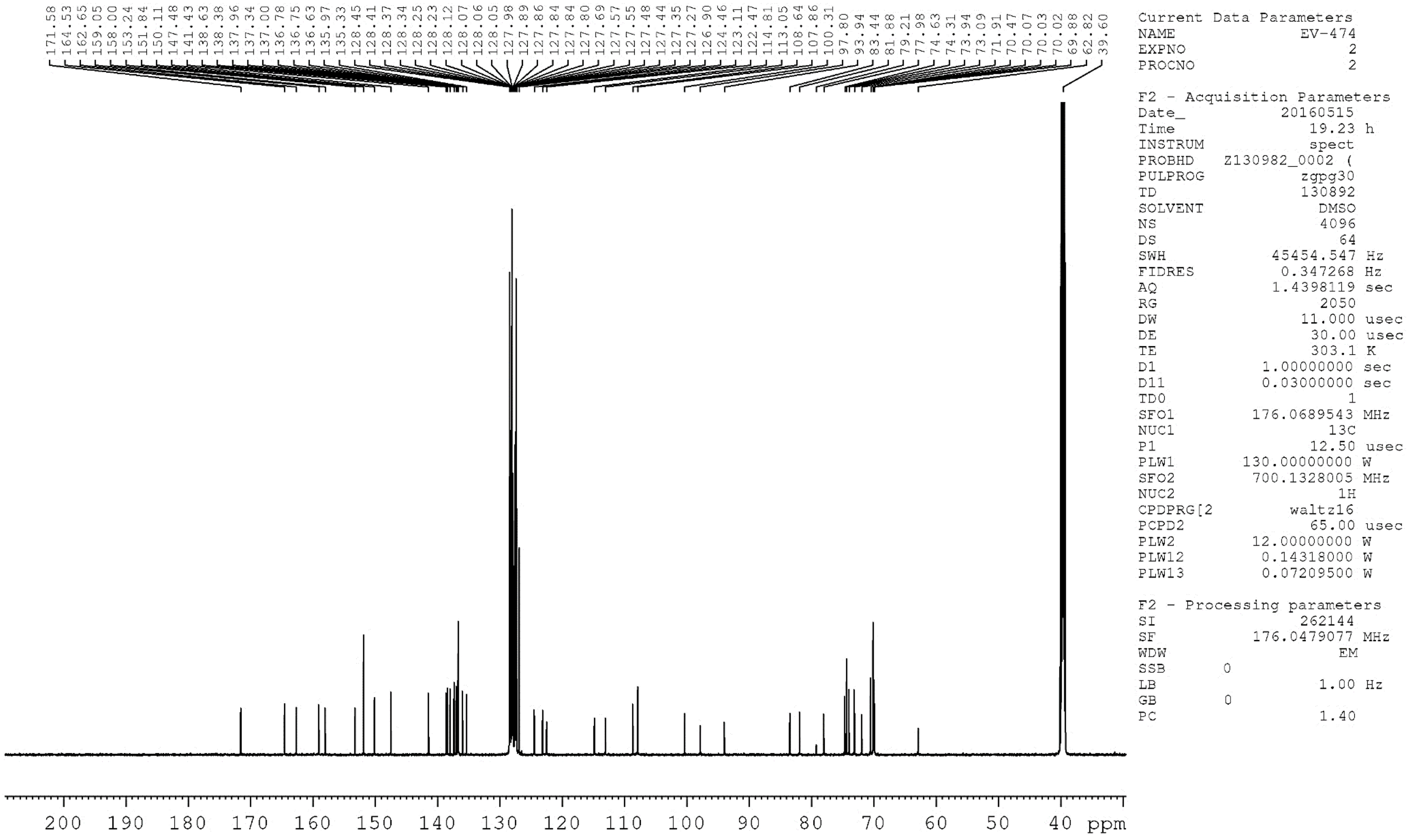

Figure S32. ^{13}C NMR spectrum of compound **37** (DMSO-d_6, 303.2 K).

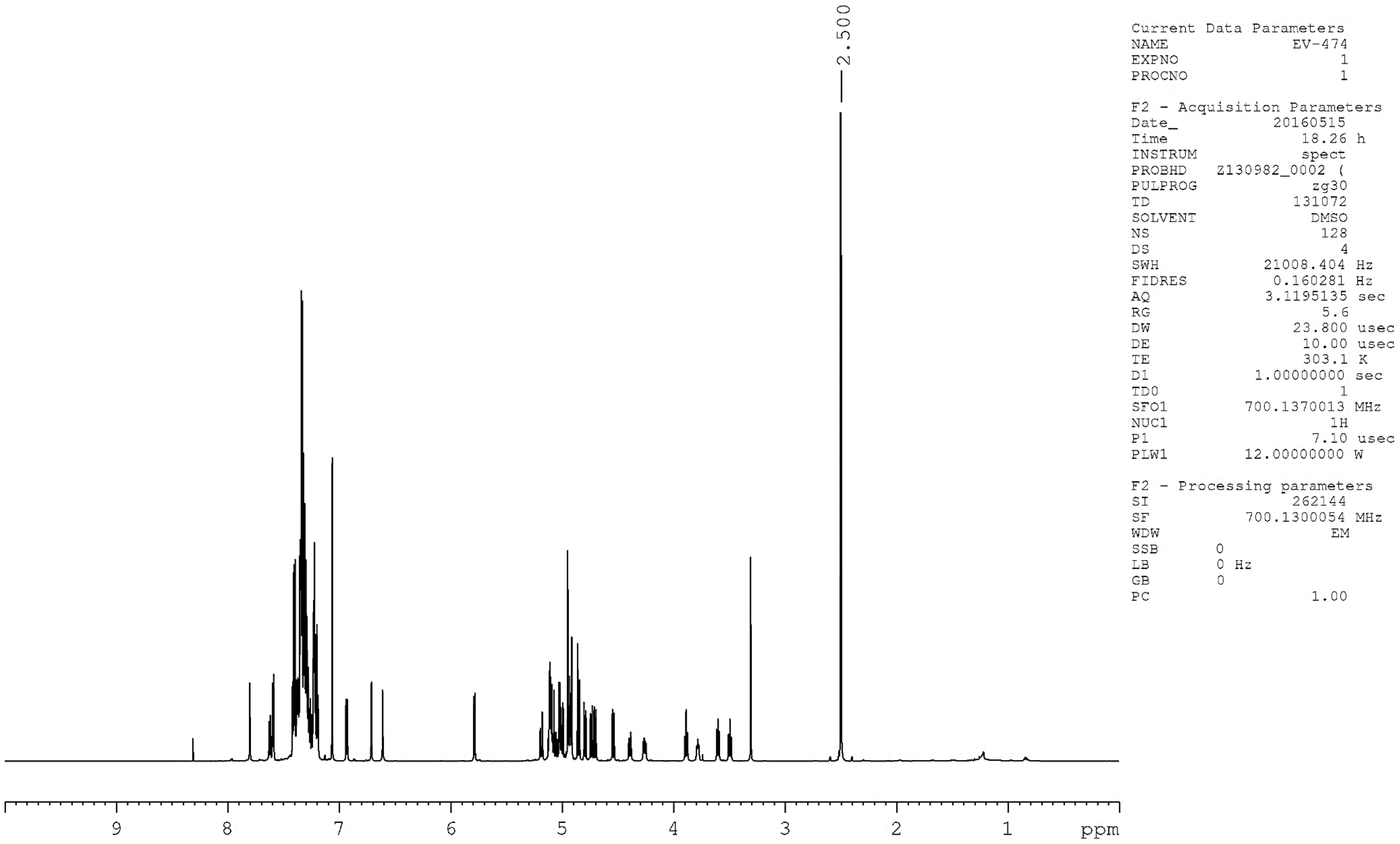

Figure S33. ^{1}H NMR spectrum of compound **37** (DMSO-d_6, 303.2 K).

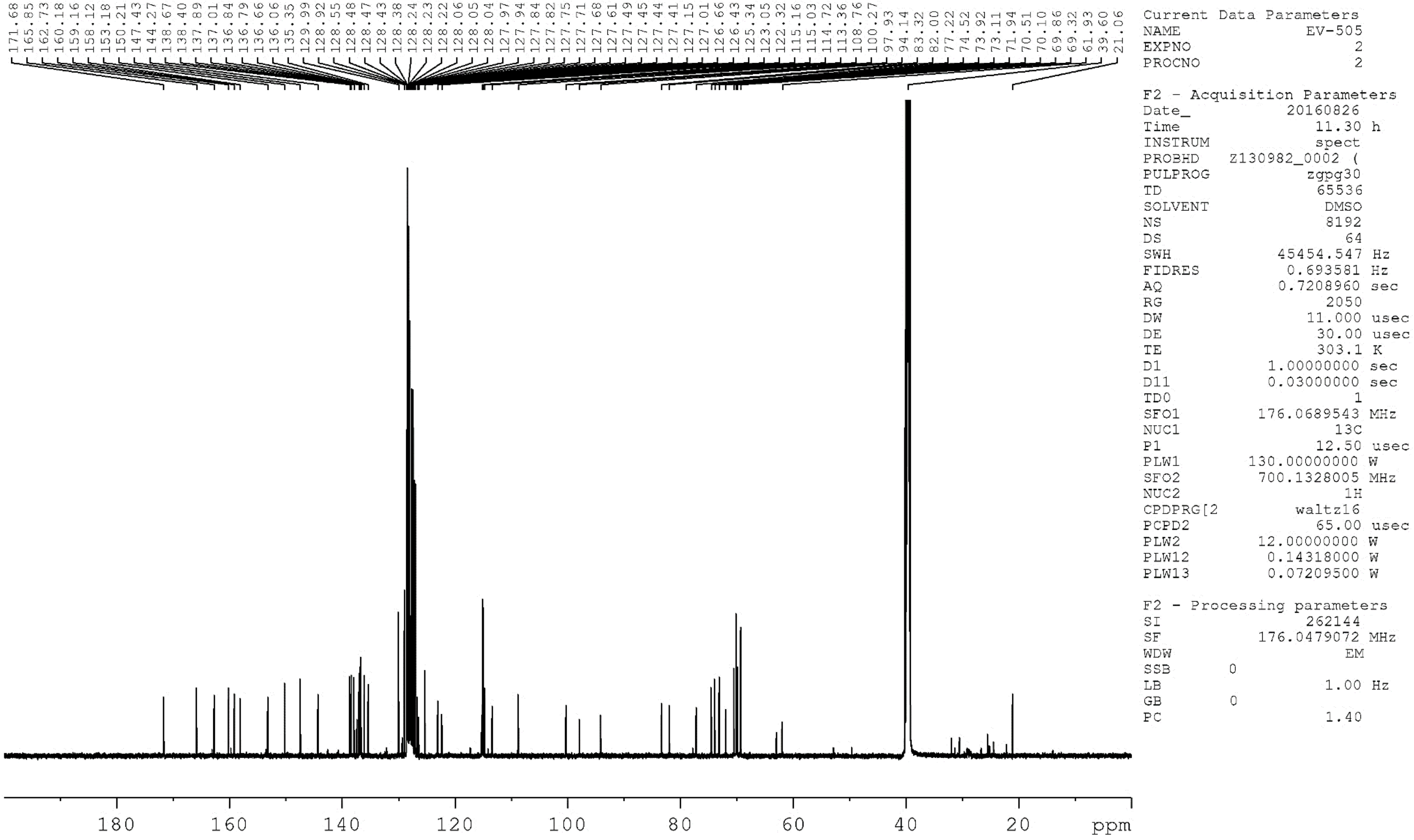

Figure S34. ^{13}C NMR spectrum of compound **38** (DMSO-d_6, 303.2 K).

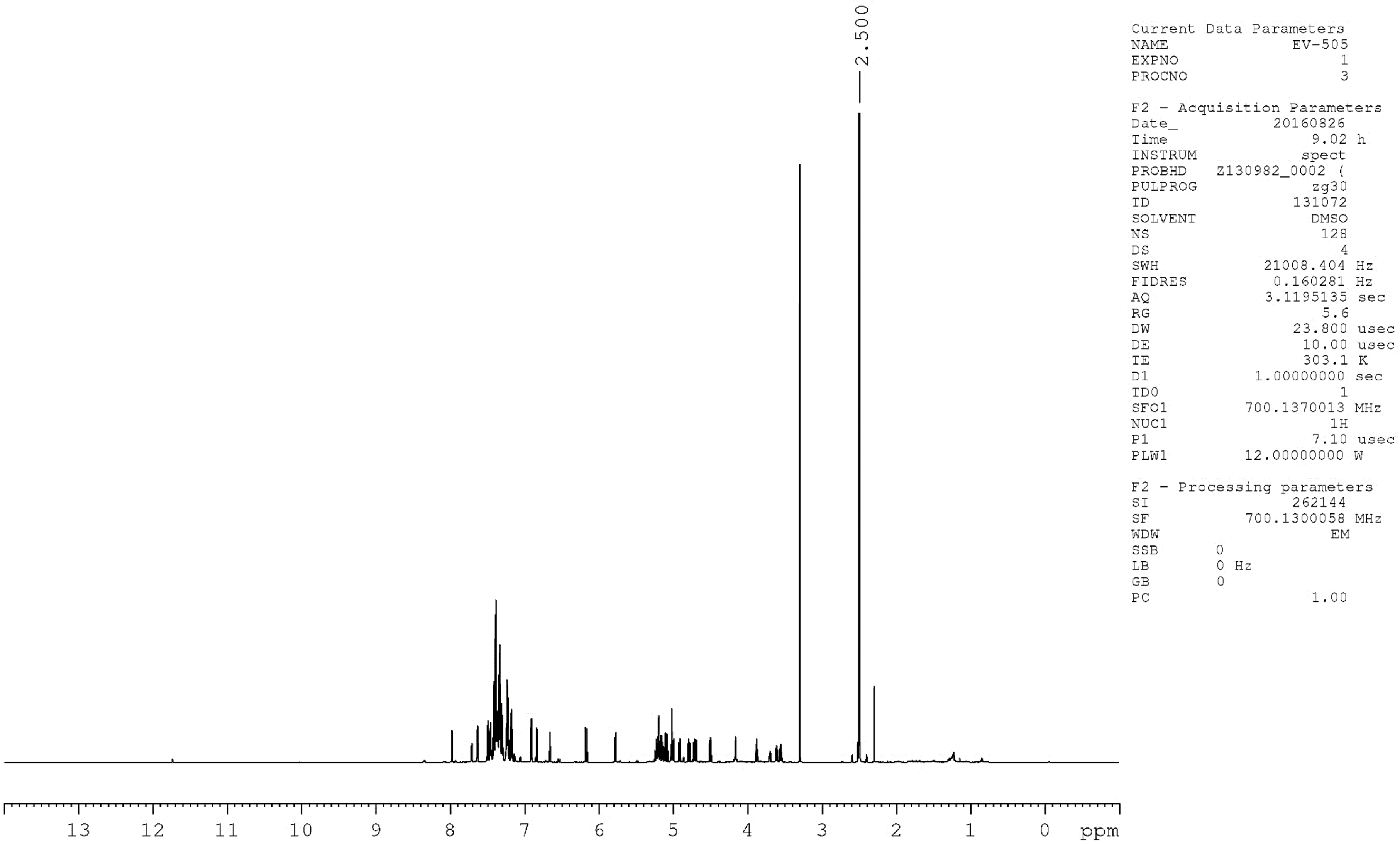

Figure S35. ^{1}H NMR spectrum of compound **38** (DMSO-d_6, 303.2 K).

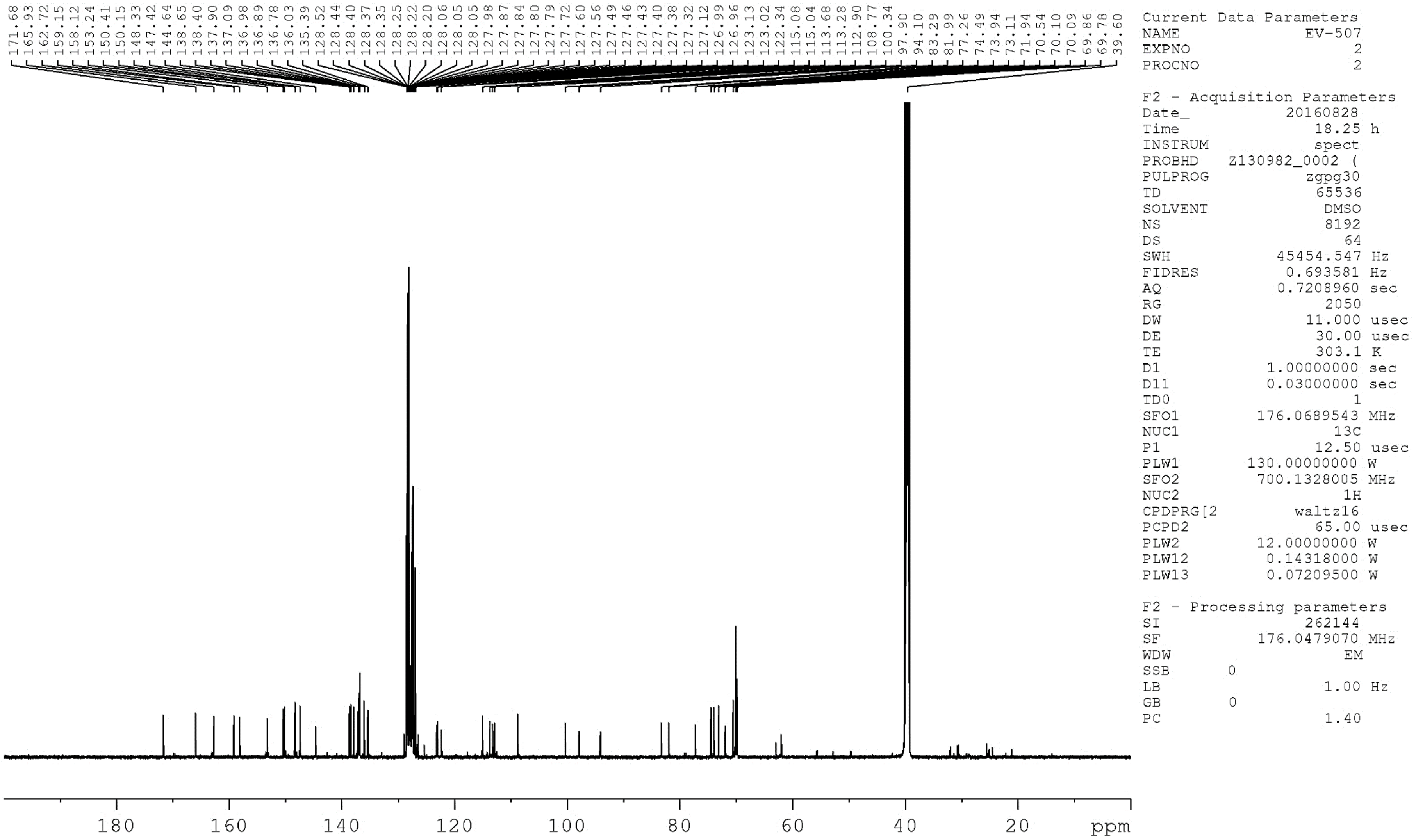

Figure S36. ^{13}C NMR spectrum of compound **39** (DMSO-d_6, 303.2 K).

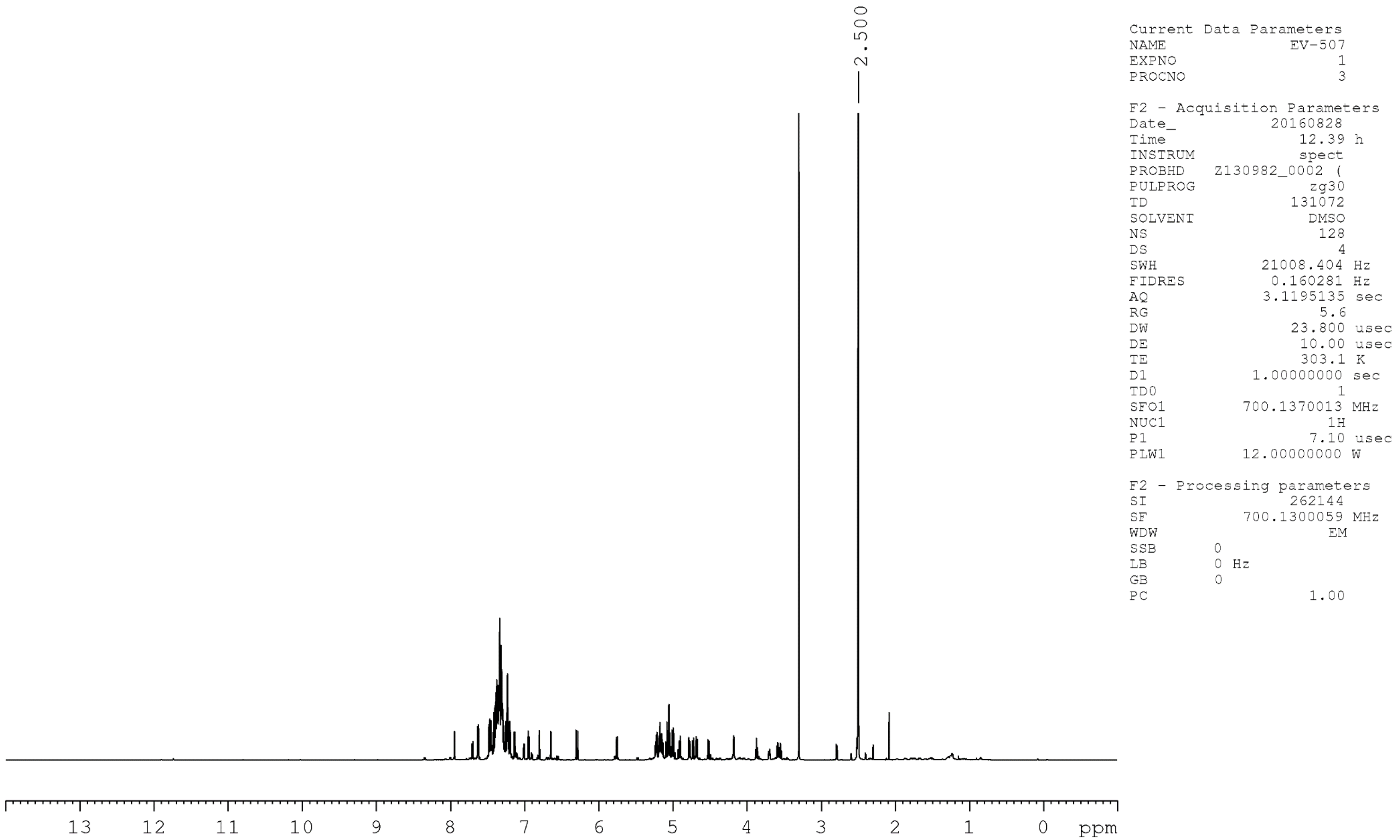

Figure S37. ^{1}H NMR spectrum of compound **39** (DMSO-d_6, 303.2 K).

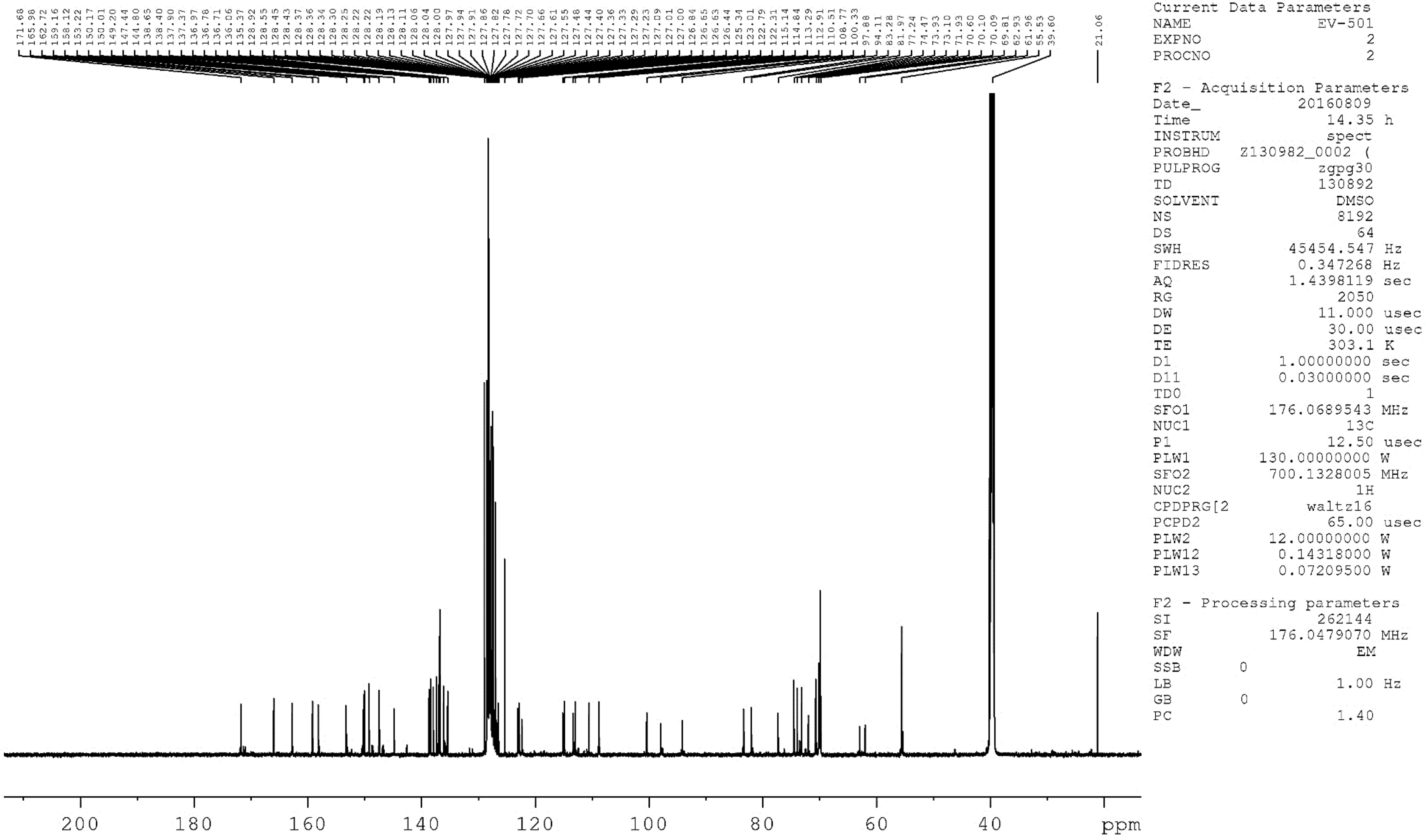

Figure S38. ^{13}C NMR spectrum of compound **40** (DMSO-d_6, 303.2 K).

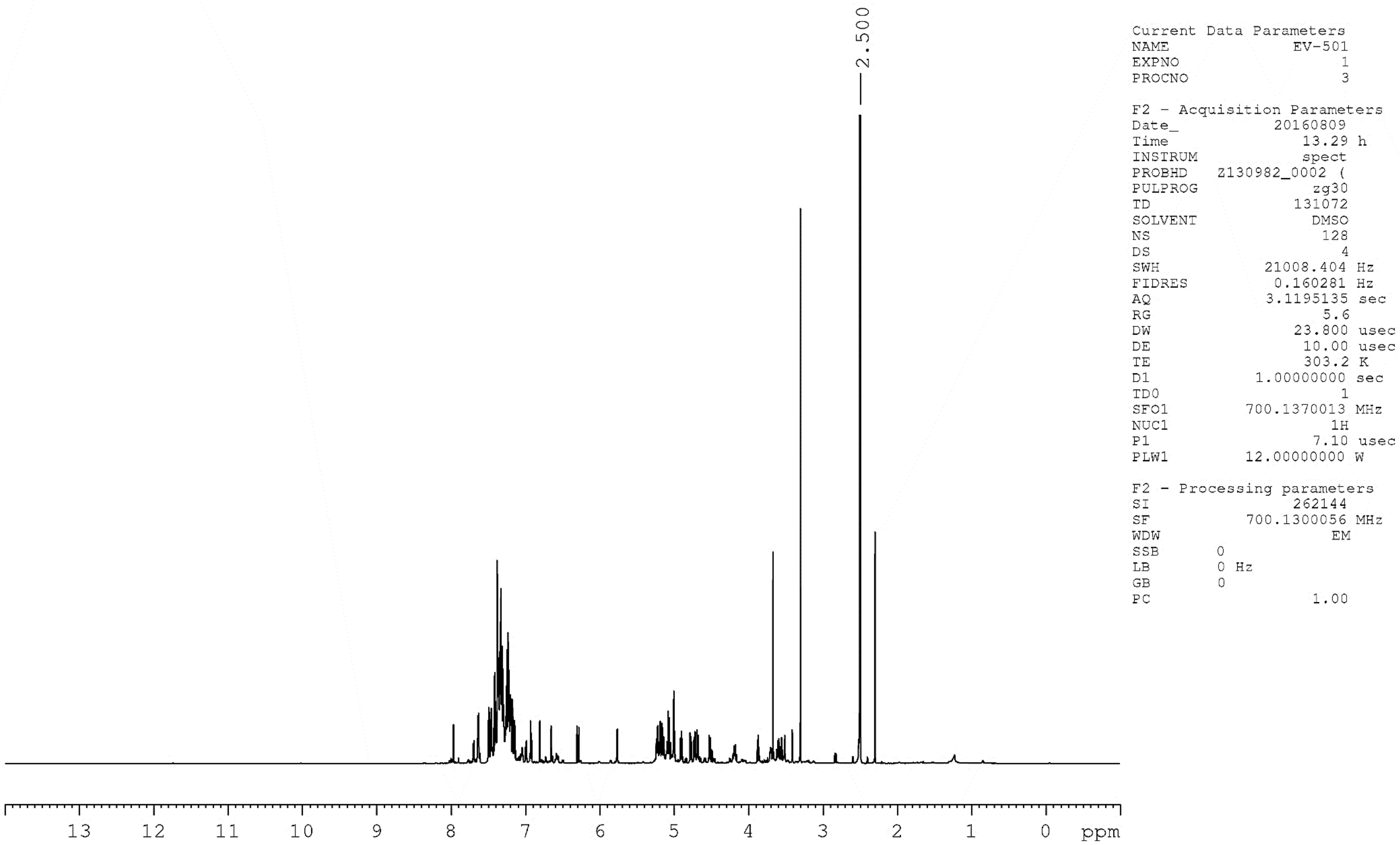

Figure S39. ^{1}H NMR spectrum of compound **40** (DMSO-d_6, 303.2 K).
